# Dopamine Homeostasis Imbalance and Dopamine Receptors-Mediated AC/cAMP/PKA Pathway Activation are Involved in Aconitine-Induced Neurological Impairment in Zebrafish and SH-SY5Y Cells

**DOI:** 10.3389/fphar.2022.837810

**Published:** 2022-03-18

**Authors:** Jie Zhou, Cheng Peng, Qiuju Li, Xiaoyu Yan, Liang Yang, Mengting Li, Xiaoyu Cao, Xiaofang Xie, Dayi Chen, Chaolong Rao, Sizhou Huang, Fu Peng, Xiaoqi Pan

**Affiliations:** ^1^ State Key Laboratory of Southwestern Chinese Medicine Resource, School of Pharmacy and School of Public Health, Chengdu University of Traditional Chinese Medicine, Chengdu, China; ^2^ Department of Pharmacy, The Affiliated Hospital of Southwest Medical University, Luzhou, China; ^3^ Development and Regeneration Key Laboratory of Sichuan Province, Department of Anatomy and Histology and Embryology, School of Basic Medicine, Chengdu Medical College, Chengdu, China; ^4^ West China School of Pharmacy, State Key Laboratory of Biotherapy, West China Hospital, Sichuan University, Chengdu, China

**Keywords:** aconitine, dopamine homeostasis, dopamine receptor, AC/cAMP/PKA, neurological impairment

## Abstract

Aconitine is one of the main bioactive and toxic ingredients of *Aconitum* species. Increasingly, aconitine has been reported to induce neurotoxicity. However, whether aconitine has effects on the dopaminergic nervous system remains unclear. In this study, zebrafish embryos at 6-days postfertilization were exposed to aconitine at doses of 0.5, 1, and 2 μM for 24 h, and SH-SY5Y cells were treated with 50, 100, and 200 μM of aconitine for 24 h. Results demonstrated that aconitine treatment induced deformities and enhanced the swimming behavior of zebrafish larvaes. Aconitine exposure suppressed cell proliferation and increased the number of reactive oxygen species and apoptosis in zebrafish larvaes and SH-SY5Y cells. Aconitine altered the levels of dopamine and its metabolites by regulating the expression of genes and proteins related to dopamine synthesis, storage, degradation, and reuptake *in vivo* and *in vitro*. Moreover, aconitine activated the AC/cAMP/PKA pathway by activating the dopamine D1 receptor (D1R) and inhibiting the dopamine D2 receptor (D2R) to disturb intracellular calcium homeostasis, eventually leading to the damage of nerve cells. Furthermore, the D1R antagonist SCH23390 and D2R agonist sumanirole pretreatment effectively attenuated the excitatory state of larvaes. Sumanirole and PKA antagonist H-89 pretreatment effectively decreased intracellular Ca^2+^ accumulation induced by aconitine *in vivo*. SCH23390 and sumanirole also reduced aconitine-induced cytotoxicity by inhibiting the AC/cAMP/PKA pathway *in vitro*. These results suggested that dopamine homeostasis imbalance and dopamine receptors (DRs)-mediated AC/cAMP/PKA pathway activation might be vital mechanisms underlying aconitine-induced neurological injury.

## Introduction

The plants of *Aconitum* genus have been extensively used to treat various diseases, such as heart failure, arrhythmia, and rheumatoid arthritis. Clinical research demonstrated that *Aconitum* species had various pharmacological activities, including heart function enhancement, anti-inflammation, analgesic action, and anti-tumor activity (Zhao et al., 2012; Zhang et al., 2017; Jie Yang et al., 2021). However, these plants have attracted increasing attention due to their cardiotoxicity and neurotoxicity (Peng et al., 2020; Jie Zhou et al., 2021; Wei Zhou et al., 2021; Ye et al., 2021). Palpitation, arrhythmia, dizziness, numbness, stiffness or convulsions of tongue and limbs, paresthesia, and general weakness are considered the typical symptoms of *Aconitum* poisoning (Chung et al., 2021). Aconitine, a diester diterpene-type aconitum alkaloids (DDAs), is one of the main bioactive and toxic ingredients in Fuzi and other *Aconitum* plants. Poisoning events caused by the irrational use of *Aconitum* containing aconitine occur frequently. However, the neurotoxic mechanisms of aconitine remain poorly understood. It is quite valuable to elucidate the neurotoxic mechanisms of aconitine to identify controllable targets for the detoxification of *Aconitum*.

In the present studies, aconitine-induced neurotoxicity possibly involves disturbance of ion homeostasis, induction of oxidative stress and apoptosis, mitochondrion injury, interference with energy metabolism, and alteration of neurotransmitter levels (Yamanaka et al., 2002; Peng et al., 2009; Zhao et al., 2010; Chen et al., 2021; Liang Yang et al., 2021). The typical clinical features of aconitine poisoning involved involuntary tremors and progressive dyskinesia (Pan and Peng, 2017). Previous studies showed that *Aconitum* could significantly evoke dopamine release which induced oxidative stress and dysfunction of dopaminergic neurons (Zhao et al., 2010). As a monoamine neurotransmitter, dopamine plays an essential role in a variety of physiological processes, such as motor activity, sensory control, decision making, learning, and memory (Runegaard et al., 2019). Several functional proteins, such as tyrosine hydroxylase (TH), monoamine oxidase (MAO), dopamine transporter (DAT), and vesicular monoamine transporter type 2 (VMAT2), have a critical role in maintaining dopamine homeostasis from the cytoplasm to the synaptic cleft. The selective binding between the synaptic dopamine and dopamine receptors (DRs) in the postsynaptic membrane is the key to regulating neuronal excitability and neuronal cell survival (Blanco-Lezcano et al., 2018). DRs that have five subtypes belong to the G-protein-coupled receptors (GPCRs) superfamily. D1-like receptors (containing D1R and D5R) are generally coupled to Gs/olf and enhance intracellular cAMP level and PKA activity, whereas D_2_-like receptors (including D2R, D3R, and D4R) are coupled to G_i/o_ and inhibit the production of cAMP, resulting in a decrease of PKA activity (Beaulieu and Gainetdinov, 2011). The downstream pathways mediated by PKA can affect the excitability and survival of nerve cells (Saavedra et al., 2002; Nagai et al., 2016). These studies suggest that dopaminergic signalling regulation may be an important mechanism of aconitine-induced neurotoxicity. However, few studies have evaluated the effects of aconitine on the dopaminergic signalling pathway.

Zebrafish has up to 70% homology to human genes, of which biological structure, physiological function, and signal transduction are highly similar to mammals (Howe et al., 2013). Although the brain tissue of zebrafish is simple, their embryos develop rapidly and the nervous system has matured on 5 days postfertilization (dpf) (Schweitzer and Driever, 2009). Overall, zebrafish has emerged as a promising and ideal model to investigate the mechanisms of drug-induced neurotoxicity (Chen et al., 2021). In this study, we employed zebrafish larvaes and SH-SY5Y cells to quantify the effects of aconitine on morphology, neurobehavior and cell viability. This study also aimed to determine whether and how aconitine interfered with dopamine homeostasis.

## Materials and Methods

### Reagents

Aconitine (purity>98%) was purchased from Chengdu Must Bio-Technology Co., Ltd (China) and prepared with dimethyl sulfoxide (DMSO) (Shanghai Solarbio Bioscience and Technology Co., Ltd, China) into the mother liquor with a concentration of 200 mM. Dopamine and its metabolites, namely 3, 4-dihydroxyphenylacetic acid (DOPAC) and homovanilic acid (HVA) standards (more than 97% purity), were obtained from Sigma (United States). D1R antagonist SCH23390, D2R agonist sumanirole, and PKA antagonist H-89 were provided by MCE (United States). Anti-TH was acquired from Sigma-Aldrich (United States). Primary antibodies against MAO-B, VMAT2, and D1R were obtained from Abcam (England). Primary antibodies against D2R, DAT, and adenylate cyclase (AC) were purchased from Santa Cruz Biotechnology (United States). PKA, p-PKA, and β-actin rabbit monoclonal antibodies were obtained from Cell Signaling Tech (Danvers, MA, United States). Trizol for RNA extraction was acquired from Invitrogen (United States). All primers for PCR were provided by Biological Engineering Technology Company (Shanghai, China). First-strand cDNA Synthesis kit and SYBR Green Quantitative kits for real-time polymerase Chain Reaction (RT-PCR) were purchased from Tiangen Biotech Co., Ltd. (Beijing, China).

### Zebrafish Preparation

Wild type AB zebrafish (*Danio rerio*) were purchased from the China Zebrafish Resource Center. *Tg* (*vmat2:GFP*) transgenic zebrafish were provided by S. Huang’s laboratory. Adult zebrafish were bred in accordance with the routine procedures of the zebrafish breeding center at Chengdu University of Traditional Chinese Medicine (Li et al., 2020). The females and males (1:2) were placed in a breeding tank overnight and were initially separated by partitions that were removed next morning after 30 min of illumination. After the removal of the dead eggs and faecal residual, the fertilized eggs were collected and transferred to a sterile culture dish. Zebrafish larvaes at six dpf were used for the following experiments. The animal experiments involved in this study were approved by the Animal Research Ethics Committee of Chengdu University of Traditional Chinese Medicine (SYXK 2016-04).

### Acute Toxicity Assay of Zebrafish Larvaes

Aconitine was diluted with fish-raising water to different concentrations with less than 0.1% DMSO in each group. Larvaes at six dpf were placed in a 24-well plate with the density of 10 embryos per well, and were exposed to 0.937, 1.875, 3.75, 7.5, 15, or 30 μM of aconitine for 24 h, respectively. Three parallel samples were set for each concentration following the similar criteria with the vehicle controls. The fish with no visible movement (such as the flapping of gills, no response to touching the tail) was dead, and the dead larvaes in each group was collected to calculate the median lethal concentration (LC50) value of aconitine on zebrafish larvaes.

### Morphological Defects of Zebrafish Larvaes

According to the LC50 of aconitine, the larvaes were treated with 0.5, 1, and 2 μM of aconitine in morphological observation and the following experiments. Potential teratogenicity of aconitine was estimated by monitoring the morphological defects of larvaes at seven dpf under a stereomicroscope. Several morphological indices, including body length (μm), ocular distance (μm), and surface area of the eyes (μm^2^), were evaluated using ImageJ.

### TUNEL Assay of Zebrafish Larvaes

TMR (red)-TUNEL cell apoptosis detection kit (Servicebio, China) was used to investigate whether aconitine induces the apoptosis of neuronal cells in zebrafish larvaes (Quan et al., 2019). Larvaes at six dpf were treated with aconitine following the methods in Section 2.4. The larvaes were fixed overnight at 4°C in 4% paraformaldehyde (PFA). After washing with PBS, the larvaes were orderly dehydrated by 95%, 90%, 80%, and 70% ethanol for 5 min each and then stored in 100% methanol overnight at -20°C. The larvaes were rehydrated and treated with proteinase K (20 μg/ml) at 37°C for 25 min, and then washed three times with PBS. The samples were added with 0.1% triton to permeabilize the tissues for 20 min at room temperature. The larvaes were then incubated at 37°C in a TUNEL reaction mixture containing TDT enzyme, dUTP, and buffer (1:5:50) for 2 h. After incubation, the nuclei were counterstained with DAPI at room temperature for 10 min. The larvaes were washed with PBS (three times, 5 min each) and covered with an anti-fade mounting medium. Apoptotic cells staining red and nuclei staining blue were observed under a fluorescence microscope.

### Locomotor Behavioral Test

Zebrafish larvaes at six dpf were transferred into 96-well plates (one fish/well) and treated with 100 μl of aconitine (0.5, 1, or 2 μM) per well for 24 h. After 0.5, 3, 6, 12, and 24 h, the swimming behavior of larvaes was monitored by the automated video tracking system (Noldus Co., Netherlands). After 5 min of habituation without light, the behavioral trajectories of larvaes for 10 min were captured and used the Ethovision XT12 software (Noldus Co., Netherlands) to track the swimming activity at a rate of 15 positions per second. Total distance travelled, movement speed, movement time, angular velocity, meander, clockwise (CW) rotation, frequency of visits to edge zone, and the behavioral trajectories were also obtained through the Ethovision XT12 software.

### Cell Culture and Treatment

Human neuroblastoma (SH-SY5Y) cells were purchased from Procell Life Science and Technology (Wuhan, China) and routinely cultured in the mixed medium of MEM: F12 (1:1) containing 10% fetal bovine serum (Gibco) and 1% penicillin-streptomycin (Sigma) at 37°C with 5% CO_2_. The culture medium was renewed once every 2 days. Aconitine was diluted with less than 0.1% DMSO to 25–400 μM of concentration range for the following experiments.

### Cell Viability Measurement

Cell viability was performed using the CCK-8 assay kit (Biosharp Life Sciences, China) according to the protocol. The cells (5×10^3^ cells/well) were seeded in a 96-well plate. After treatment with different concentrations of aconitine (25, 50, 100, 150, 200, 300, and 400 μM) for 24 or 48 h, the cells were incubated with 10 μl of CCK-8 solution for 2 h in the dark at 37°C. The absorbance was determined at 450 nm under an automated microplate spectrophotometer (Spectra Max, MD, United States).

### Quantification of Cell Apoptosis by Flow Cytometry

The Annexin V-FITC/PI double staining kit (Solarbio Life Sciences, China) was performed to quantify cell apoptosis according to the manufacturer’s instruction. The cells were incubated with aconitine at doses of 50, 100, and 200 μM for 24 h, and washed twice with cold PBS. The collected samples were centrifuged at 300 g for 5 min, and the supernatant was removed. The cells were resuspended with the binding buffer at a concentration of 1 × 10^6^ cells/mL. The cell suspensions were added with 5 μl of Annexin V-FITC, mixed gently, and incubated for 10 min at room temperature in the dark. All samples were in the dark incubated for 5 min at room temperature after adding 5 μl of PI solution. Annexin V-FITC binding and PI staining were evaluated by a flow cytometer (Ex = 488 nm, Em = 530 nm) (FACSVerse, BD Biosciences, United States). Total 10,000 events were analyzed for each independent experiment.

### Determination of Reactive Oxygen Species (ROS)

DCFH-DA staining was used to determine intracellular ROS levels *in vivo* and *in vitro*. The larvaes were placed in a 12-well plate with athe density of 10 fishes per well, and incubated with 0.5, 1, and 2 μM of aconitine for 24 h. The probe solution was diluted following the instructions. After staining with 25 μM of working solution (freshly prepared) at 28°C for 1 h in the dark, zebrafish larvaes were washed with fresh fish water three times and were taken photos under a stereoscopic fluorescence microscope (Leica, M165 FC, Singapore). *In vitro*, SH-SY5Y cells in each group were treated with aconitine at doses of 50, 100, and 200 μM for 24 h. The cells were incubated with 10 μM working solution prepared by serum-free medium for 1 h at 37°C. After the cells were gently washed with PBS two times, the fluorescence images were captured under a laser scanning confocal microscope (Leica TCS-SP8 SR, Germany). The intensity of fluorescence was analyzed with the ImageJ software.

### High-Performance Liquid Chromatography Analysis of Dopamine and its Metabolites

Fifty larvaes per sample were diluted in 30 μl of PBS, and then added with 6 μl of 0.1 mM perchloric acids after tissue homogenization, following 10 min of incubation on ice. The homogenates were centrifuged at 4°C with 14,000 g for 15 min 400 µl of supernatant was transferred to 1.5 ml of Eppendorf tubes, in which the same amount of 0.1 M perchloric acid was added on ice for 10 min, following centrifugation at 14,000 *g* (4°C) for 15 min. The supernatant was filtered through a 0.22 μm microporous membrane and stored in a brown sample bottle for HPLC analysis. *In vitro*, the cells seeded in a new culture dish were treated with different concentrations of aconitine (50, 100, and 200 μM) for 24 h. The cells were collected and centrifuged after wash with PBS twice, following resuspension with 30 μl of PBS. The cell samples were collected following the similar methods with the larvae samples. Next, dopamine, DOPAC, and HVA standards with the concentration of 100 μg/ml and the calibration mixture were prepared with ultra-pure water. The calibration concentrations ranged from 0.5 ng/ml to 500 ng/ml of dopamine and DOPAC, and from 2 ng/ml to 2000 ng/ml of HVA. The levels of dopamine and its metabolites were measured by HPLC (Enayah et al., 2018). Ultimate 3,000 system (ThermoFisher, United States) equipped with a diode array detector was used for HPLC analysis. Chromatographic separation was performed using a ChromCore C18 chromatographic column (4.6 mm × 250 mm, 5 μm, NanoChrom Technologies, China). The mobile phase consisted of acetonitrile and 0.1% trifluoroacetic acid at the flow rate of 1 ml/min with the column temperature of 25°C. The results were expressed as a nanogram of analyte per milligram of protein in the homogenate. Total protein was measured by the BCA protein quantification kit. The ratio of DOPAC + HVA to dopamine contents was calculated to reflect dopamine turnover that is a specific index of dopamine metabolism (Thiffault et al., 2000).

### Zebrafish Larvaes Photographed by the Laser Scanning Confocal Microscope

*Tg* (*vmat2:GFP*) transgenic zebrafish embryos at one dpf were cultured in dishes with 0.03% 1-phenyl 2-thiourea (PTU) that inhibits the formation of melanin. The larvaes of seven dpf were anaesthetized (tricaine, 0.4%) and covered with 3% methylcellulose after different concentrations of aconitine treatment for 24 h. The zebrafish’s position was fixed and the brain of larvaes were photographed by LSCM (Olympus FV-1200, Japan).

### Whole-Mount Immunofluorescence Staining of Zebrafish Larvaes

Whole-mount immunofluorescence staining was used to observe the number of TH-positive cells in zebrafish larvaes. Wild type AB larvaes were treated with aconitine for 24 h, washed with cold PBS three times, and fixed with 2 ml of 4% formaldehyde overnight. After wash three times with TBST, the samples were punched with 1 ml of working fluid (1.25% NaOH: H_2_O_2_ = 4:1) for 20 min, added with 2.0 ml of methanol, and placed in a horizontal shaker for 15 min. The larvaes were blocked with 2% nonfat-dried milk (TBST +2% BSA) for 1 h, following incubation with the mouse-anti-TH antibody (1:500) at 4°C overnight. Alexa Fluor 488 labelled goat-anti-mouse antibody (1:200) was applied at room temperature for 1 h in the dark. The embryos were washed three times with TBST for total 30 min. The density of fluorescence was observed under LSCM.

### Measurement of Ca^2+^ in Zebrafish Larvaes

Fluo-4 AM, a fluorescent dye (Servicebio, China), is usually applied to detect cytosolic Ca^2+^. After aconitine treatment, the larvaes were incubated with 40 μM of Fluo-4 AM containing 0.8% DMSO in egg water at room temperature for 30 min in the dark. The larvaes were then washed three times with fresh fish water, and the images were photographed under LSCM.

### Western Blot Analysis

Zebrafish larvaes at six dpf were transferred to a 6-well plate (30 larvaes/well) and incubated with aconitine for 24 h. The larvaes were then washed twice with cold PBS and transferred to a new centrifuge tube. Each sample was added with 200 μl of RIPA containing PMSF and protease Inhibitor Cocktail (1:100) and homogenized for 2 min at -20°C with 70 Hz (Jingxin, China). The homogenates were centrifuged at 12,000 g, 4°C for 15 min, and the supernatant was collected. *In vitro*, SH-SY5Y cells were seeded in a culture dish as described previously. After aconitine administration, the cells were harvested and washed twice with cold PBS, lysed by RIPA on ice for 30 min, and centrifuged at 14,000 g, 4°C for 15 min. The supernatant was collected to 500 µl of centrifuge tube. After the protein was quantified with the BCA protein quantification method, the lysates were denatured with the loading buffer at 95°C for 5 min. An equal amount of protein (20–50 μg/well) was electrophoresed on 10% SDS-PAGE gel. The proteins were then electroblotted to PVDF membranes and blocked with 5% non-fat skim milk diluted in TBST (0.1% Tween/TBS) for 2 h. The membranes were then incubated with the primary antibody overnight, washed three times (5 min each time), and incubated again with the horseradish peroxidase (HRP)-conjugated secondary antibody for 1 h at room temperature. The bands on the membranes were visualized with the SuperLumia ECL HRP substrate kit (Abbkine, China). The acquired images were analyzed using Gel Pro3.0 (Media Cybernetics). Total protein level was normalized using the level of β-actin protein in each sample and presented as a percentage of the controls.

### Gene Expression Measurement Using RT-qPCR Technique

Total RNA of zebrafish larvaes and SH-SY5Y cells were extracted by using Trizol following the manufacturer’s protocols. The concentration and purity of RNA were measured by the NanoDrop 2000 spectrophotometer (Thermo, United States). RNA was reversely transcribed to single-strand cDNA by using the first-strand cDNA synthesis kit. Real-time quantitative polymerase chain reaction (RT-qPCR) analysis was performed on a CFX Connect fluorescence quantitative PCR instrument (BIO-RAD, United States) using the SYBR Green Quantitative kit. The reaction mixture (20 μl) contained 10 μl of 2×qPCR Mix, 0.6 μl of 10 μM each of the forward and reverse primers, 1 or 2 μl of template cDNA, and 7.8 or 6.8 μl of diethylpyrocarbonate (DEPC) H_2_O. The primer sequences are shown in Supplementary Table S1. PCR amplification was performed under the following conditions: 95°C for 15 min, followed by 39 cycles of 95°C for 10 s and 60°C for 30 s. The melt curve assay was conducted with the increasing temperature from 60 to 95°C by 0.3°C every 5 s. The relative mRNA expression levels were standardized with the β-actin and represented by a ratio of the expression in aconitine-treated groups versus that in the controls.

### Statistical Analysis

All statistical analyses were performed in SPSS 22.0 statistical software (IBM Corp). The normality of data were assessed using the Kolmogorov Smirnov test. The Levene’s test was used to evaluate the homogeneity of variance. The differences between groups were analyzed using the one-way analysis of variance (ANOVA) following the least-significant difference (LSD) or Tamhane’s T2 post-hoc comparison. All data were expressed as mean ± standard deviation (SD) of at least three independent experiments. The difference was considered to be significant at the *p* < 0.05 level.

## Results

### Aconitine Induced Morphological and Neurobehavioral Changes in Zebrafish Larvaes

**FIGURE 1:**
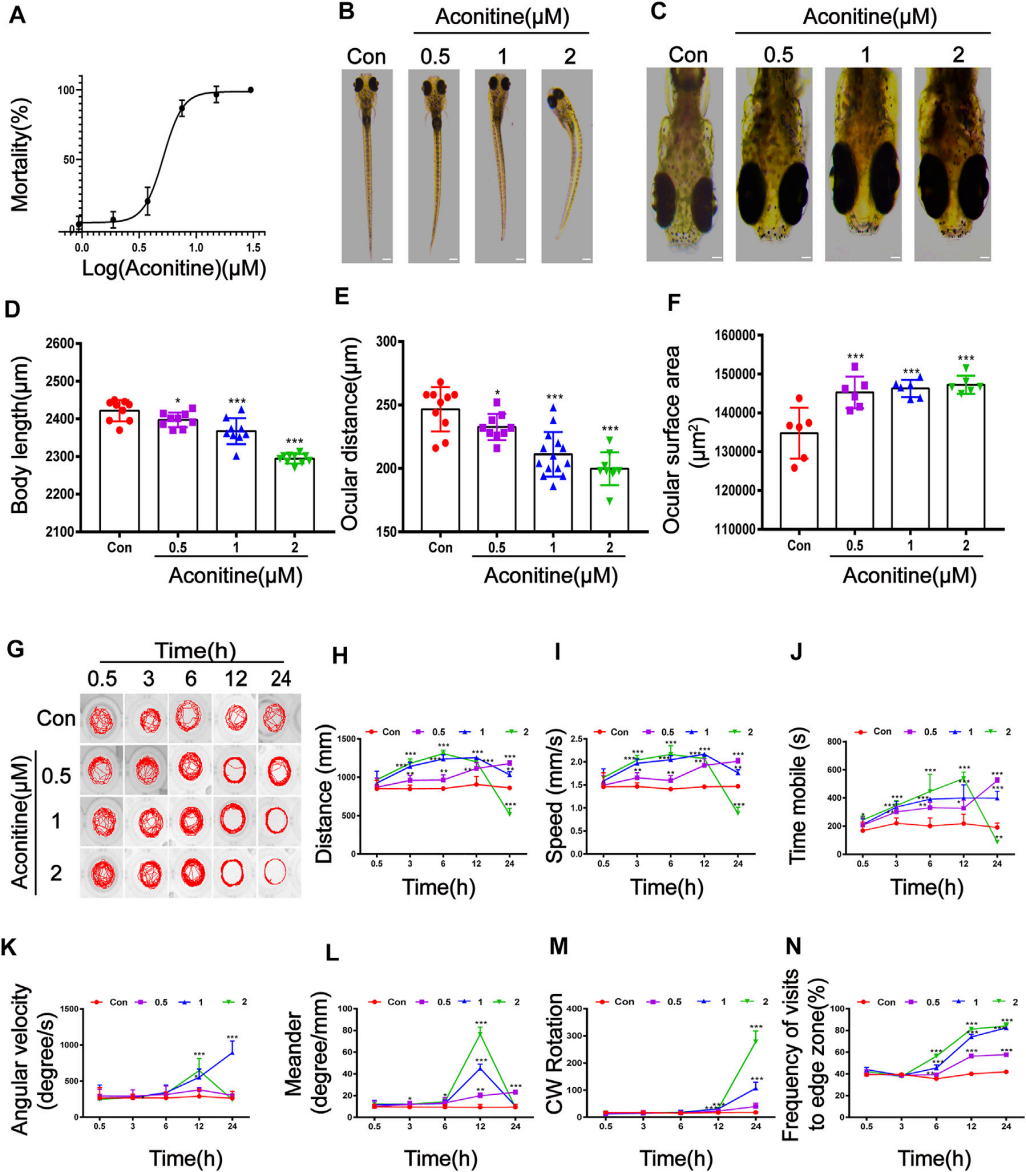
Morphological and swimming behavior changes of zebrafish larvaes after aconitine exposure. **(A)** Mortality of zebrafish larvaes exposed to aconitine at different concentrations (0.937–30 μM), *n* = 30. **(B–F)** Effects of exposure to aconitine on morphological parameters of zebrafish larvaes. Images were taken under a stereomicroscope (32× or 100×), *n* = 15. **(D)** Body length (μm). **(E)** Ocular distance (μm). **(F)** The surface area of eyes (μm^2^). **(G–N)** Effects of exposure to aconitine on neurobehavioral of zebrafish larvaes, *n* = 10. **(G)** Behavior trajectories. **(H)** Distance travelled (mm). **(I)** Movement speed (mm/s). **(J)** Time mobile (s). **(K)** Angular velocity (degree/s). **(L)** Meander (degree/mm). **(M)** CW rotation. **(N)** Frequency of visits to edge zone (%). The values are expressed as mean ± SD. *p < 0.05, **p < 0.01, ***p < 0.001 vs. control groups.

The results of acute toxicity test showed that zebrafish larvaes presented high mortality in an obvious concentration-dependent manner after treatment with aconitine (Figure 1A). Compared with the control group, the aconitine groups at doses of 0.937, 1.875, and 3.75 μM showed mortality percentages of 3.33%, 6.67%, and 20%, respectively. With the increasing doses of aconitine, the mortality rates gradually increased to 100%. The calculated LC50 of aconitine on larvaes for 24 h was 5.156 μM with 95% CI: 4.611–5.747 μM. The teratogenic effects of aconitine on the morphology of larvaes were investigated 24 h after administration. As a result, lighter pigmentation, body curvature, shorter body length and ocular distance, and increased surface area of the eyes in the aconitine groups were observed, compared with those in the control groups (Figures 1B–F). The locomotion and orientation of larvaes was examined at five different periods (0.5, 3, 6, 12, and 24 h) to reflect the behavior pattern of larvaes. Treatment with aconitine significantly increased the distance travelled and movement speed of larvaes after 3 h. The swimming distance and speed in 1 and 2 μM of aconitine groups reached the peak value at 12 and 6 h, respectively, following a decreasing trend. The above parameters in the 2 μM group significantly lowered, but the levels in the 1 μM group were still higher than those in the control group after 24 h (Figure 1H,I). The effect of aconitine treatment on time mobile was similar to the distance travelled and movement speed (Figure 1J). But the swimming time of the 2 μM group was fastest at 12 h and then dropped to the level below the control group. The angular velocity of the 2 μM group reached the maximum at 12 h, whereas this parameter continuously increased in the 1 μM group (Figure 1K). The meander of 1 and 2 μM groups reached the peak at 12 h and then decreased to the level of the control group, while the meander of 0.5 μM group increased in a time-dependent manner (Figure 1L). The CW rotation of animals exposed to 1 and 2 μM of aconitine for 12 h significantly increased compared with that of the control (Figure 1M). Moreover, the frequency of visits to the edge zone dose and time-dependently increased after treatment with aconitine for 6 h (Figure 1G,N). The larvaes in 1 and 2 μM of aconitine groups gradually showed unbalance swimming, upside down of head, tail violent tremor, intermittent *in situ* “circular” violent rotation, and other abnormal behaviors with the increasing exposure time, compared with those in the control groups. Given these, aconitine exposure resulted in dramatic alterations of the spontaneous movement and motor coordination in zebrafish larvaes.

### Aconitine Led to Cell Injury of Zebrafish Larvaes and SH-SY5Y Cells

**FIGURE 2:**
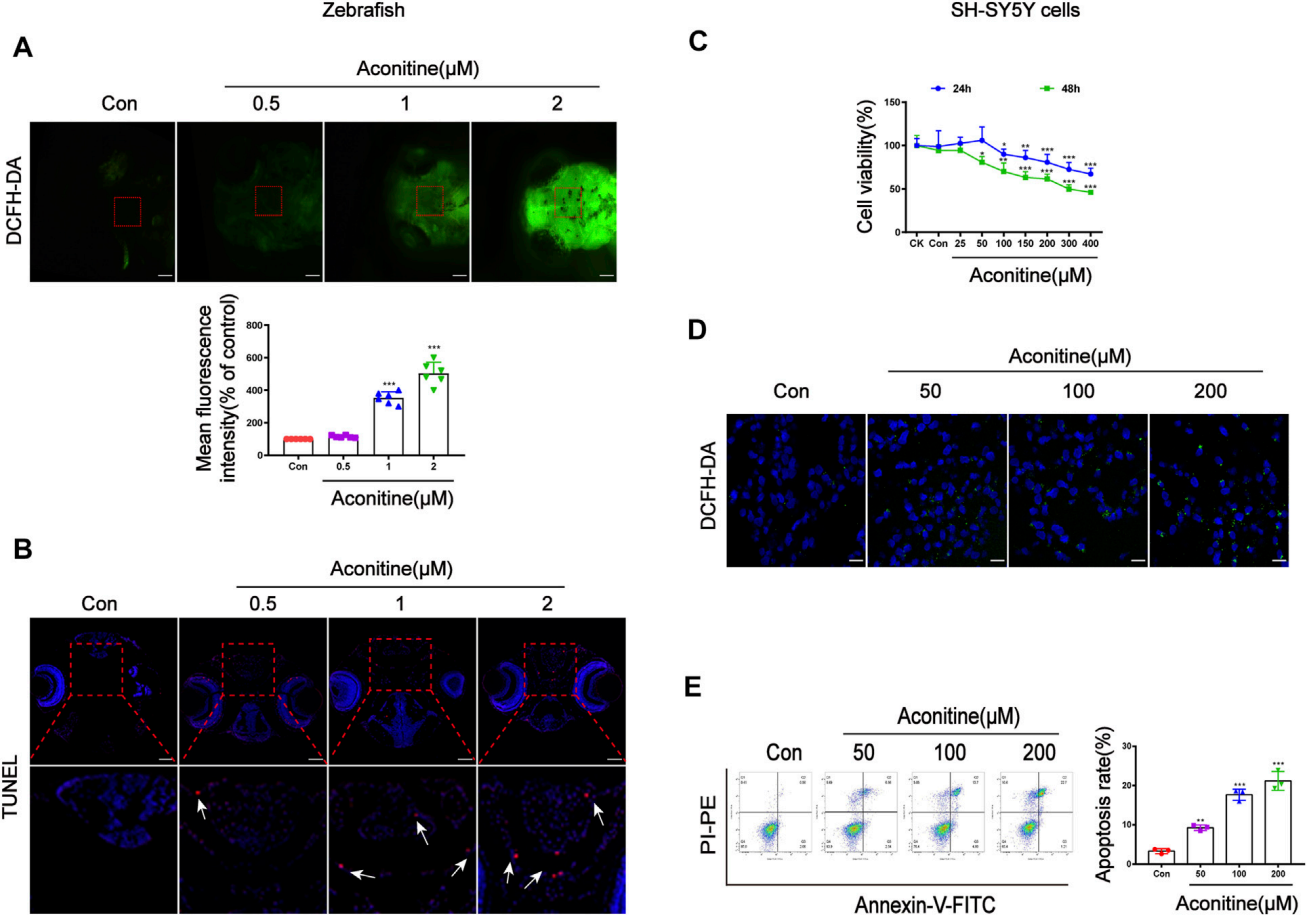
Cell damage of zebrafish larvaes and SH-SY5Y cells caused by aconitine. **(A)** The images of aconitine-treated larvaes by DCFH-DA (100×), *n* = 6. **(B)** The images of aconitine-exposed larvaes by TUNEL assay (100×), *n* = 3. **(C)** Cell viability of SH-SY5Y cells after 24 h or 48 h aconitine exposure, *n* = 8. **(D)** The images of aconitine-treated SH-SY5Y cells by DCFH-DA (200×), *n* = 3. **(E)** SH-SY5Y cell apoptosis aconitine-induced based on Annexin V/PI double staining by flow cytometry, *n* = 3. All photographs were taken under a stereoscopic fluorescence microscope or confocal microscope. The values are expressed as mean ± SD. *p < 0.05, **p < 0.01, ***p < 0.001 vs. control groups.

DCFH-DA staining was applied to determine intracellular ROS levels of zebrafish larvaes. Aconitine incubation at doses of 1 and 2 μM for 24 h significantly increased ROS production in the brain of zebrafish larvaes, compared with that in the control group (Figure 2A). TUNEL experiments were performed to observe whether aconitine induced cell apoptosis in the brain of zebrafish larvaes. The red fluorescence density of 2 μM aconitine greatly enhanced in the enlarged images compared to the control group, suggesting that aconitine could induce apoptosis (Figure 2B). *In vitro*, the cytotoxicity of aconitine on SH-SY5Y cells was assessed by the CCK-8 assay. Cell viability were significantly reduced in a dose and time-dependent manner after aconitine incubation (Figure 2C). After incubation with DCFH-DA probes, the green fluorescence in the 200 of μM aconitine exposure group also strengthened in cells (Figure 2D). Results from flow cytometry analysis demonstrated that aconitine dose-dependently induced apoptosis of SH-SY5Y cells (Figure 2E). Hence, aconitine could trigger ROS overproduction and cell apoptosis, leading to neurotoxicity in zebrafish larvaes and SH-SY5Y cells.

### Aconitine Disturbed Dopamine Homeostasis in Zebrafish Larvaes and SH-SY5Y Cells

**FIGURE 3:**
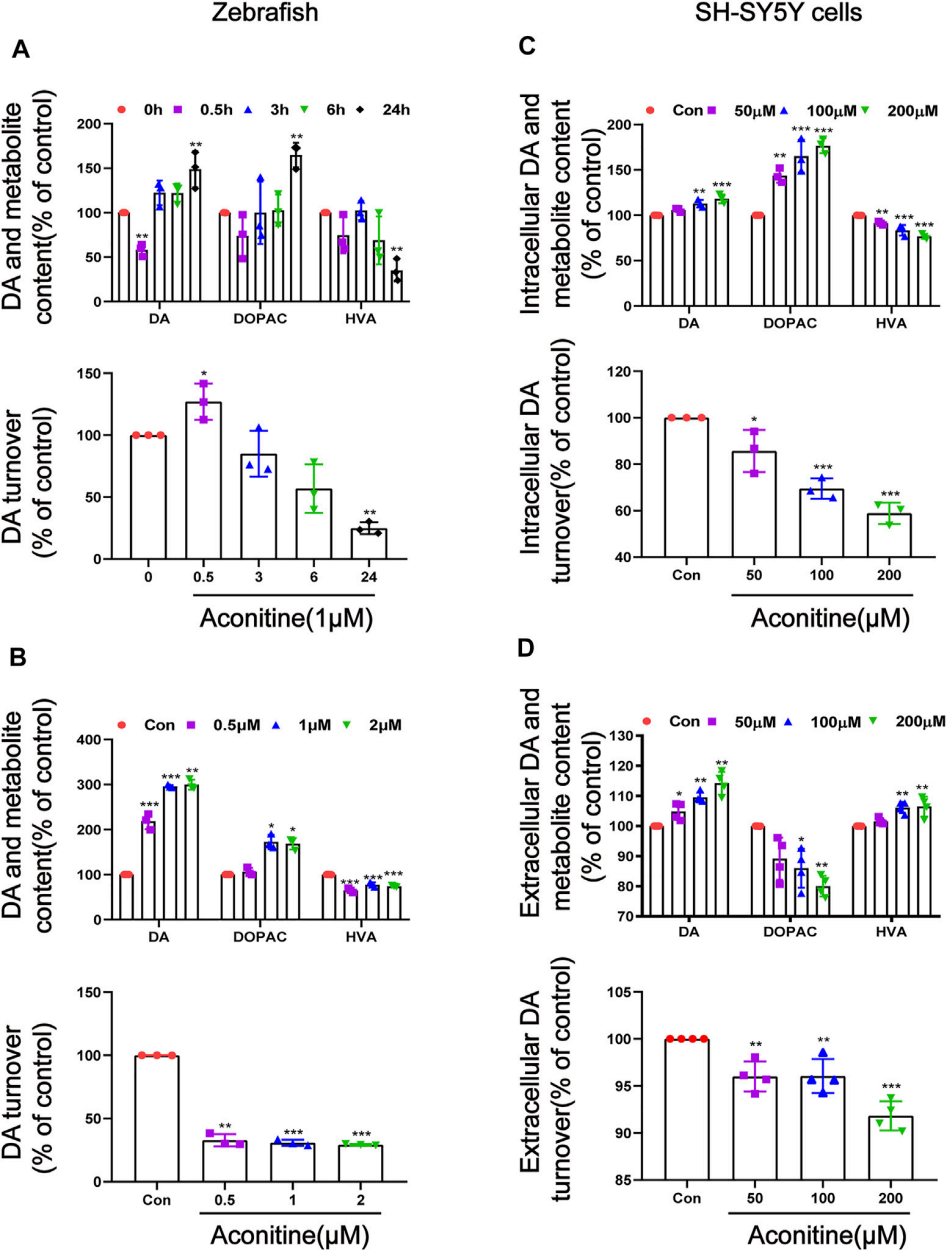
Alteration of dopamine and its metabolites levels in zebrafish larvaes and SH-SY5Y cells after aconitine exposure. **(A)** Dopamine, DOPAC and HVA levels and the turnover of dopamine after larvaes were treated with 1 μM aconitine for 0.5, 3, 6, and 24 h, respectively. **(B)** Dopamine, DOPAC and HVA contents and the turnover of dopamine after larvaes were incubated with aconitine at doses of 0.5, 1 and 2 μM for 24 h. **(C)** Intracellular dopamine, DOPAC and HVA levels and the turnover of dopamine at doses of 50, 100, and 200 μM for 24 h in SH-SY5Y cells. **(D)** Extracellular dopamine, DOPAC and HVA levels and the turnover of dopamine at doses of 50, 100, and 200 μM for 24 h in SH-SY5Y cells. The values are expressed as mean ± SD, *n* = 3. *p < 0.05, **p < 0.01, ***p < 0.001 vs. control groups.

To explore the relationship between aconitine-induced behavioral changes and dopamine homeostasis, we evaluated the turnover of dopamine *in vivo* and *in vitro*. First of all, dopamine, DOPAC, and HVA contents in zebrafish were detected at different time points after exposure to 1 μM of aconitine. As a result, dopamine level initially decreased at 0.5 h of aconitine treatment and the dopamine turnover that reflects dopamine metabolic rate significantly increased in zebrafish larvaes. However, aconitine induced a significant increase of dopamine and DOPAC levels at 24 h compared to the control, whereas HVA level significantly reduced, indicating that dopamine turnover went down (Figure 3A). In addition, zebrafish larvaes at six dpf were treated with different concentrations of aconitine for 24 h to explore the dose-response relationship between aconitine and dopamine turnover. Dopamine levels significantly increased in aconitine treatment groups compared with that in the control group, while DOPAC level only increased in the 1 and 2 μM of aconitine groups and HVA level decreased significantly after aconitine treatment (Figure 3B). The results also revealed that aconitine decreased significantly dopamine turnover in a dose-dependent manner. *In vitro*, Figure 3C,D, the iterative experiments revealed the significant effects of aconitine on intracellular and extracellular levels of dopamine, DOPAC, and HVA. Both intracellular and extracellular dopamine contents dose-dependently increased after aconitine incubation. As for the metabolites of dopamine, intracellular DOPAC level also increased in a dose-dependent manner, whereas intracellular HVA level significantly decreased. Extracellular DOPAC and HVA alterations were exactly the opposite of intracellular changes. Nevertheless, both intracellular and extracellular dopamine turnover dose-dependently slowed down. Thus, aconitine could affect dopamine turnover to perturb dopamine homeostasis *in vivo* and *in vitro*.

### Aconitine Affected Dopamine Metabolism-Related Function in Zebrafish Larvaes and SH-SY5Y Cells

**FIGURE 4:**
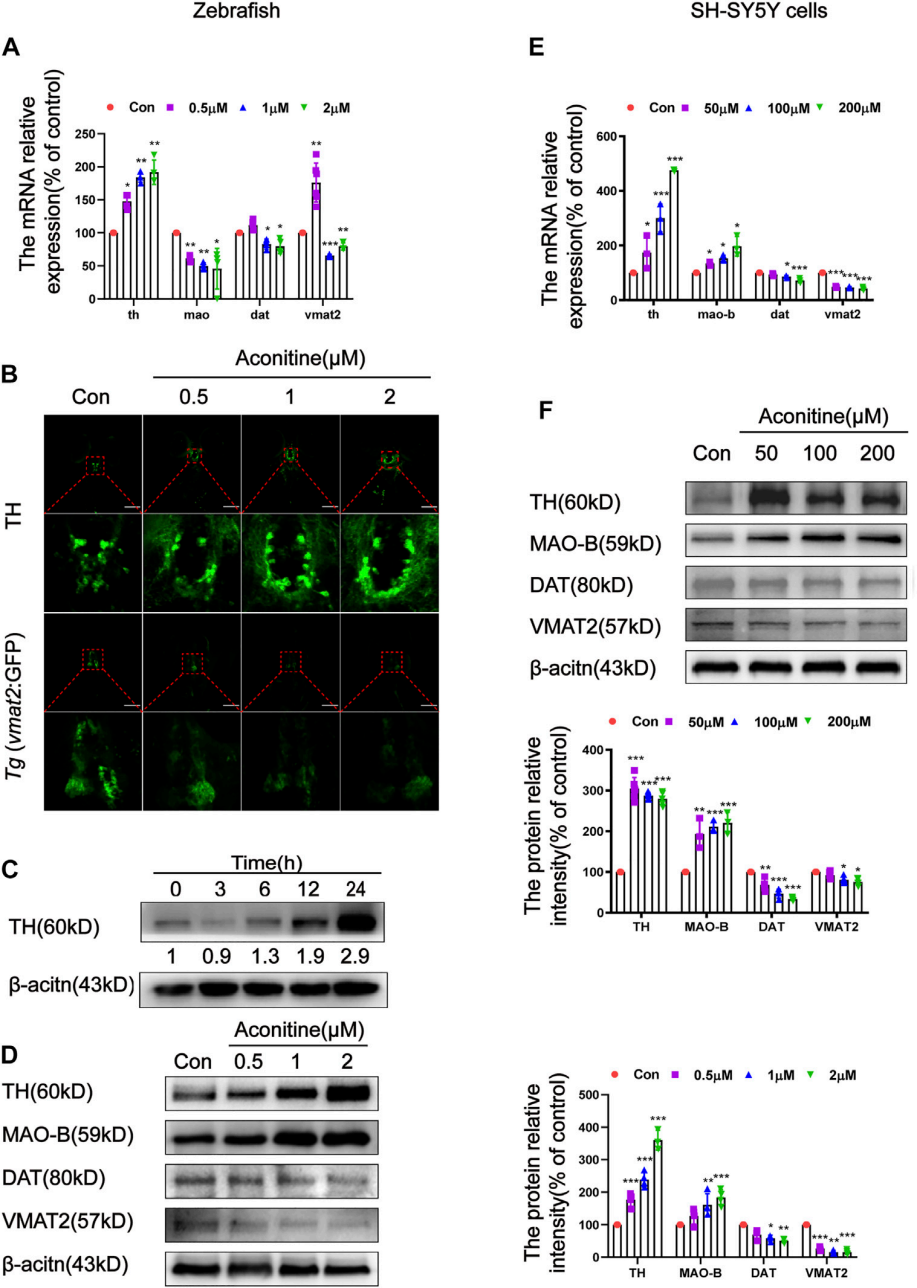
Effects of aconitine on TH, MAO, DAT and VMAT2 expressions in zebrafish larvaes and SH-SY5Y cells. **(A)** The mRNA expressions of *th*, *mao*, *dat*, *vmat2* with aconitine exposure in zebrafish larvaes, *n* = 6. **(B)** TH and VMAT2 positive cells were investigated using immunostaining and *Tg (vmat2:GFP)* transgenic zebrafish larvaes, respectively, *n* = 5. **(C)** Effect of different exposure times of 1 μM aconitine on the protein expression of TH, *n* = 4. **(D)** The protein levels of TH, MAO-B, DAT and VMAT2 with aconitine exposure in zebrafish larvaes, *n* = 4. **(E)** The mRNA expressions of *th*, *mao-b*, *dat*, *vmat2* with aconitine exposure in SH-SY5Y cells, *n* = 3. **(F)** The protein levels of TH, MAO-B, DAT, and VMAT2 with aconitine exposure in SH-SY5Y cells, *n* = 7. All photographs in which the green fluorescence represented the immune-reactive cells were taken under a confocal microscope (100×). The values are expressed as mean ± SD. *p < 0.05, **p < 0.01, ***p < 0.001 vs. control groups.

As mentioned above, aconitine disrupted dopamine homeostasis by decreasing the dopamine turnover both *in vivo* and *in vitro*. The dopamine metabolism-related function, including dopamine synthesis, degradation, transport, and reuptake, were examined to further explore the underlying mechanisms. As a rate-limiting enzyme of dopamine synthesis, TH protein expression was measured after treatment with 1 μM of aconitine for different times. As shown in Figure 4A,D, the protein and mRNA levels of TH remarkably increased in a concentration-dependent manner after aconitine treatment for 24 h. TH protein expression also increased in a time-dependent manner, and the gray value at 24 h increased to 2.9 times of that in untreated condition (Figure 4C). The *mao* mRNA expression significantly decreased in all treatment groups. However, the MAO-B protein expression in 1 and 2 μM of aconitine groups significantly increased, compared with that in the control group. The mRNA and protein expression levels of DAT significantly decreased in 1 and 2 μM of aconitine groups. The *vmat2* mRNA expression in the 0.5 μM group had a sharp rise and then and decreased significantly in 1 and 2 μM groups, compared with that in the control group. The VMAT2 protein levels had a significant reduction in all treatment groups. Whole-mount immunofluorescence staining and *Tg (vmat2:GFP)* transgenic zebrafish were used to evaluate the expression of TH and VMAT2 in dopaminergic neurons of zebrafish larvaes, respectively (Figure 4B). Similar to the results of protein expression, the number of TH-positive neurons of the telencephalon greatly increased, while the fluorescence intensity of mesencephalon decreased in *Tg (vmat2:GFP)* transgenic zebrafish after different concentrations of aconitine exposure (Figure 4D). In SH-SY5Y cells (Figure 4E,F), the TH mRNA and protein expressions significantly increased in the aconitine treatment groups compared with that in the control group. In addition, the mRNA and protein levels of MAO-B significantly increased in a concentration-dependent manner, whereas both DAT and VMAT2 mRNA and protein expression levels significantly decreased after aconitine treatment.

### Aconitine Activated the AC/cAMP/PKA Pathway by Up-Regulating D1R and Down-Regulating D2R in Zebrafish Larvaes and SH-SY5Y Cells

**FIGURE 5:**
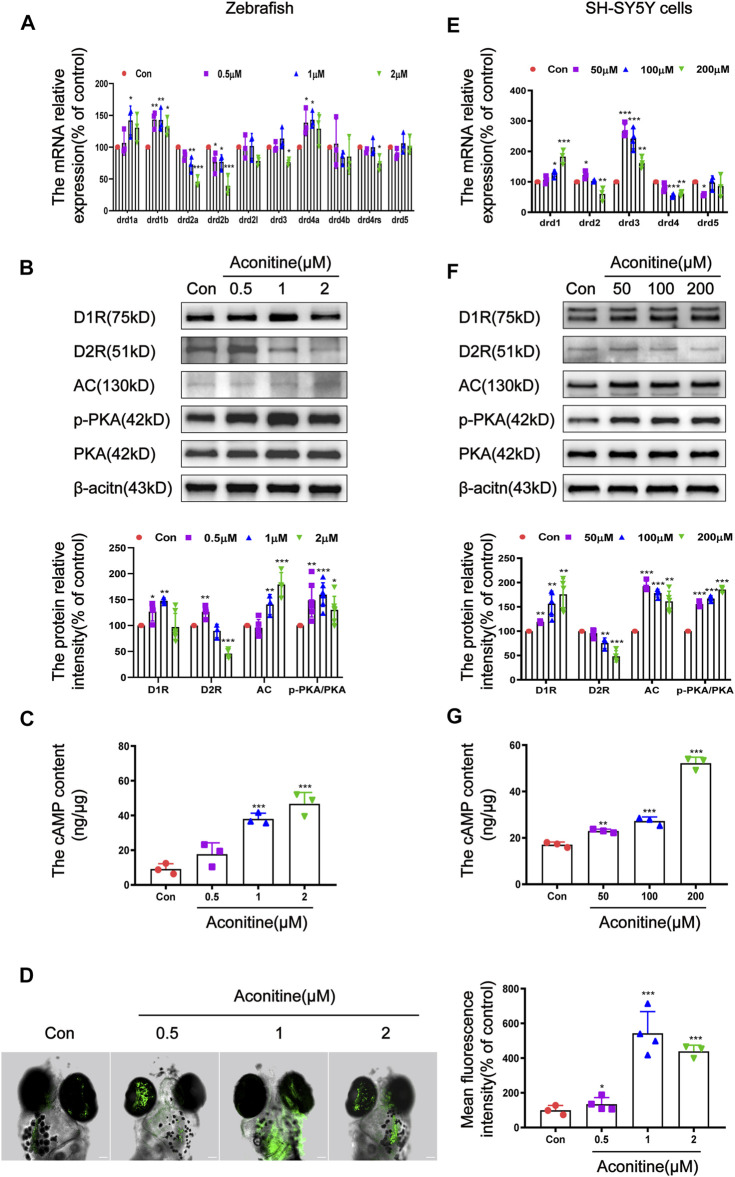
Regulation of DR-mediated AC/cAMP/PKA signalling pathway by aconitine in zebrafish larvaes and SH-SY5Y cells. **(A)** Expression of DR mRNA with aconitine exposure in zebrafish larvaes, *n* = 3. **(B)** D1R, D2R, AC, p-PKA, and PKA protein expressions with aconitine exposure in zebrafish larvaes, *n* = 7. **(C)** The cAMP contents were examined with aconitine exposure by ELISA assays in zebrafish larvaes, *n* = 3. **(D)** Changes of intracellular Ca^2+^ after treatment with aconitine in zebrafish larvaes, *n* = 5. **(E)** Expression of DR mRNA with aconitine exposure in SH-SY5Y cells, *n* = 5. **(F)** D1R, D2R, AC, p-PKA and PKA protein expressions with aconitine exposure in SH-SY5Y cells, *n* = 5. **(G)** The cAMP contents were examined with aconitine exposure by ELISA assays in SH-SY5Y cells, *n* = 3. All photographs were taken under a confocal microscope (100×). The values are expressed as mean ± SD. *p < 0.05, **p < 0.01, ***p < 0.001 vs. control groups.

It is well known that DRs can regulate the content of cAMP to mediate the downstream signal transduction (Beaulieu and Gainetdinov, 2011). Therefore, we further investigated the effects of aconitine on the DRs-mediated AC/cAMP/PKA signalling pathway. The mRNA expression of D_1_-like dopamine receptors (*drd1a*, *drd1b* and *drd5a*) and D_2_-like dopamine receptors (*drd2a*, *drd2b*, *drd2l*, *drd3*, *drd4a*, *drd4b*, and *drd4-rs* subtypes) was assessed to clarify the states of dopamine receptors after aconitine exposure in zebrafish (Yamamoto et al., 2015; Shontz et al., 2018). As depicted in Figure 5A, the *drd1a* mRNA levels remarkably rose only in 1 μM treatment group, compared with that in the control group, while the *drd1b* mRNA levels significantly increased in all aconitine-treated groups. The mRNA expression levels of *drd2a* and *drd2b* were significantly attenuated by aconitine in a concentration-dependent manner. The transcript levels of *drd3* and *drd4-rs* significantly reduced only in the 2 μM group compared with that in the control group. By contrast, 0.5 and 1 μM of aconitine significantly increased the *drd4a* levels. Results in Figure 5B showed that the D1R protein levels significantly increased in 0.5 and 1 μM groups. Interestingly, aconitine at different concentrations has a bidirectional regulation effect on the D2R protein expression. Results indicated that the expression of D2R protein significantly increased at a low concentration (0.5 μM) and reduced at a high concentration (2 μM) in zebrafish larvaes. The AC protein levels were dose-dependently increased by aconitine treatment. Aconitine also significantly enhanced the phosphorylation levels of PKA in all aconitine groups. The content of cAMP in larvaes was detected by ELISA. As a result, the levels of cAMP significantly increased in 1 and 2 μM groups (Figure 5C). As shown in Figures 5D, 1 μM of aconitine significantly increased the Ca^2+^ fluorescence intensity in the brain of larvaes after 24 h of exposure.

Differ from zebrafish, the mammals have five main subtypes of dopamine receptors, which are encoded by the genes *drd1*, *drd2*, *drd3*, *drd4*, and *drd5* (Beaulieu et al., 2015). The effects of aconitine on the expression of these genes were evaluated in SH-SY5Y cells. As shown in Figure 5E, the *drd1* mRNA expression significantly increased in a concentration-dependent manner, whereas the *drd4* expression significantly decreased, compared to the controls. The *drd2* mRNA levels significantly increased at 50 μM and significantly decreased at 200 μM. The mRNA expression of *drd3* increased significantly after different concentrations of aconitine treatment. The *drd5* mRNA expression significantly decreased at 50 μM. *In vitro*, the effects of aconitine on the AC/cAMP/PKA signalling pathway was similar to that of zebrafish larvaes (Figure 5F,G). After aconitine exposure, the protein levels of D1R, AC, and cAMP content were significantly enhanced, while the ratio of p-PKA to PKA was significantly increased, indicating that D1R-mediated AC/cAMP/PKA signalling pathway was activated. Meanwhile, aconitine significantly reduced the protein expression of the D2R in 100 and 200 μM groups in cells, which differ from the vivo results. In general, *in vivo* and *in vitro* studies showed that aconitine activated the AC/cAMP/PKA signalling pathway by regulating the expression of D1R and D2R.

### DRs Intervention Inhibited the Excitatory Behaviors Induced by Aconitine in Zebrafish Larvaes

**FIGURE 6:**
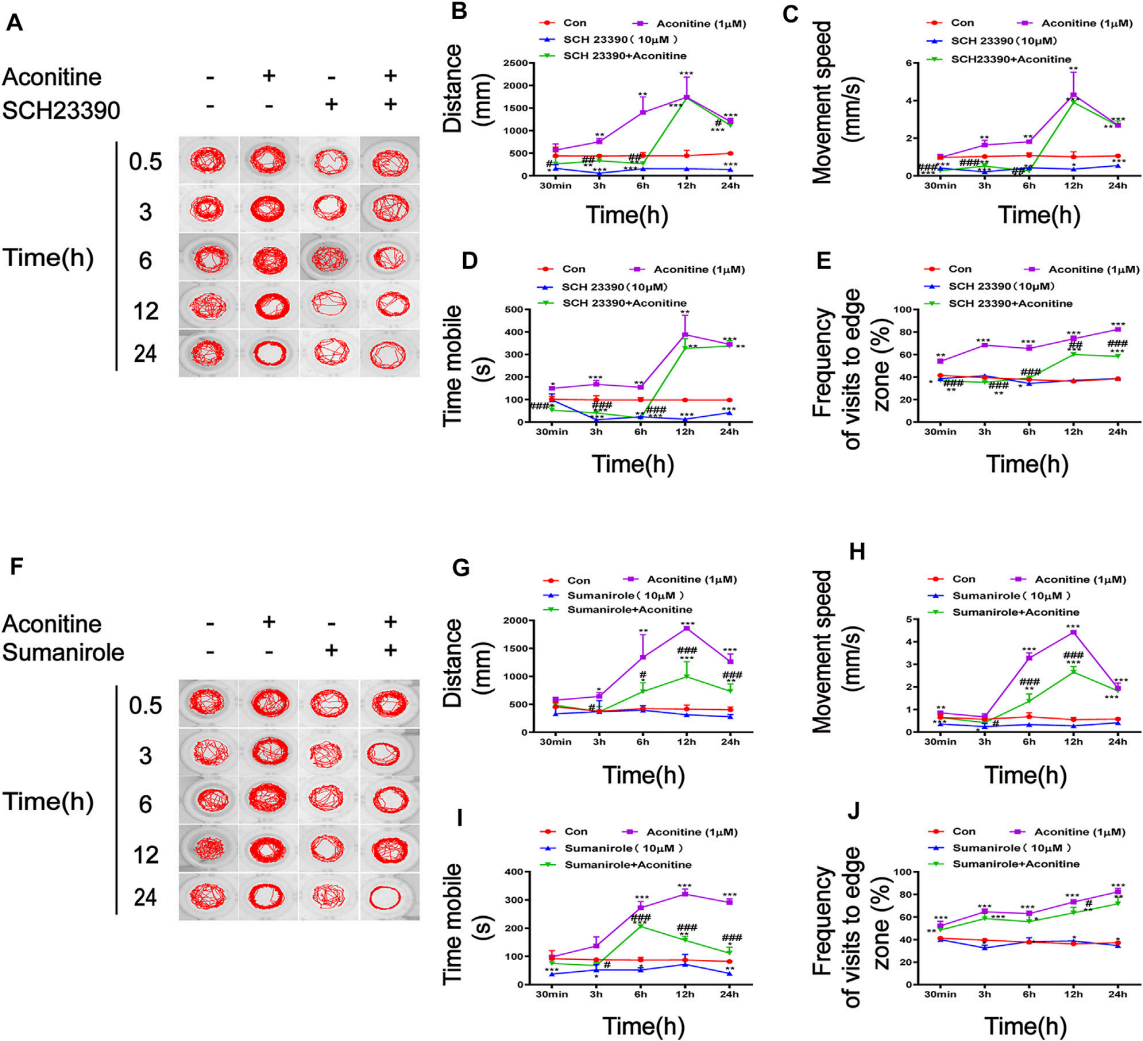
Effects of SCH23390 and sumanirole on behavioral changes induced by aconitine in zebrafish larvaes. **(A–E)** SCH23390 inhibited aconitine-stimulated excitatory behaviors, *n* = 10. **(F–J)** Sumanirole suppressed aconitine-induced excitatory behaviors, *n* = 10. The values are expressed as mean ± SD. *p < 0.05, **p < 0.01, ***p < 0.001 vs. control groups. ^#^ *p* < 0.05, ^##^ *p* < 0.01, ^###^ *p* < 0.001 vs. aconitine-treated groups.

The effects of SCH23390 (a selective inhibitor of D1R) and sumanirole (a highly selective D2R complete agonist) on the swimming behaviors affected by aconitine were evaluated. Based on the results of the locomotor behavioral test, we investigated the concentration and time of SCH23390 and sumanirole incubation prior to aconitine treatment (Supplementary Figure S1A, B). The larvaes were treated with 1 μM of aconitine for 24 h after the presence or absence of 10 μM SCH23390 or 10 μM sumanirole for 2 h. As shown in Figure 6A–D, the increases of distance, movement speed, and mobility time in response to aconitine stimulation were significantly inhibited at 0.5, 3, and 6 h after SCH23390 pretreatment. SCH23390 significantly attenuated the increase of frequency of visits to the edge zone induced by aconitine at 0.5-24 h (Figure 6E). Furthermore, aconitine-induced increases in the distance and mobility time were also effectively inhibited at 3, 6, and 12 h after sumanirole administration, while the movement speed was significantly lowered by sumanirole at 6 and 12 h (Figures 6F–I). In contrast to SCH23390, pretreatment with sumanirole barely reversed the increases of frequency of visits to the edge zone by aconitine-induced (Figure 6J).

### DRs and PKA Intervention Reversed the Increase of intracellular Ca^2+^ Induced by Aconitine in Zebrafish Larvaes

**FIGURE 7:**
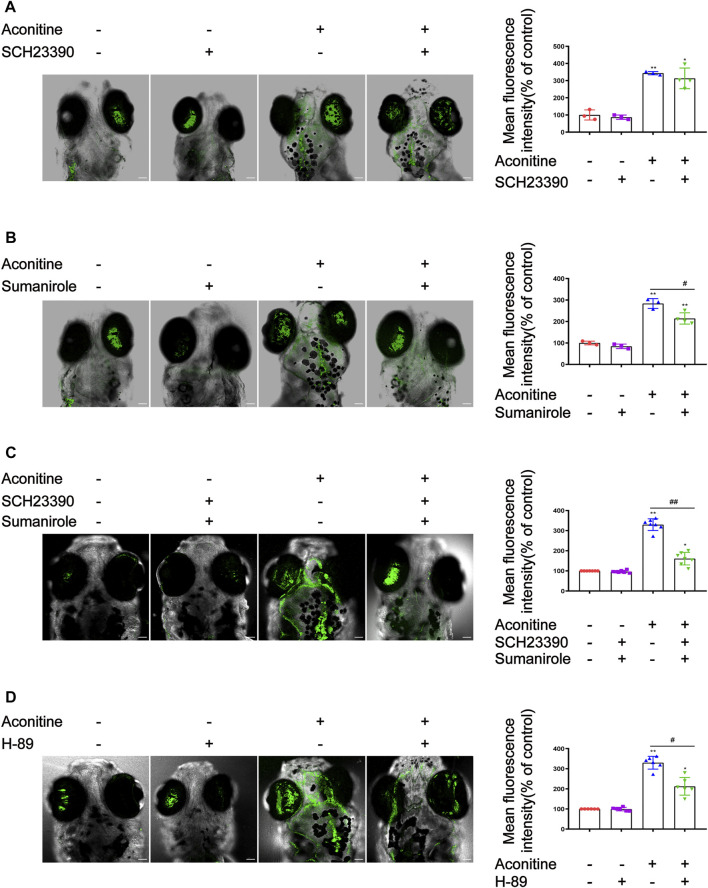
Effects of SCH23390, sumanirole and H-89 on intracellular Ca^2+^ changes induced by aconitine in zebrafish larvaes. **(A)** Effects of SCH23390 on intracellular Ca^2+^ changes induced by aconitine. **(B)** Sumanirole suppressed aconitine-induced the increase of intracellular Ca^2+^. **(C)** Pretreatment with SCH23390 and sumanirole inhibited the rise of intracellular Ca^2+^ aconitine-stimulated. **(D)** H-89 inhibited the increase of intracellular Ca^2+^ induced by aconitine. All photographs were taken under a confocal microscope (100×). The values are expressed as mean ± SD, *n* = 8. *p < 0.05, **p < 0.01, ***p < 0.001 vs. control groups. ^#^ *p* < 0.05, ^##^ *p* < 0.01, ^###^ *p* < 0.001 vs. aconitine-treated groups.

The effects of SCH23390, sumanirole, and H89 (a selective inhibitor of PKA) on intracellular Ca^2+^ induced by aconitine treatment were determined. As shown in Figure 7A, although the green fluorescence of the aconitine group with the presence of SCH23390 was slightly weakened compared with that of the aconitine group, the difference had no significance. Fortunately, pretreatment with sumanirole effectively inhibited the increase of intracellular Ca^2+^ induced by aconitine in the brain of larvaes (Figure 7B). Co-pretreatment with SCH23390 and sumanirole further enhanced the inhibition of Ca^2+^ elevation in response to aconitine stimulation (Figure 7C). As another target molecule of the AC/cAMP/PKA signalling pathway, the effects of PKA intervention with H-89 on intracellular Ca^2+^ in the brain of larvaes were examined. Similar to DRs modulators, the pretreatment concentration and time of H-89 were also optimized (Supplementary Figure S1C). The larvaes were treated with 10 μM of H89 for 3 h before aconitine administration. The result showed that H-89 effectively reversed aconitine-induced an increase of intracellular Ca^2+^ zebrafish larvaes (Figure 7D).

### DRs Intervention Attenuated Aconitine-induced Cell Injury by Inhibiting the AC/cAMP/PKA Pathway in SH-SY5Y Cells

**FIGURE 8:**
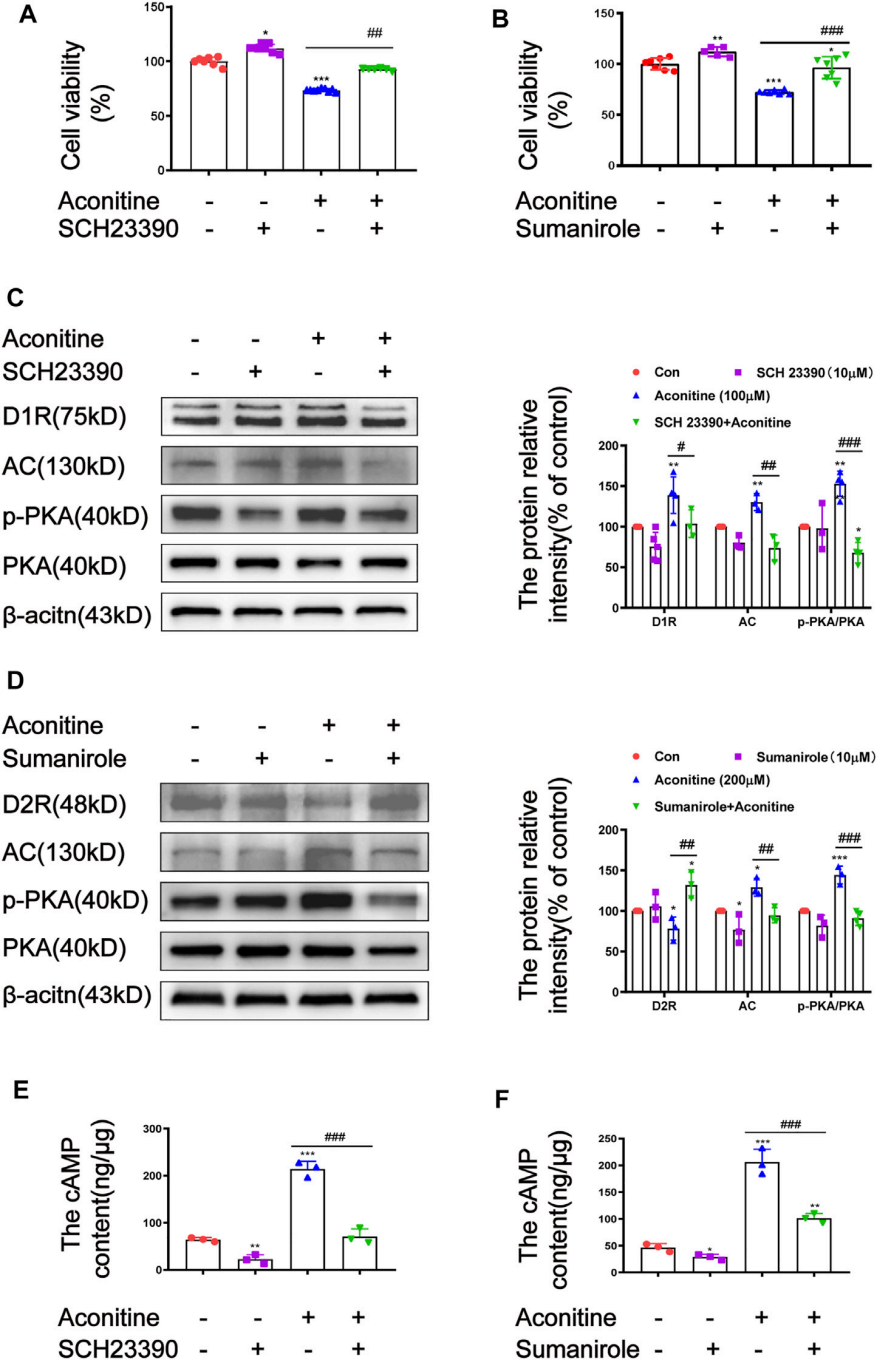
Effects of SCH23390 and sumanirole on aconitine-induced the activation of AC/cAMP/PKA pathway in SH-SY5Y cells. **(A–B)** SCH23390 and sumanirole inhibited aconitine-induced cytotoxicity in SH-SY5Y cells, respectivevly, *n* = 9. **(C–F)** SCH23390 and sumanirole suppressed aconitine-mediated the activation of AC/cAMP/PKA pathway, *n* = 5. The values are expressed as mean ± SD. *p < 0.05, **p < 0.01, ***p < 0.001 vs. control groups. ^#^ *p* < 0.05, ^##^ *p* < 0.01, ^###^ *p* < 0.001 vs. aconitine-treated groups.

DRs modulators SCH23390 and sumanirole were separately used to further elucidate the roles of D1R and D2R in aconitine-activated AC/cAMP/PKA pathway. Similar to *in vivo* experiments, the pretreatment concentration and time of DRs modulators in cells were also investigated (Supplementary Figure S2). SH-SY5Y cells were pre-incubated with 10 μM of SCH23390 for 2 h or 10 μM of sumanirole for 0.5 h before aconitine treatment, respectively. Aconitine-induced cytotoxicity of SH-SY5Y cells could be effectively attenuated by 10 μM of SCH23390 or 10 μM of sumanirole (Figure 8A, B). In addition, both pretreatment with SCH23390 and sumanirole inhibited the activation of the AC/cAMP/PKA pathway caused by aconitine (Figures 8C–F). These results indicated that aconitine-induced neurotoxicity in SH-SY5Y cells might partially attribute to the activation of the AC/cAMP/PKA/pathway by up-regulating D1R or down-regulating D2R.

## Discussion

*Aconitum* species are one kind of Chinese medicines commonly used in clinic. However, the unreasonable use of these plants poses high risks of cardiotoxicity and neurotoxicity. Previous researches demonstrated that DDAs were the main bioactive and toxic components of *Aconitum* species (Han Bo et al., 2017). Aconitine, an important DDA extracted from *Aconitum*, is responsible for the toxic effects of these species. Progressive dyskinesia and involuntary tremor are the typical symptoms of aconitine intoxication, which may be related to the regulation of dopamine (Zhou et al., 2019). However, the mechanism underlying aconitine-induced neurological impairment remains unclear. In this study, zebrafish larvaes and SH-SY5Y cells were used as models to investigate the mechanisms of aconitine-induced neurological damage and the key signalling pathway involved.

Aconitine treatment increased the mortality rate of zebrafish larvaes, induced deformities, inhibited the proliferation of SH-SY5Y cells, and led to ROS overproduction and apoptosis both *in vivo* and *in vitro*. These results indicated that aconitine had obvious neurotoxicity. In behavioral experiments, a high concentration of aconitine (2 μM) significantly reduced the spontaneous behaviors of larvaes after 24 h of exposure, consistent with the results of previous experiments (Xia et al., 2021). However, the larvaes treated with 2 μM of aconitine exhibited a significant increase in locomotor activity before experiencing a remarkable decrease, which may be attributed to different concentrations and exposure patterns. In addition, aconitine significantly increased the meander and angular velocity of zebrafish larvaes within 12 h. The CW rotation was enhanced in a concentration- and time-dependent manner. These changes of movement direction and posture parameters indicated that aconitine exposure might act as a trigger for motor incoordination in larvaes (Capriello et al., 2019; Cheng et al., 2020). Within 24 h after treatment with 2 μM of aconitine, the swimming activity of zebrafish increased sharply (first phase) followed by a rapid ‘swirling’ swimming behavior (second phase) and a series of paroxysmal convulsions. The swimming behaviors of larvaes finally were significantly reduced by aconitine. These results suggested that aconitine might induce epileptic-like behavior in zebrafish larvaes. Previous studies proved that aconitine could trigger epileptic-like activity in the neocortex and hippocampus of rats and produce acute and long-term excitatory effects on cerebral cortex activity (Ameri et al., 1996; Voss et al., 2008). Interestingly, the larvaes exposed to aconitine developed phenotypes associated with anxiety-like behavior, such as increased locomotor activity, more marginal thixotropy, and irregular and repetitive behaviors with the increasing doses and treatment time. Thus, these results verified that aconitine exposure could trigger neurological impairment *in vivo* and *in vitro*.

The dopaminergic nervous system plays an important role in the regulation of epilepsy and anxiety-like behavior (Mathew et al., 2001; Werhahn et al., 2006; de la Mora et al., 2010; Costa et al., 2016; Peng et al., 2021; Yaksi et al., 2021). Previous studies indicated that aconitine triggered the release of dopamine, following oxidative stress and dysfunction of dopaminergic nervous system (Zhao et al., 2010). Given this, we speculated that dopaminergic system dysfunction might be the possible mechanism underlying aconitine-induced neurotoxicity. To verify this hypothesis, the effects of aconitine on the levels of dopamine and its metabolites in zebrafish larvaes and SH-SY5Y cells were firstly investigated. The results revealed that aconitine treatment promoted the accumulation of intracellular and extracellular dopamine to result in an imbalance of dopamine homeostasis. As is well-known, the biosynthesis of dopamine derived from tyrosine is controlled by TH (Klein et al., 2019). Excessive dopamine can promote MAO and COMT-mediated catabolism, thereby increasing the levels of DOPAC and HVA. Dopamine is stored in synaptic vesicles after uptake by the VMAT2 (Caudle et al., 2008). Dopamine stored in vesicles is released by exocytosis to the synaptic cleft (Meiser et al., 2013). Dopamine action is terminated by reuptake from the synaptic cleft via DAT (Liu et al., 2020). Therefore, some functional proteins involved in dopamine synthesis, storage and transport, degradation, release, and reuptake play key roles in maintaining dopamine balance from the cytoplasm to the synaptic cleft. The results of immunocytochemistry revealed that the fluorescence intensity of TH significantly increased in the telencephalon of zebrafish larvaes treated with aconitine. TH mRNA and protein levels in zebrafish larvaes and SH-SY5Y cells were also markedly increased after aconitine exposure. These results suggested that aconitine promoted the synthesis of dopamine, which may be an important reason for the increase of dopamine levels after aconitine treatment. Furthermore, aconitine exposure dose-dependently reduced the fluorescence intensity in the GFP-labelled *vmat2* transgenic zebrafish. The mRNA and protein expression of VMAT2 in zebrafish larvaes and SH-SY5Y cells were also significantly decreased by aconitine treatment. Hence, aconitine treatment decreased the amount of dopamine transported to the synaptic vesicles via VMAT2, which contributed to the increase of intracellular dopamine level. Moreover, since the locally acidic environment of synaptic vesicles prevents dopamine autoxidation (Farzam et al., 2020), the reduction of VMAT2 may exacerbate dopamine oxidation. In addition, we also analyzed the effect of aconitine on dopamine transporter DAT. Aconitine treatment significantly decreased the expression of DAT mRNA and protein in zebrafish larvaes and SH-SY5Y cells, thereby decreasing the reuptake amount of dopamine from the synaptic cleft to the presynaptic nerve cells, which also resulted in the increase of dopamine level in cells. In our experiment, aconitine induced an increase in MAO expression and DOPAC content. Excessive dopamine in the cytoplasm can promote the stress-increase of MAO (Hermida-Ameijeiras et al., 2004). Thus, the increase of MAO expression may be a compensatory response to balance intracellular dopamine level. Interestingly, the accumulation of dopamine in the cytoplasm produces H_2_O_2_ under the action of self-non-enzymatic oxidation or MAO, which can trigger oxidative stress. What’s more, oxidative products-dopamine semiquinone (DASQ) and dopamine o-quinone (DAQ) can mediate protein modification and interfere with protein stability and cellular physiology, which contribute to the death of dopaminergic cells (Stokes et al., 1999; Hermida-Ameijeiras et al., 2004; Farzam et al., 2020). We also confirmed that aconitine could induce ROS overproduction and apoptosis in zebrafish larvaes and SH-SY5Y cells. Ugun-Klusek and others (Ugun-Klusek et al., 2019) revealed that mitochondrial ROS is mainly produced by the electron transport chain, but mitochondrial ROS can also be generated by the enzymatic action of several other mitochondrial enzymes including MAOs. Our previous studies revealed that aconitine could affect mitochondrial aerobic respiration and mitochondrial dynamics (Liang Yang et al., 2021). Therefore, neuronal damage caused by oxidative stress may contribute to the decrease of locomotor activity of the larvaes after 24 h of aconitine exposure. However, there are differences between the mRNA levels and protein levels of MAO in aconitine-exposed zebrafish larvaes. Zebrafish have only one type of MAO, being termed as the zMAO (Arslan and Edmondson, 2010), while humans have two MAO subtypes (MAO-A and MAO-B). Some studies indicated that the zMAO is functionally more similar to human MAO-A than MAO-B (Razali et al., 2021). However, the protein level of MAO in zebrafish larvaes was detected by the incubation with the human Monoamine oxidase B antibody in our experiments, thus magnifying the differences between the mRNA levels and protein levels of MAO in aconitine-exposed zebrafish larvaes. Similarly, aconitine also had great influence on the mRNA expression of genes related to dopamine synthesis, storage, degradation, and reuptake. These changes not only disrupted dopamine homeostasis to induce oxidative stress and apoptosis, but also increased dopamine levels from the synaptic cleft to the postsynaptic membrane receptors, which may alter DRs-mediated molecular events in the postsynaptic membrane.

Dopamine binds to five subtypes of receptors in mammals, whereas zebrafish has more DR subtypes due to gene replication events. In our study, aconitine treatment significantly increased the expression of *drd1* in zebrafish larvaes and SH-SY5Y cells, but decreased the expression of *drd2*. In addition, the mRNA expression of *drd3* was different between zebrafish larvaes and SH-SY5Y cells. The effects of aconitine on *drd4* mRNA expression varied on different subtypes. No significant difference in terms of the mRNA expression of *drd5* was found. Furthermore, D1R and D2R of the dopaminergic nervous system in zebrafish is largely conserved compared with that in other vertebrates, including homologous animals (Panula et al., 2010). D1R and D2R play vital roles in regulating animal behavior, emotion, and learning (Souza et al., 2011; Beaulieu et al., 2015; Kung et al., 2015; Tran et al., 2015; Costa et al., 2016; Peng et al., 2021). Hence, D1R- and D2R-mediated signalling pathways were first discussed in our study. Several studies have shown that D_1_-like receptors stimulated the release of intracellular Ca^2+^ via cAMP-dependent or cAMP-independent manner, whereas D_2_-like receptors play an opposite role in calcium regulation (Bergson et al., 2003; Beaulieu et al., 2015). As an important second messenger, Ca^2+^ is involved in a variety of physiological and pathological processes. Increasingly, it is recognized that epilepsy and anxiety may be related to various calcium signalling pathways, including the direct effects on membrane excitability through Ca^2+^ influx by ion channels and the indirect pathways through G-protein coupling (Steinlein, 2014; Rajakulendran and Hanna, 2016; Zamponi, 2016). As expected, results found that aconitine significantly potentiated PKA phosphorylation and increased intracellular Ca^2+^ concentration via D1R and D2R-mediated cAMP-dependent pathways. *In vivo* experiments, aconitine significantly increased D1R and decreased D2R expression, respectively, thus activating the AC/cAMP/PKA pathway to increase intracellular Ca^2+^ level. D1R is generally coupled to G_s/olf_ and stimulates the production of cAMP and PKA activity, whereas D2R is bound to G_i/o_ and suppresses the production of cAMP, thereby decreasing PKA phosphorylation level (Beaulieu and Gainetdinov, 2011). Interestingly, the activation of D1R and D2R by low-dose of aconitine did not significantly affect the levels of AC and cAMP, but increased the phosphorylation level of PKA and the concentration of intracellular Ca^2+^, indicating that D1R and D2R may regulate the phosphorylation of PKA by cAMP-independent pathway. For instance, D1R and D2R may be involved in G_αq/11_-mediated dopaminergic signalling, which may reduce the phosphorylation of DARPP-32 through CDK5 and enhance PKA-mediated signalling (Beaulieu et al., 2015). Furthermore, the co-activation of D1R and D2R promoted the formation of D1R-D2R heterodimers, and the activation of D1R-D2R complex induced the recruitment of G_αq/11_, which mediated dopaminergic signalling (Beaulieu et al., 2015). In addition, D2R bound to G_βγ_ may also regulate PLC/PKC/CDK5/DARPP-32 pathway to activate PKA (Beaulieu et al., 2015). It was worth noting that D1R was not activated by higher dose aconitine treatment, which may be related to D1R internalization (Ng et al., 1995). Studies have shown that PKA phosphorylation could promote DRs endocytosis (Kohout and Lefkowitz, 2003; Cho et al., 2010). *In vitro* experiments, D1R was stimulated after aconitine exposure, whereas D2R was suppressed, resulting in the activation of the AC/cAMP/PKA pathway via the coordination of D1R and D2R. Low dose aconitine induced the increase of D1R and had no significant effect on D2R, suggesting that D1R may play the main role in regulating the AC/cAMP/PKA pathway. In summary, D1R and D2R corporately mediated the activation of the AC/cAMP/PKA pathway to enhance intracellular Ca^2+^ level. The AMP-independent pathways mediated by DRs binding G_αq/11_ or G_βγ_ might also involve in PKA-mediated signalling.

To further elucidate whether DRs intervention alleviate aconitine-induced neurotoxicity in zebrafish larvaes and SH-SY5Y cells, the effects of D1R antagonist SCH23390 and D2R agonist sumanirole on the swimming behavior and intracellular Ca^2+^ level of zebrafish larvaes were investigated, as well as cell proliferation and the AC/cAMP/PKA signalling pathway in SH-SY5Y cells. SCH23390 and sumanirole had slightly different effects on aconitine-induced abnormal swimming behaviors. SCH23390 significantly inhibited the increase of distance travelled induced by aconitine at 0.5, 3, 6, and 24 h, whereas no significant alteration was detected at 12 h. The increase in the distance travelled of larvaes induced by aconitine possibly reached the peak at 12 h, but SCH23390 pretreatment was insufficient to reverse the extremely significant increase of locomotor activity. To verify this result, we will optimize the concentration and time of SCH23390 pretreatment in the subsequent experiments. Compared with SCH23390, sumanirole had a stronger inhibitory effect on the increase in spontaneous behavior induced by aconitine in zebrafish. The probable reason may be that D2R complete agonist sumanirole not only directly reduces the excitatory behaviors of larvaes through the D2R-mediated AC/cAMP/PKA pathway, but also inhibits the synthesis and release of dopamine by enhancing negative feedback of D2R in the presynaptic membrane, thereby decreasing the neurotransmission of dopamine (Qiao et al., 2020). However, the edge thixotropic behavior induced by aconitine was significantly suppressed by pretreatment with SCH23390, whereas sumanirole did not significantly influence this behavior. Li et al. found that the down-regulation of postsynaptic D2R in the frontal association cortex was linked to anxiety-like behaviour in awake parkinsonian mice (Li et al., 2019). In contrast, studies suggested that the D2R antagonist sumanirole decreased anxiety-like behavior in the VTA of mice with chronic corticosteroids exposure (Peng et al., 2021). Therefore, the effects of D2R on anxiety-like behaviors are quite complex, which may be related to D2R presynaptic inhibition and the D2R postsynaptic signalling pathway. In addition, D2R in different brain regions may have different effects on anxiety behaviors. Furthermore, sumanirole intervention inhibited the increase in intracellular Ca^2+^ induced by aconitine in zebrafish larvaes, while SCH23390 had no significant effect on the increase in intracellular Ca^2+^ induced by aconitine. Co-pretreatment with SCH23390 and sumanirole enhanced the inhibitory effect of aconitine-induced Ca^2+^ elevation compared with sumanirole alone. These findings support the view that DRs have an important role in modulating Ca^2+^ signalling. DR can regulate intracellular Ca^2+^ concentration through Gα_s_/cAMP/PKA signalling or G_q_/PLC pathway, and also directly act on the calcium channels to achieve the regulation of intracellular Ca^2+^ (Bergson et al., 2003; Beaulieu and Gainetdinov, 2011). To verify whether the PKA signalling pathway is involved in aconitine-induced neurological impairment, we examined the effect of PKA inhibitor (H-89) on intracellular Ca^2+^ increase aconitine-induced. The result showed that H-89 intervention effectively inhibited the increase of intracellular Ca^2+^ induced by aconitine in zebrafish larvaes, which further supported this fact that aconitine induced AC/cAMP/PKA pathway activation to increase intracellular Ca^2+^ level. It is supposed that the increased Ca^2+^ may also lead to oxidative stress and cell apoptosis through Ca^2+^ overload (Berridge et al., 2003). Our findings uncover the potential of DRs intervention agents and PKA inhibitor application for aconitine-induced neurotoxicity prevention and therapy. It should be noted that there are still many deficiencies in this study due to the limited scope of the present study. For instance, our study found that aconitine disrupted dopamine homeostasis and induced oxidative stress and apoptosis. However, the relationship between dopamine homeostasis imbalance and the overproduction of ROS and apoptosis have not been studied clearly. In addition, we found that aconitine induced epileptic and anxiety-like abnormal behavior in zebrafish larvaes. Although SCH23390 and sumanirole could significantly reduce the excitatory locomotor behaviors aconitine-induced, and SCH23390 could also significantly reduce the thigmotaxis of larvaes, the roles of DRs on aconitine-induced epilepsy- and anxiety-behaviors of zebrafish larvaes have not been discussed further.

## Conclusion

**FIGURE 9:**
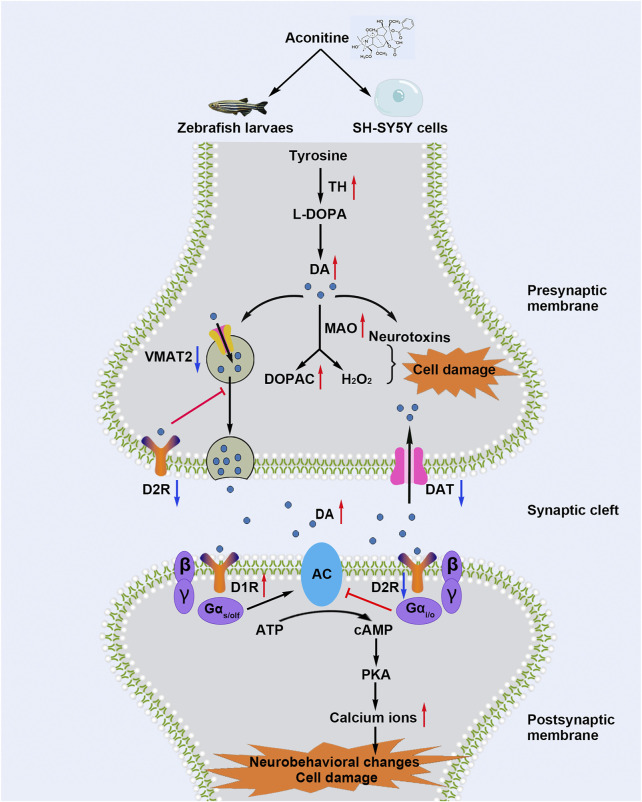
Schematic diagram shows that aconitine induces neurological impairment via dopamine homeostasis disruption and dopamine receptors-mediated AC/cAMP/PKA pathway activation in zebrafish larvaes and SH-SY5Y cells.

Aconitine is a representative and highly toxic compound derived from *Aconitum* plants. Although the neurotoxicity of aconitine has been recognized, the underlying mechanisms have not been elucidated yet. Our study showed that aconitine treatment led to various morphological changes, such as shortened body length, curved body shape, shortened eye distance, and enlarged eyeball, and induced epileptic-like and anxiety-like behaviors of zebrafish larvaes. As shown in Figure 9, aconitine increased dopamine levels in zebrafish larvaes and SH-SY5Y cells and disrupted dopamine homeostasis by regulating the functional proteins of dopamine synthesis, degradation, transport, and reuptake. Moreover, aconitine promoted the AC/cAMP/PKA pathway activation by potentiating D1R or inhibiting D2R, following the increased intracellular Ca^2+^ level, eventually resulting in dopaminergic dysfunction. To our knowledge, our study is the first to demonstrate that dopamine homeostasis imbalance and DRs-mediated AC/cAMP/PKA signalling pathway activation are involved in aconitine-induced neurological impairment.

## Data Availability

The original contributions presented in the study are included in the article/Supplementary Material, further inquiries can be directed to the corresponding authors.

## Glossary

AC: adenylate cyclase
ANOVA: one-way analysis of variance
BCA: bicinchoninic acid
cAMP: cyclic adenosine monophosphate
CI: confidence intervals
CW: clockwise
D1R: dopamine D1 Receptor
D2R: dopamine D2 Receptor
DAQ: dopamine o-quinone
DASQ: dopamine semiquinone
DAT: dopamine transporter
DCFH-DA: 2,7-dichlorofluorescein diacetate
DDAs: diester diterpence-type aconitum alkaloids
DEPC: diethylpyrocarbonate
DMSO: dimethylsulfoxide
DOPAC: 3, 4-dihydroxyphenylacetic acid
dpf: day post-fertilization
DRs: dopamine receptors
GPCRs: G-protein-coupled receptors
HPLC: high performance liquid chromatography
HRP: horseradish peroxidase
HVA: homovanilic acid
LC50: median lethal concentration
LSCM: laser scanning confocal microscope
LSD: least significant difference
MAO: monoamine oxidase
PFA: paraformaldehyde
PKA: protein kinase A
PMSF: phenylmethanesulfonyl fluoride
PTU: 1-phenyl 2-thiourea
ROS: reactive oxygen species
RT-qPCR: real-time quantitative polymerase chain reaction
SD: standard deviation
TH: tyrosine hydroxylase
TUNEL: terminal deoxynucleotidyl transferase-mediated dUTP nick end labeling
VMAT2: vesicular monoamine transporter type 2.
