# Corrigendum: Dopamine homeostasis imbalance and dopamine receptors-mediated AC/cAMP/PKA pathway activation are involved in aconitine-induced neurological impairment in zebrafish and SH-SY5Y cells

**DOI:** 10.3389/fphar.2023.1219561

**Published:** 2023-05-16

**Authors:** Jie Zhou, Cheng Peng, Qiuju Li, Xiaoyu Yan, Liang Yang, Mengting Li, Xiaoyu Cao, Xiaofang Xie, Dayi Chen, Chaolong Rao, Sizhou Huang, Fu Peng, Xiaoqi Pan

**Affiliations:** ^1^ State Key Laboratory of Southwestern Chinese Medicine Resource, School of Pharmacy and School of Public Health, Chengdu University of Traditional Chinese Medicine, Chengdu, China; ^2^ Department of Pharmacy, The Affiliated Hospital of Southwest Medical University, Luzhou, China; ^3^ Development and Regeneration Key Laboratory of Sichuan Province, Department of Anatomy and Histology and Embryology, School of Basic Medicine, Chengdu Medical College, Chengdu, China; ^4^ West China School of Pharmacy, State Key Laboratory of Biotherapy, West China Hospital, Sichuan University, Chengdu, China

**Keywords:** aconitine, dopamine homeostasis, dopamine receptor, AC/cAMP/PKA, neurological impairment

In the published article, there was an error in **Affiliation 1**. Instead of “Key Laboratory of Southwestern Chinese Medicine Resources,” it should be “State Key Laboratory of Southwestern Chinese Medicine Resource.”

In the published article, there was an error in **Affiliation 2**. Instead of “Department of Pharmacy, Affiliated Hospital of Southwest Medical University,” it should be “Department of Pharmacy, The Affiliated Hospital of Southwest Medical University.”

The authors apologize for this error and state that this does not change the scientific conclusions of the article in any way. The original article has been updated.

