# Supplementary figures and images for: Dopamine Homeostasis Imbalance and Dopamine Receptors-Mediated AC/cAMP/PKA Pathway Activation are Involved in Aconitine-Induced Neurological Impairment in Zebrafish and SH-SY5Y Cells (part 1 of 2)
Source: Front Pharmacol. 2022 Mar 18;13:837810. doi: 10.3389/fphar.2022.837810 (PMC8971779; doi:10.3389/fphar.2022.837810)

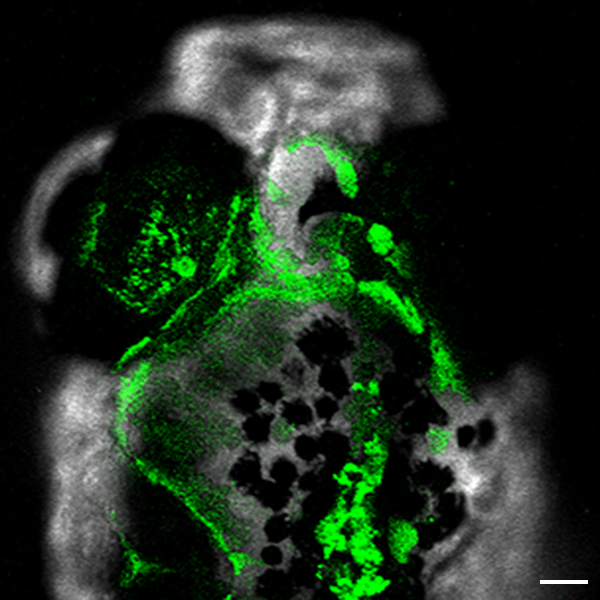

Supplement: Supplementary file 1 [file DataSheet3.zip › Original date(Figure 7-2)/Calcium ion/Aconitine-1.tif]

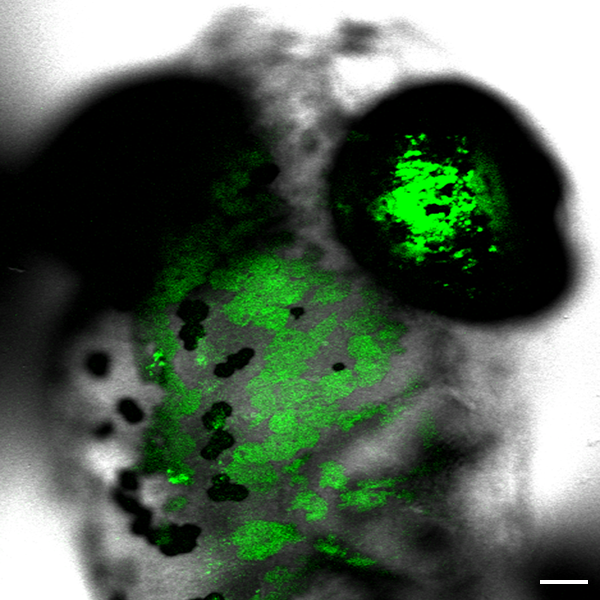

Supplement: Supplementary file 1 [file DataSheet3.zip › Original date(Figure 7-2)/Calcium ion/Aconitine-2.tif]

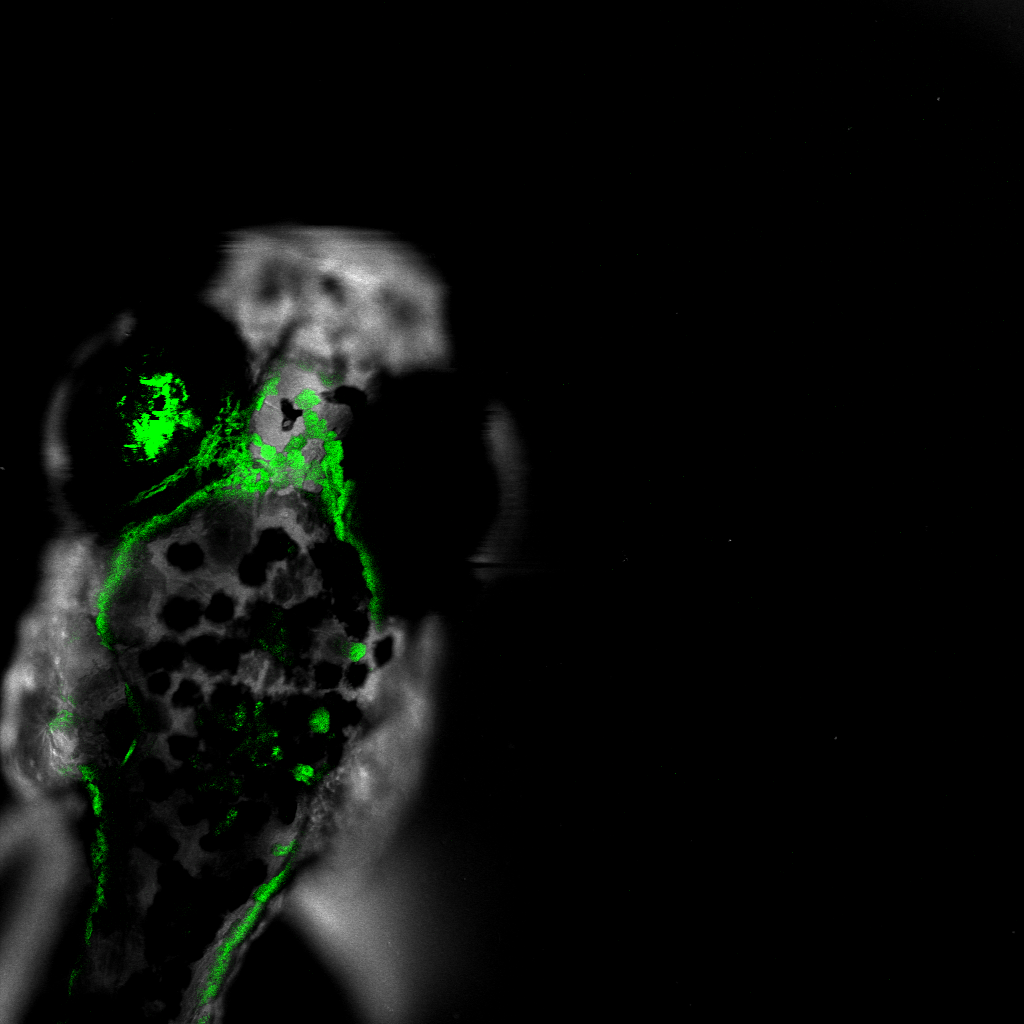

Supplement: Supplementary file 1 [file DataSheet3.zip › Original date(Figure 7-2)/Calcium ion/Aconitine-3.tif]

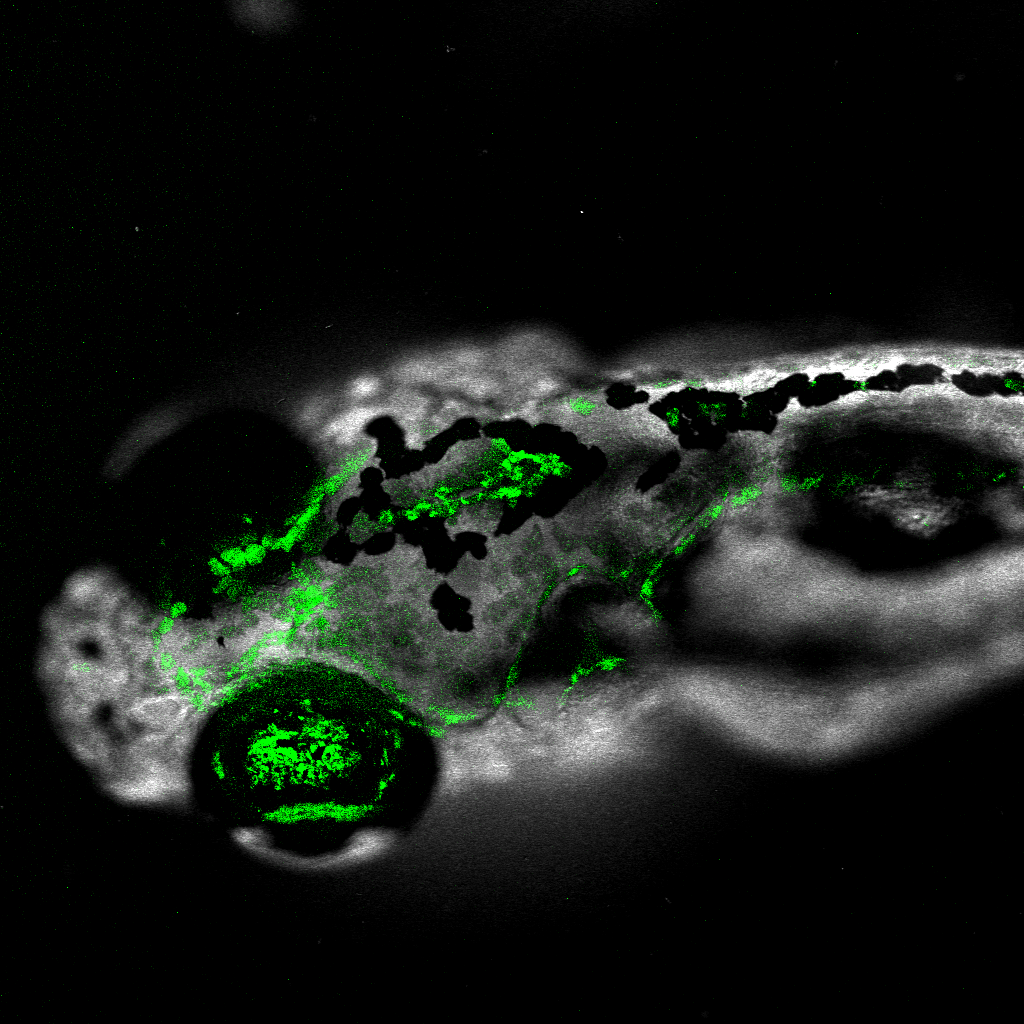

Supplement: Supplementary file 1 [file DataSheet3.zip › Original date(Figure 7-2)/Calcium ion/Aconitine-4.tif]

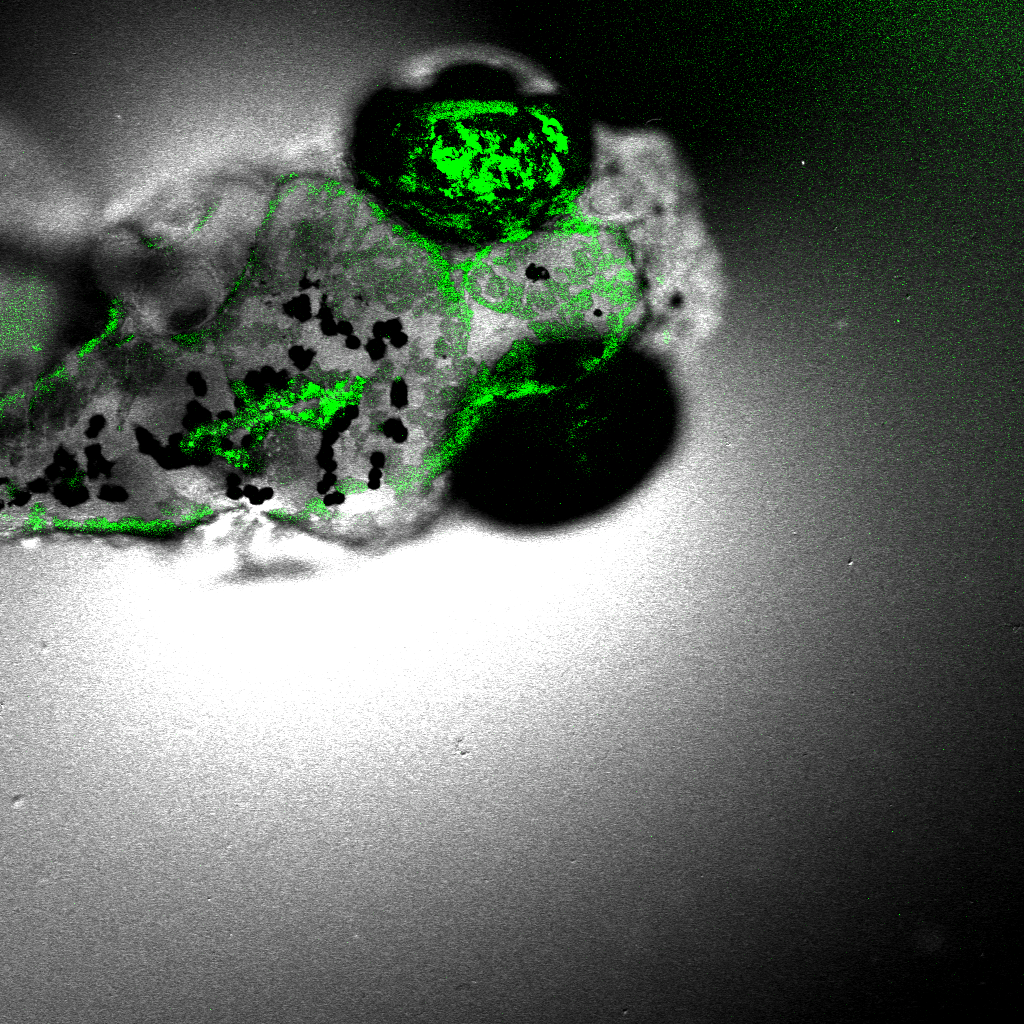

Supplement: Supplementary file 1 [file DataSheet3.zip › Original date(Figure 7-2)/Calcium ion/Aconitine-5.tif]

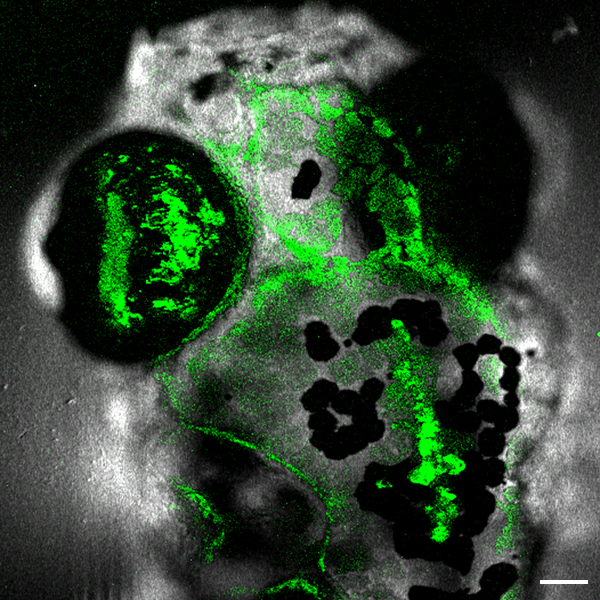

Supplement: Supplementary file 1 [file DataSheet3.zip › Original date(Figure 7-2)/Calcium ion/Aconitine-6.tif]

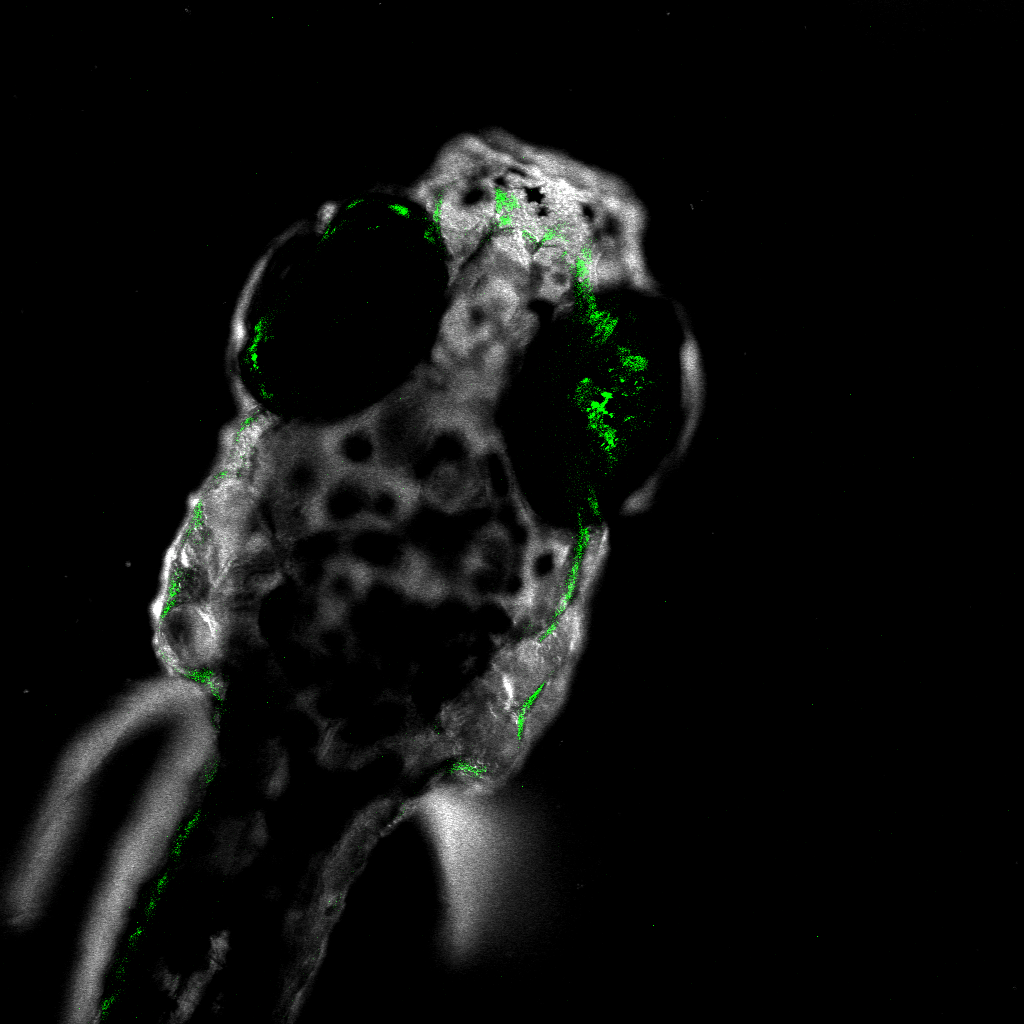

Supplement: Supplementary file 1 [file DataSheet3.zip › Original date(Figure 7-2)/Calcium ion/Control-1.tif]

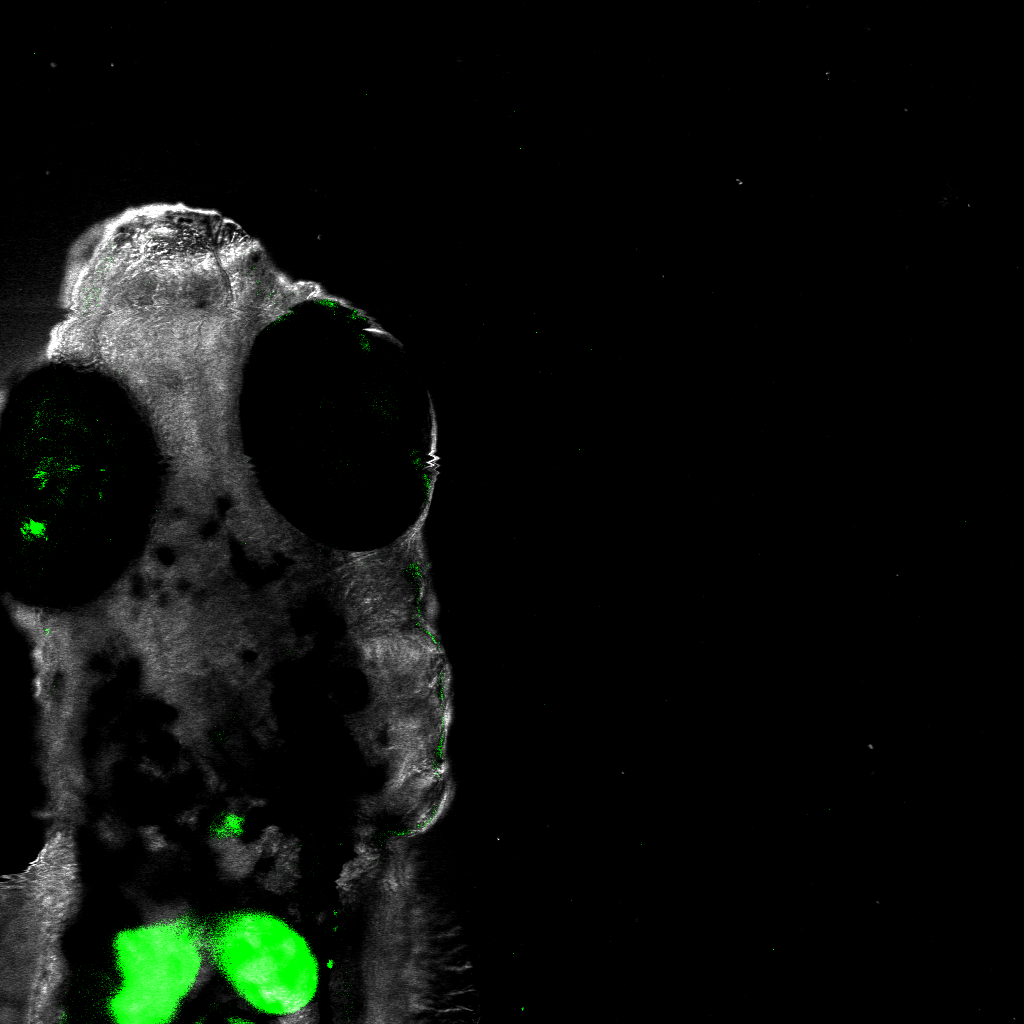

Supplement: Supplementary file 1 [file DataSheet3.zip › Original date(Figure 7-2)/Calcium ion/Control-10.tif]

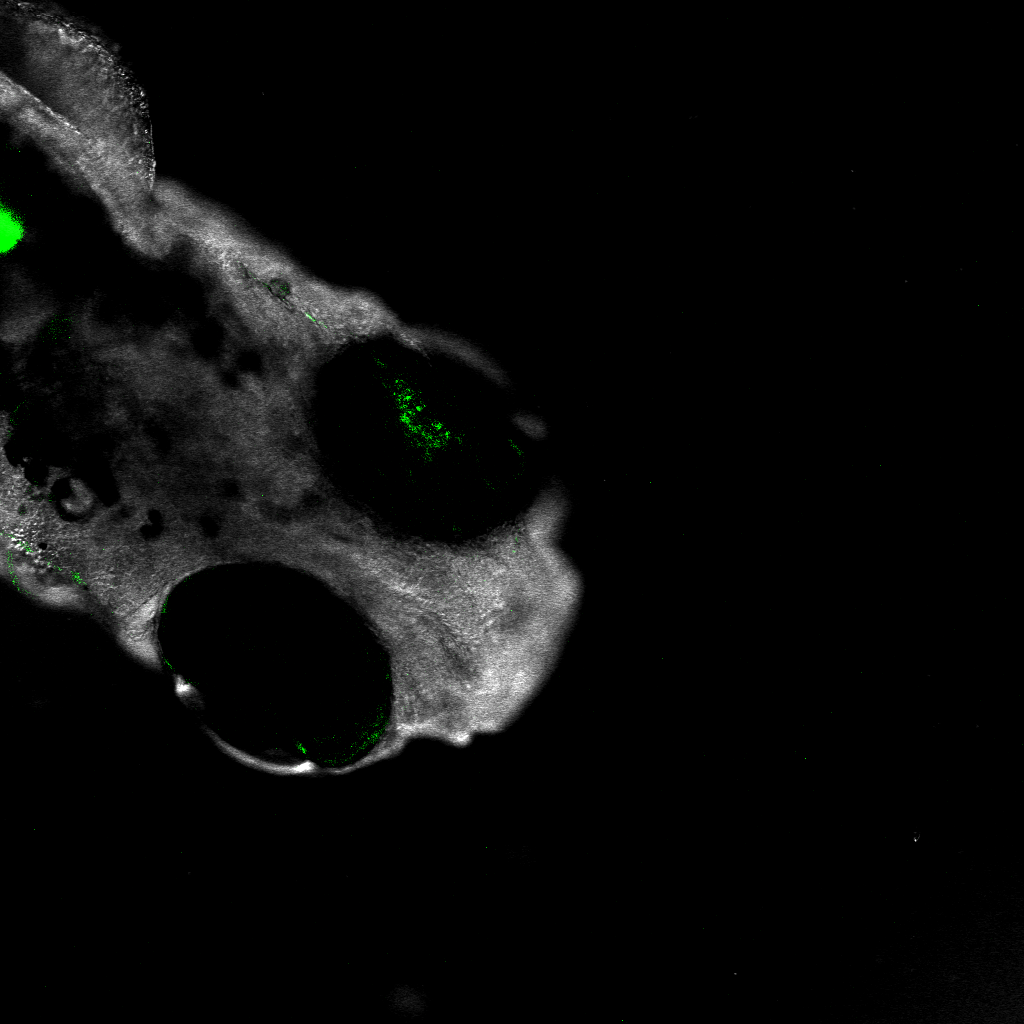

Supplement: Supplementary file 1 [file DataSheet3.zip › Original date(Figure 7-2)/Calcium ion/Control-11.tif]

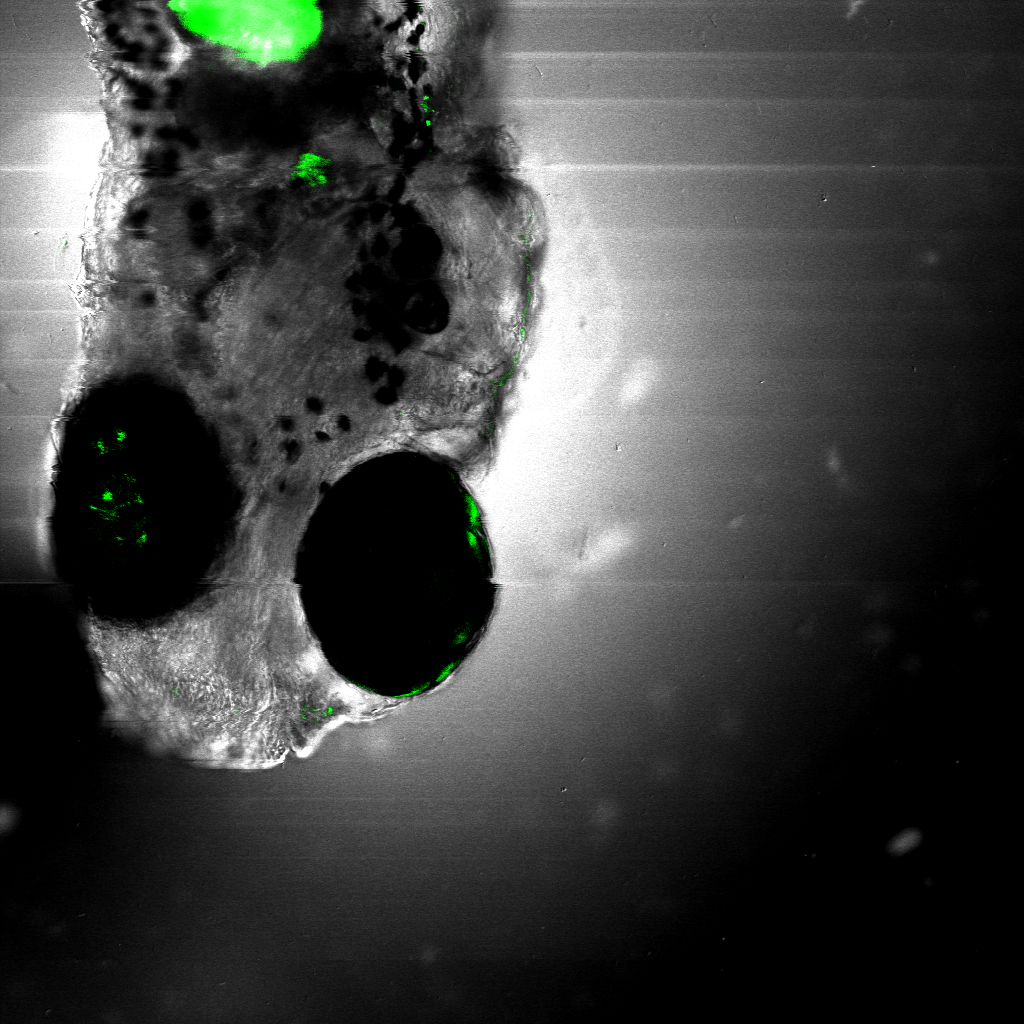

Supplement: Supplementary file 1 [file DataSheet3.zip › Original date(Figure 7-2)/Calcium ion/Control-12.tif]

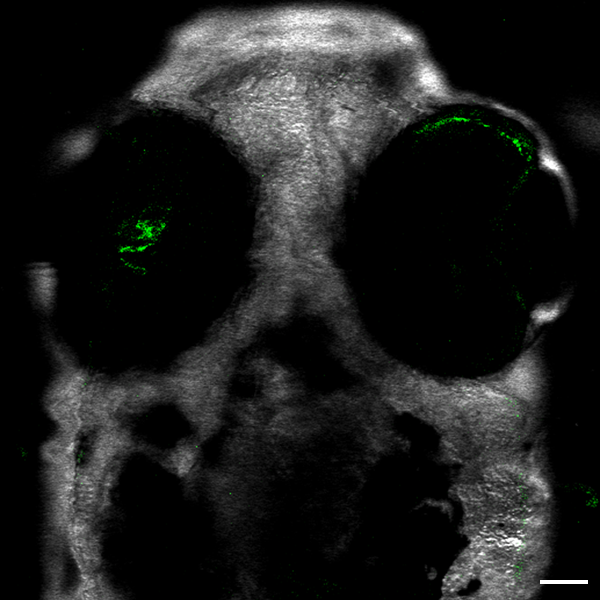

Supplement: Supplementary file 1 [file DataSheet3.zip › Original date(Figure 7-2)/Calcium ion/Control-2.tif]

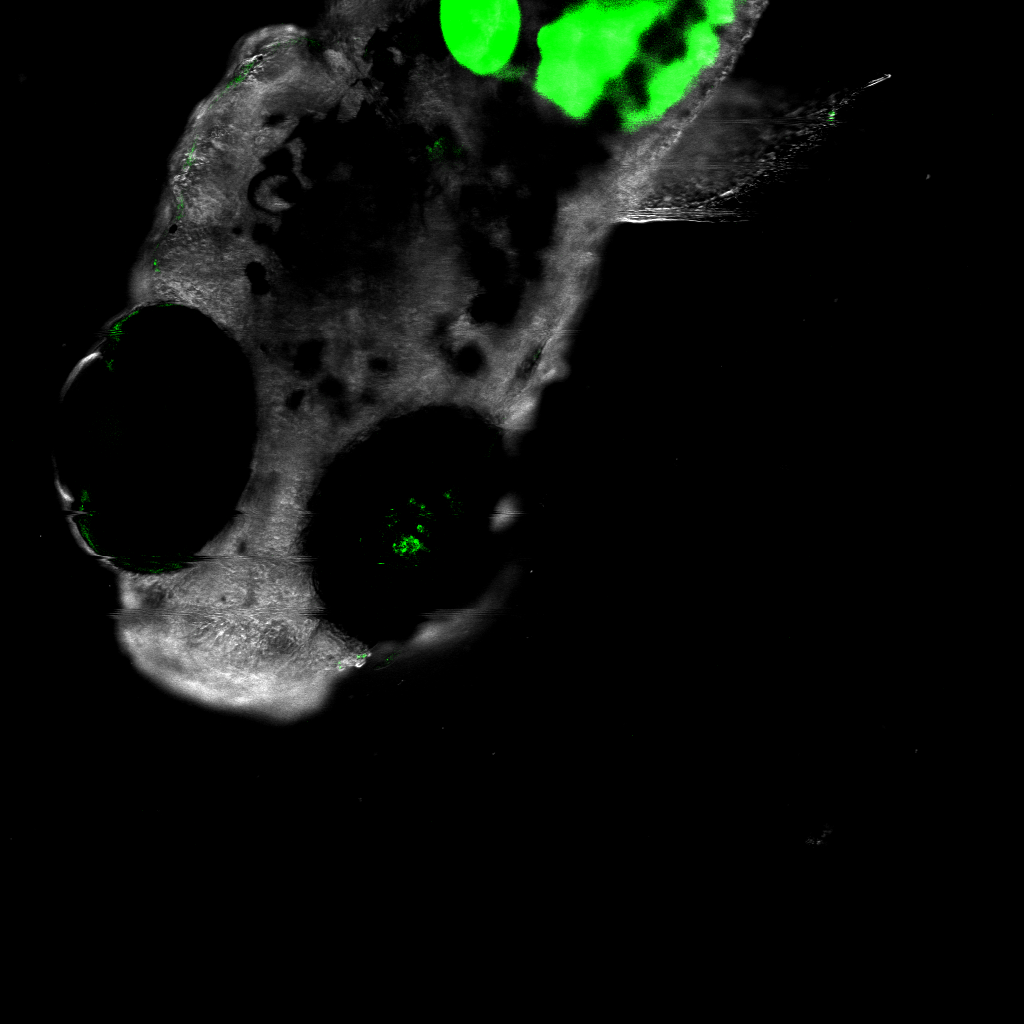

Supplement: Supplementary file 1 [file DataSheet3.zip › Original date(Figure 7-2)/Calcium ion/Control-3.tif]

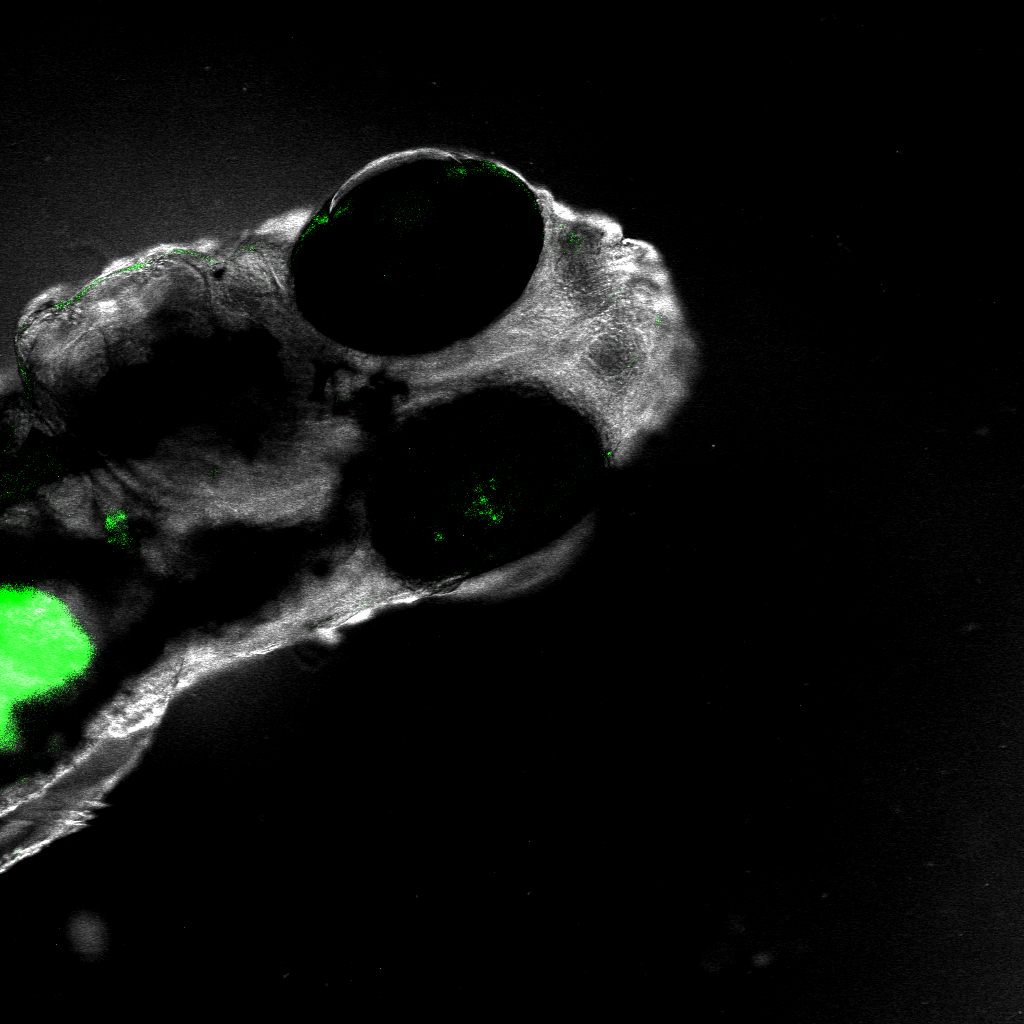

Supplement: Supplementary file 1 [file DataSheet3.zip › Original date(Figure 7-2)/Calcium ion/Control-4.tif]

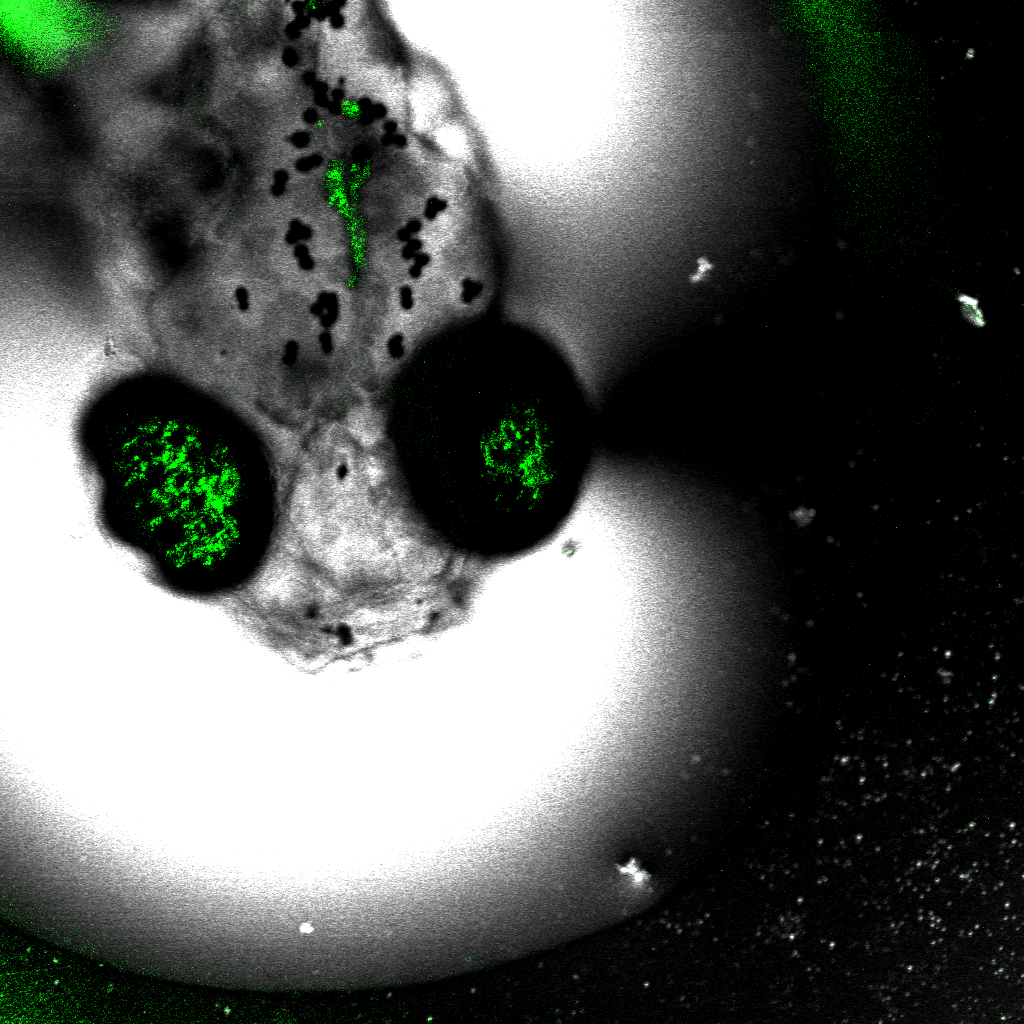

Supplement: Supplementary file 1 [file DataSheet3.zip › Original date(Figure 7-2)/Calcium ion/Control-5.tif]

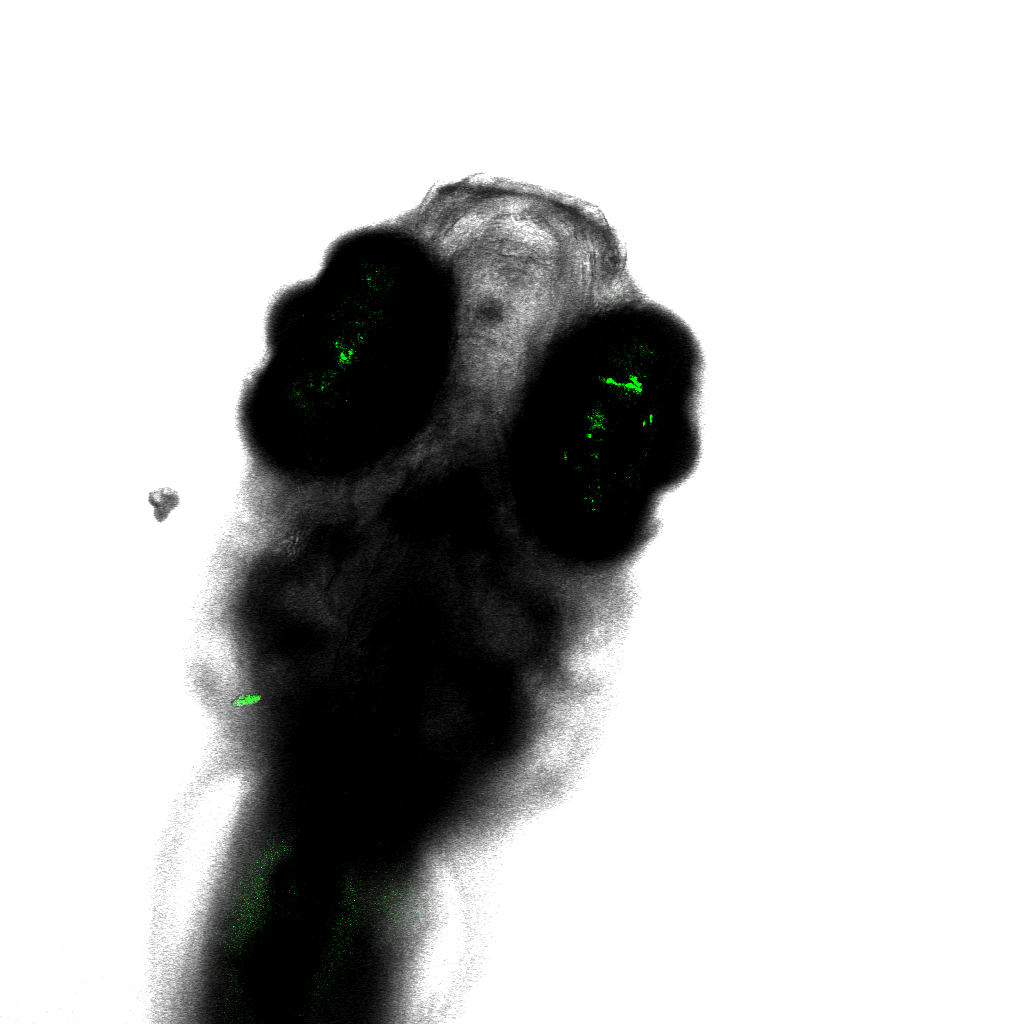

Supplement: Supplementary file 1 [file DataSheet3.zip › Original date(Figure 7-2)/Calcium ion/Control-6.tif]

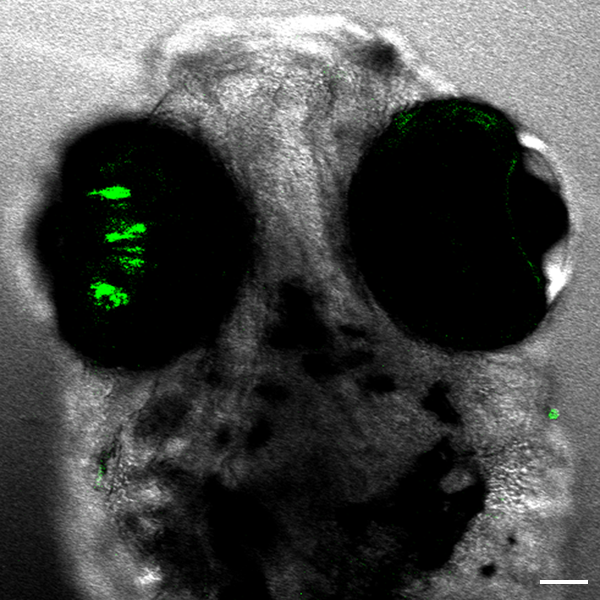

Supplement: Supplementary file 1 [file DataSheet3.zip › Original date(Figure 7-2)/Calcium ion/Control-7.tif]

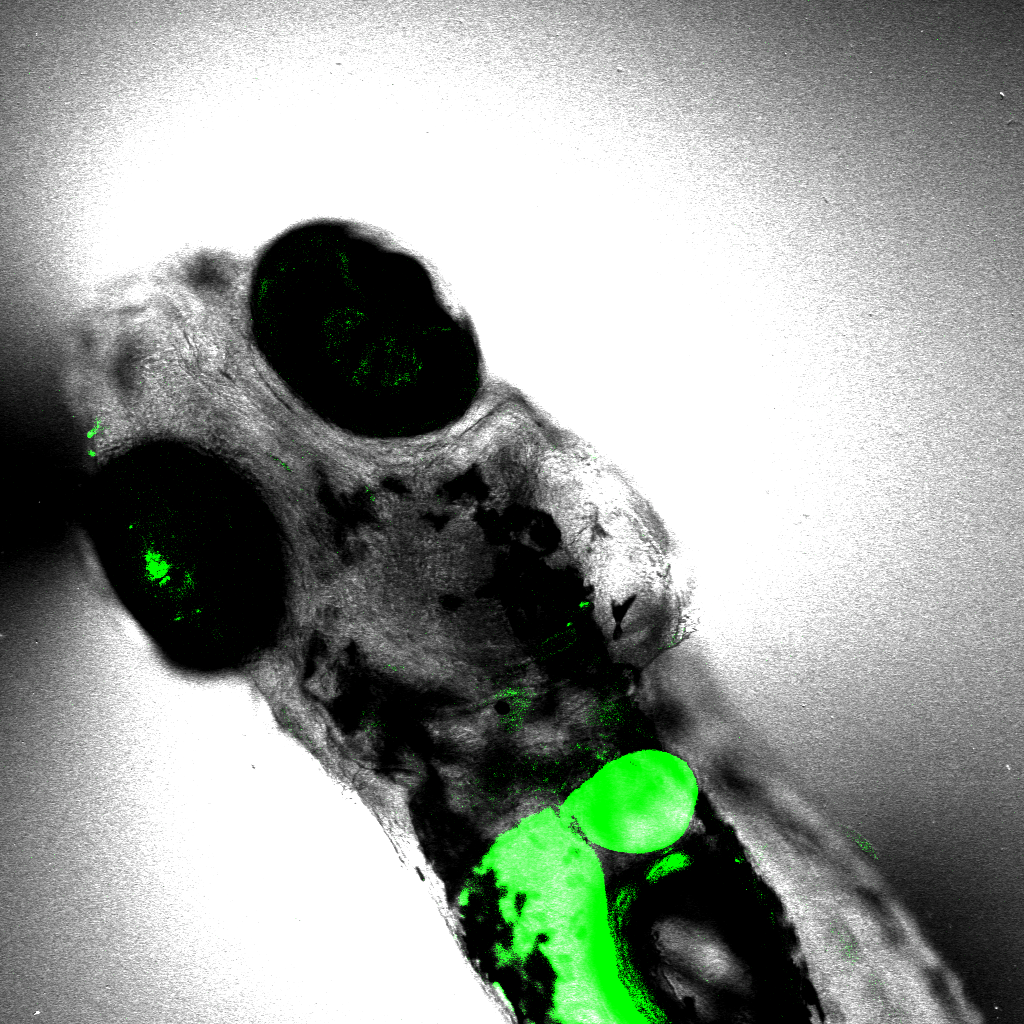

Supplement: Supplementary file 1 [file DataSheet3.zip › Original date(Figure 7-2)/Calcium ion/Control-8.tif]

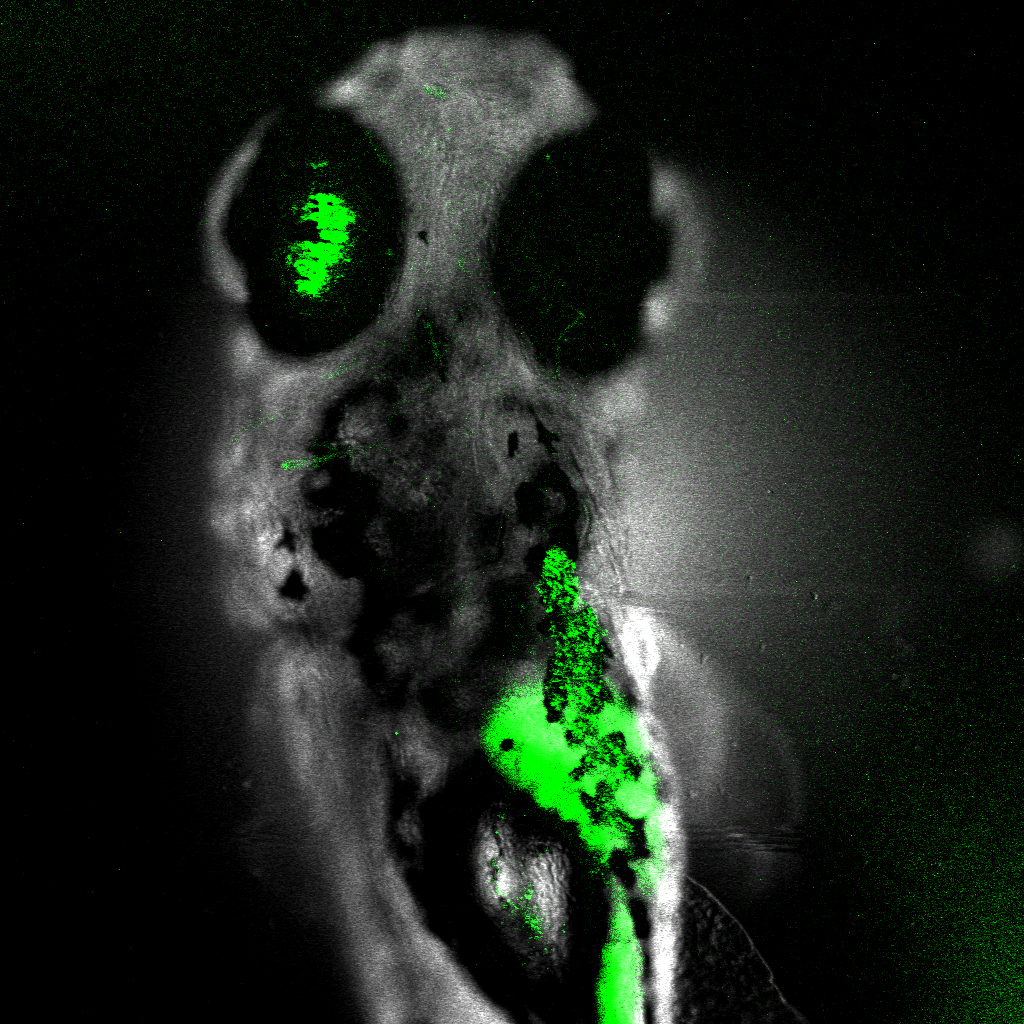

Supplement: Supplementary file 1 [file DataSheet3.zip › Original date(Figure 7-2)/Calcium ion/Control-9.tif]

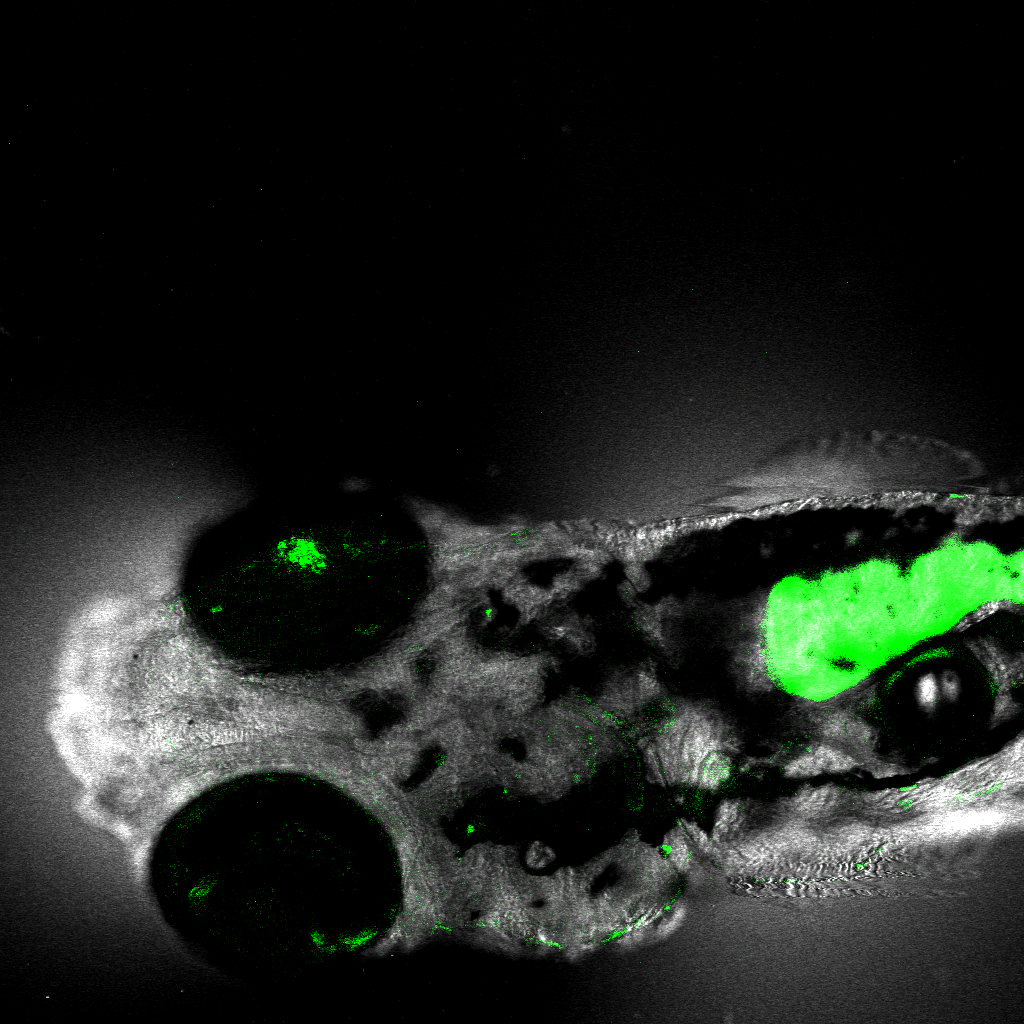

Supplement: Supplementary file 1 [file DataSheet3.zip › Original date(Figure 7-2)/Calcium ion/H89+Aconitine-1.tif]

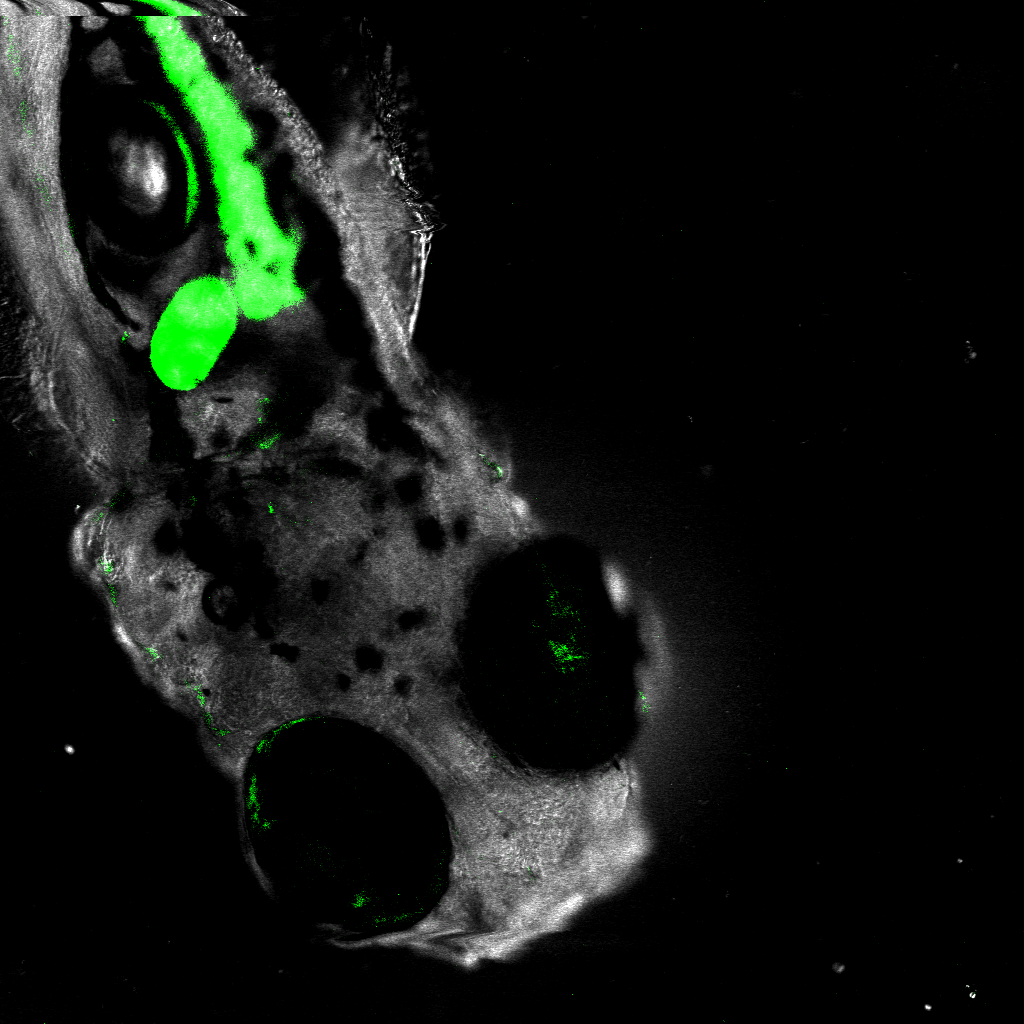

Supplement: Supplementary file 1 [file DataSheet3.zip › Original date(Figure 7-2)/Calcium ion/H89+Aconitine-2.tif]

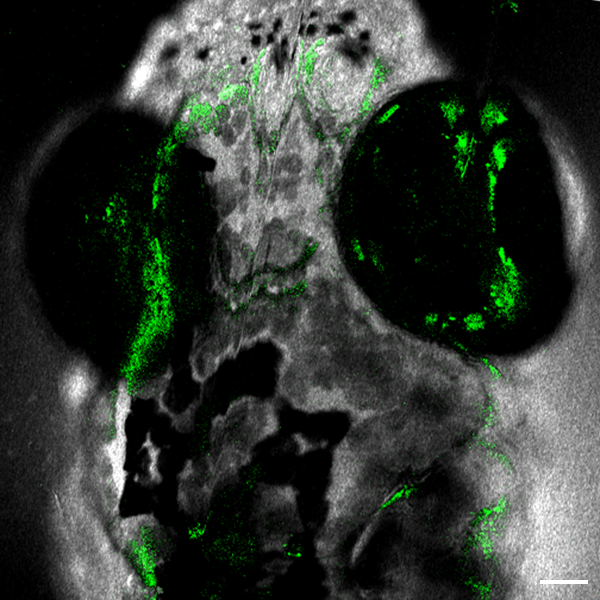

Supplement: Supplementary file 1 [file DataSheet3.zip › Original date(Figure 7-2)/Calcium ion/H89+Aconitine-3.tif]

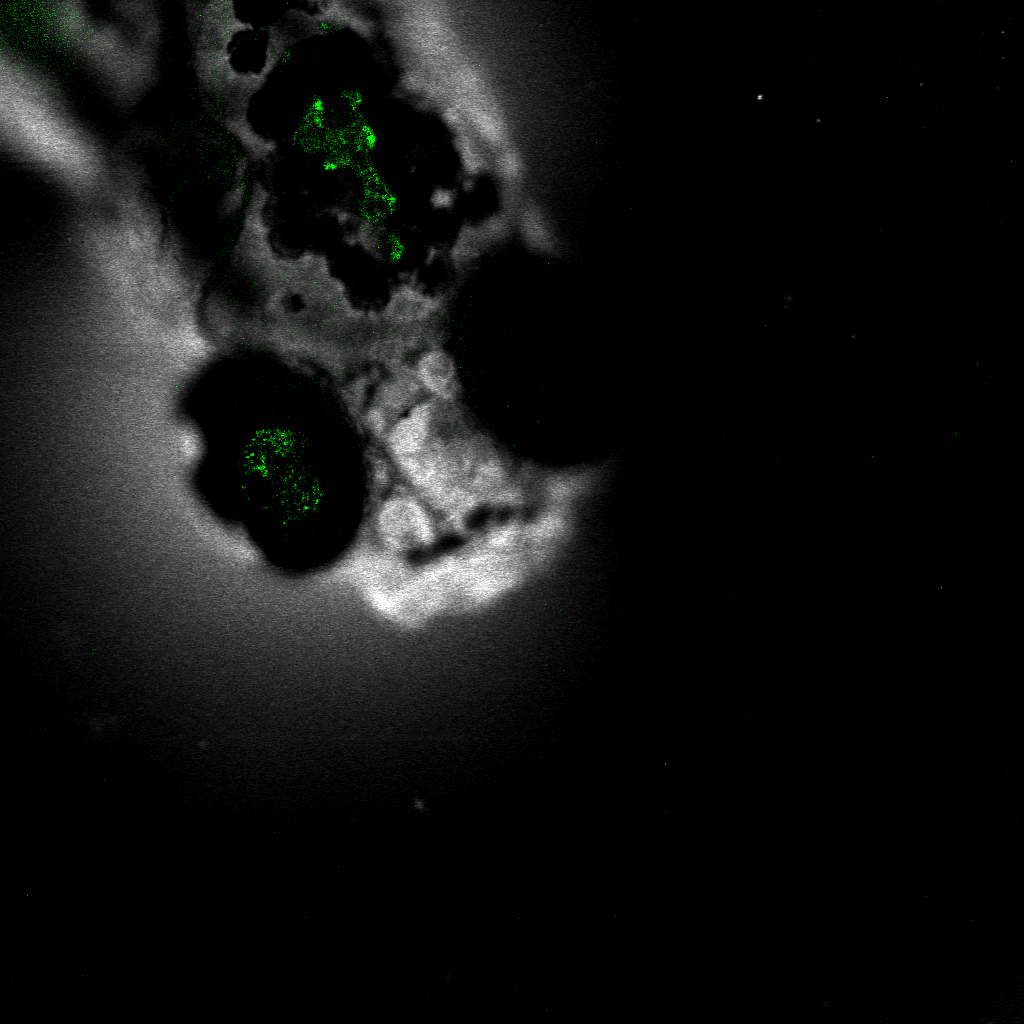

Supplement: Supplementary file 1 [file DataSheet3.zip › Original date(Figure 7-2)/Calcium ion/H89+Aconitine-4.tif]

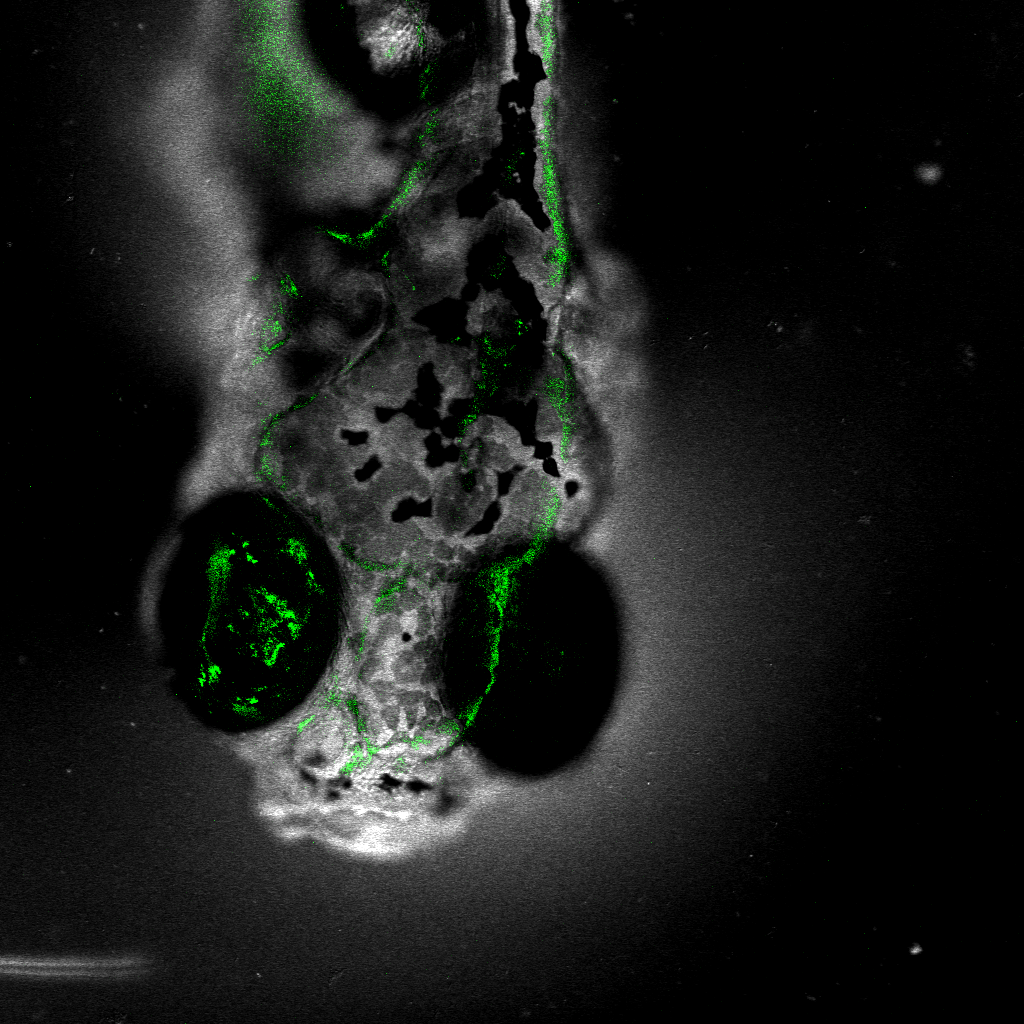

Supplement: Supplementary file 1 [file DataSheet3.zip › Original date(Figure 7-2)/Calcium ion/H89+Aconitine-5.tif]

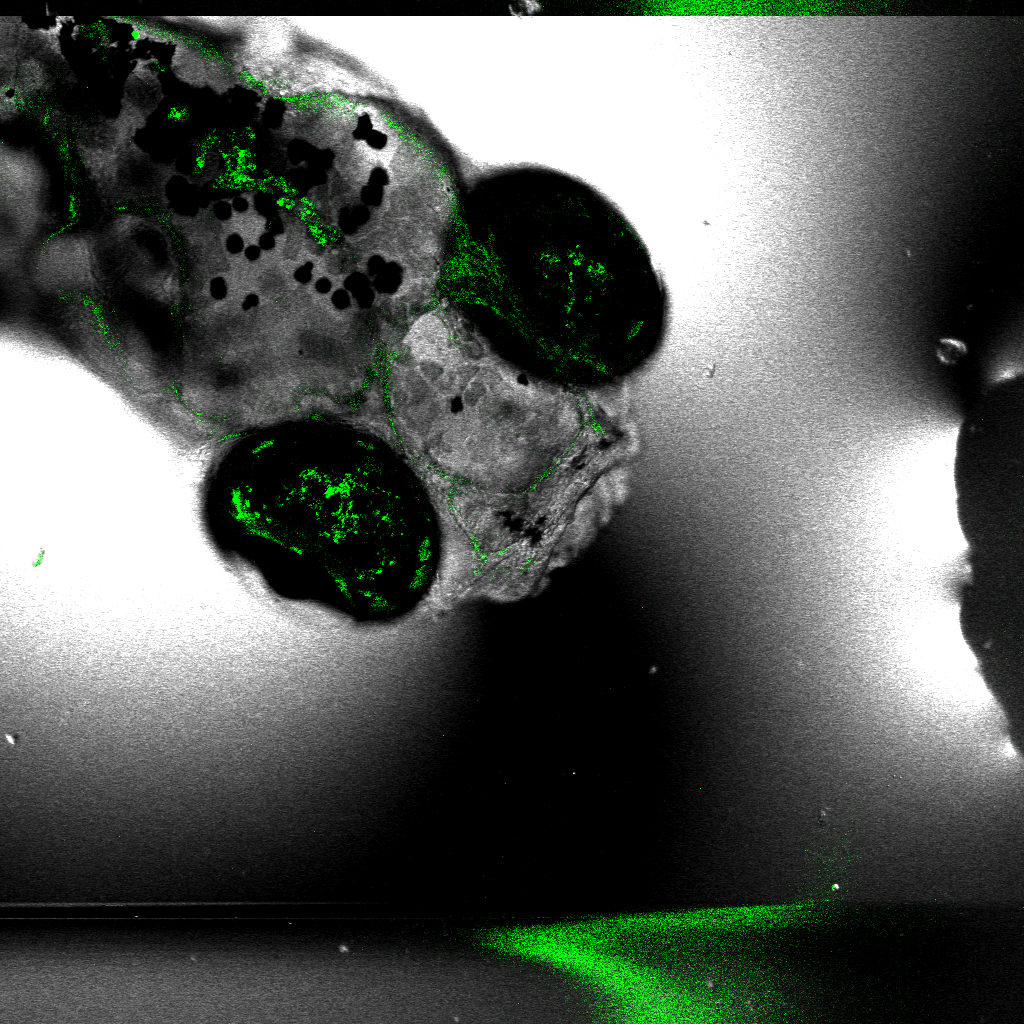

Supplement: Supplementary file 1 [file DataSheet3.zip › Original date(Figure 7-2)/Calcium ion/H89+Aconitine-6.tif]

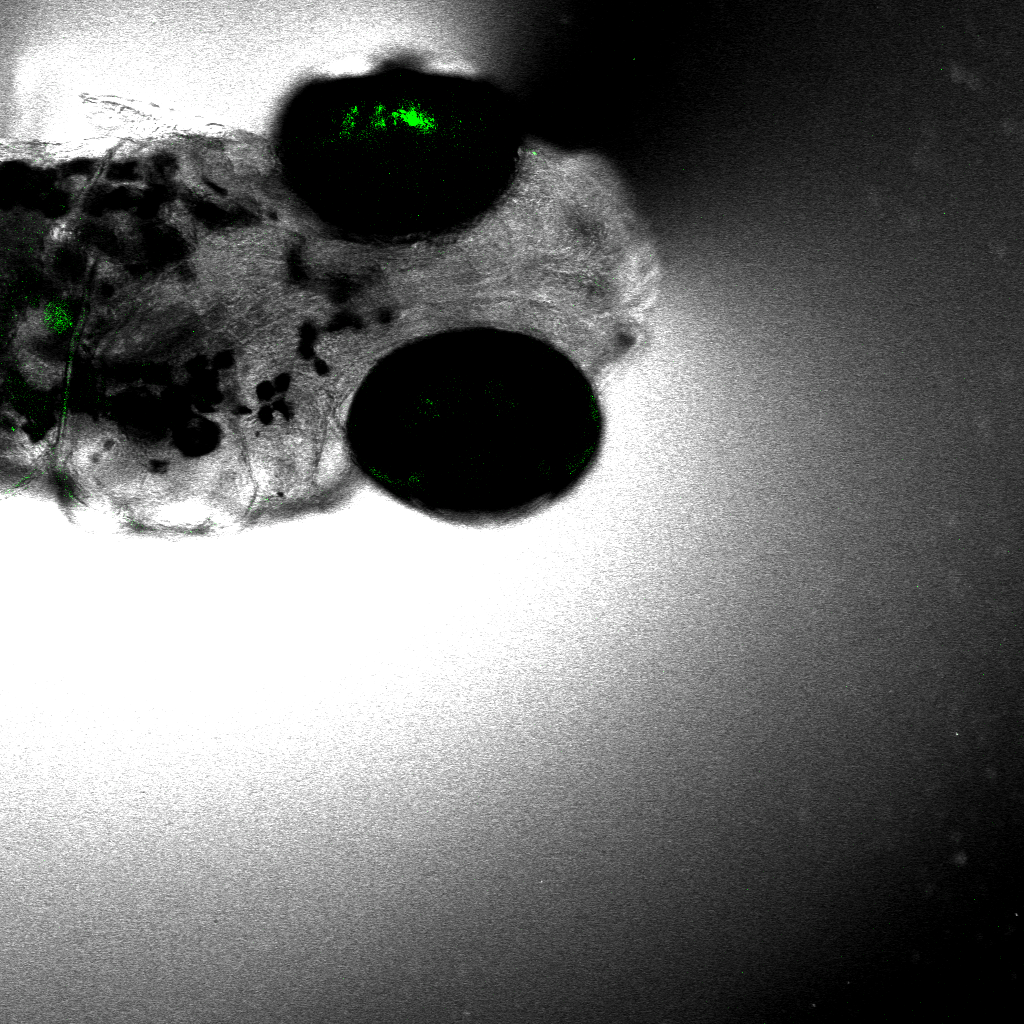

Supplement: Supplementary file 1 [file DataSheet3.zip › Original date(Figure 7-2)/Calcium ion/H89-1.tif]

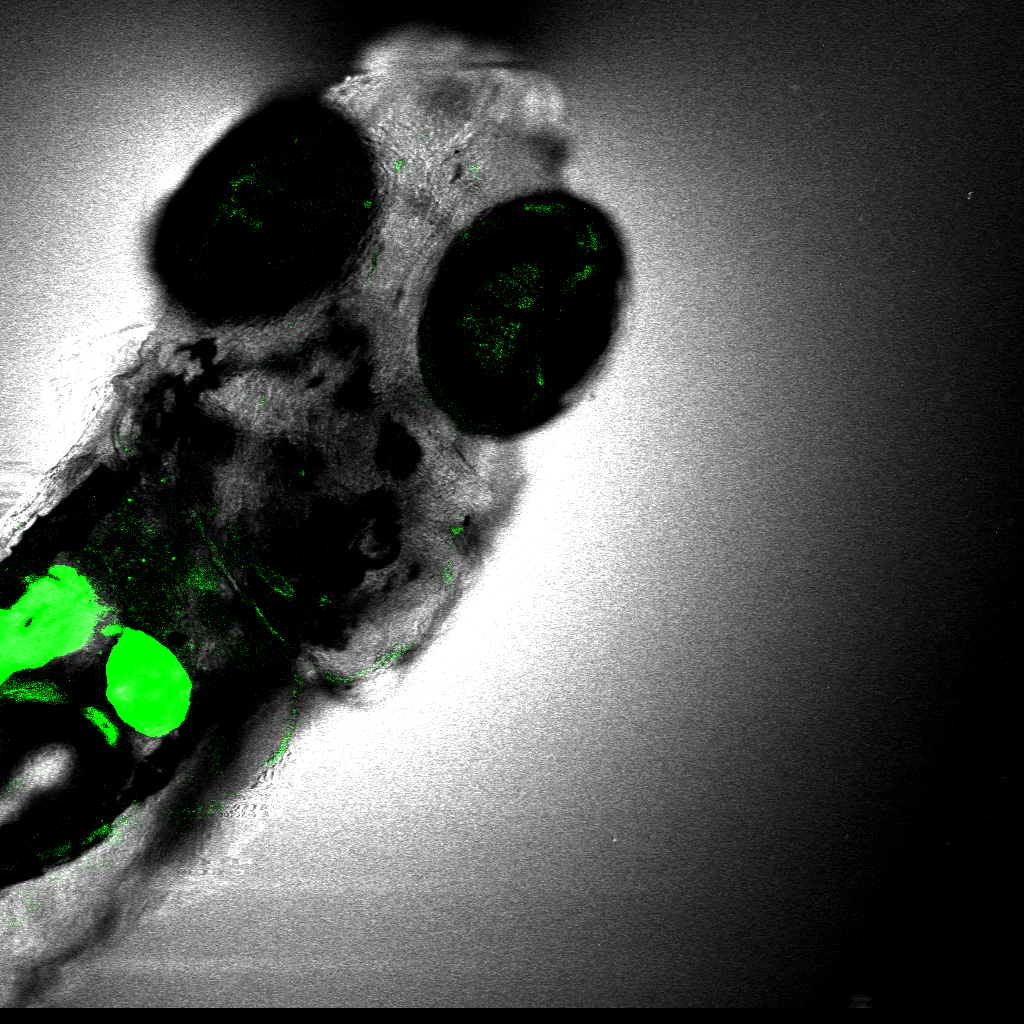

Supplement: Supplementary file 1 [file DataSheet3.zip › Original date(Figure 7-2)/Calcium ion/H89-2.tif]

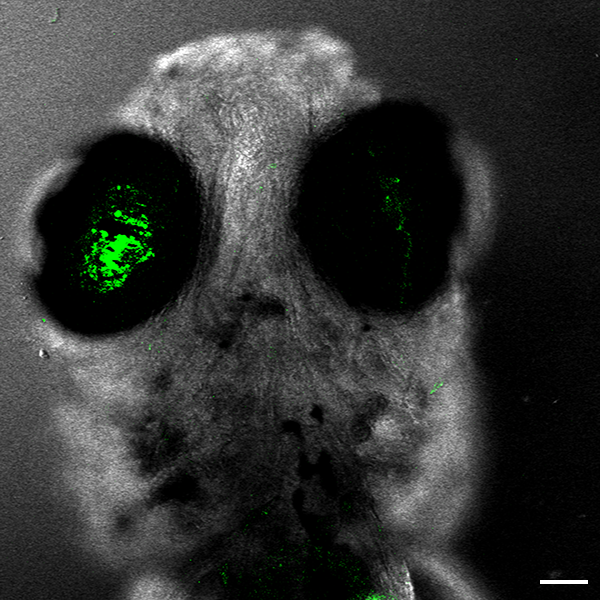

Supplement: Supplementary file 1 [file DataSheet3.zip › Original date(Figure 7-2)/Calcium ion/H89-4.tif]

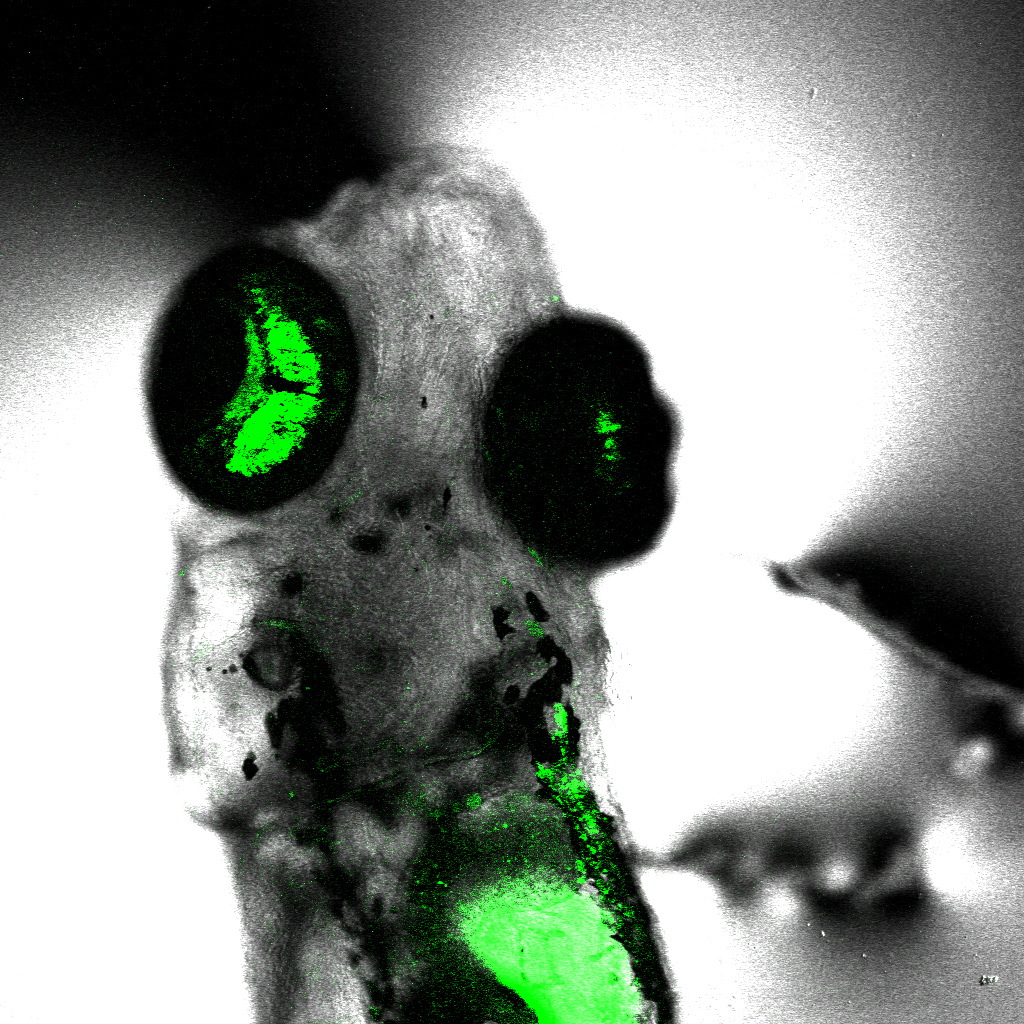

Supplement: Supplementary file 1 [file DataSheet3.zip › Original date(Figure 7-2)/Calcium ion/H89-5.tif]

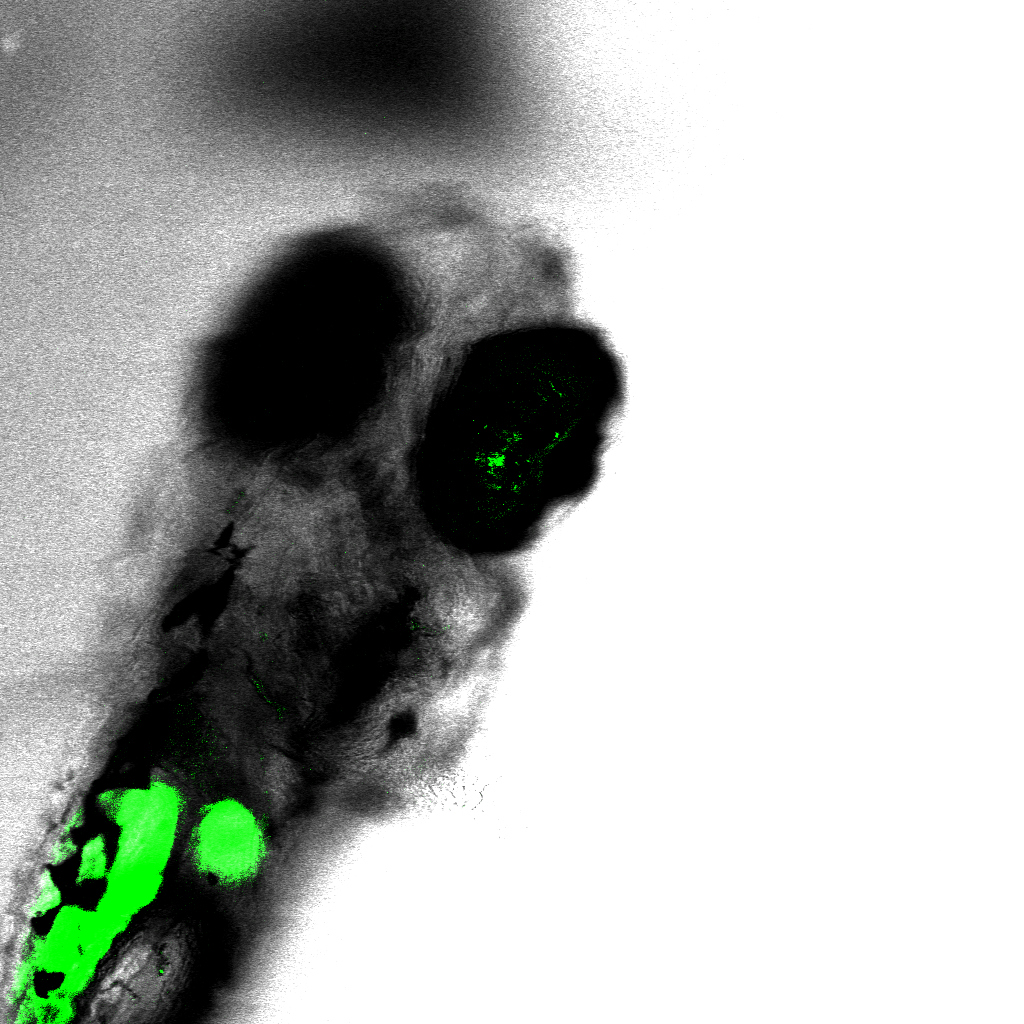

Supplement: Supplementary file 1 [file DataSheet3.zip › Original date(Figure 7-2)/Calcium ion/H89-6.tif]

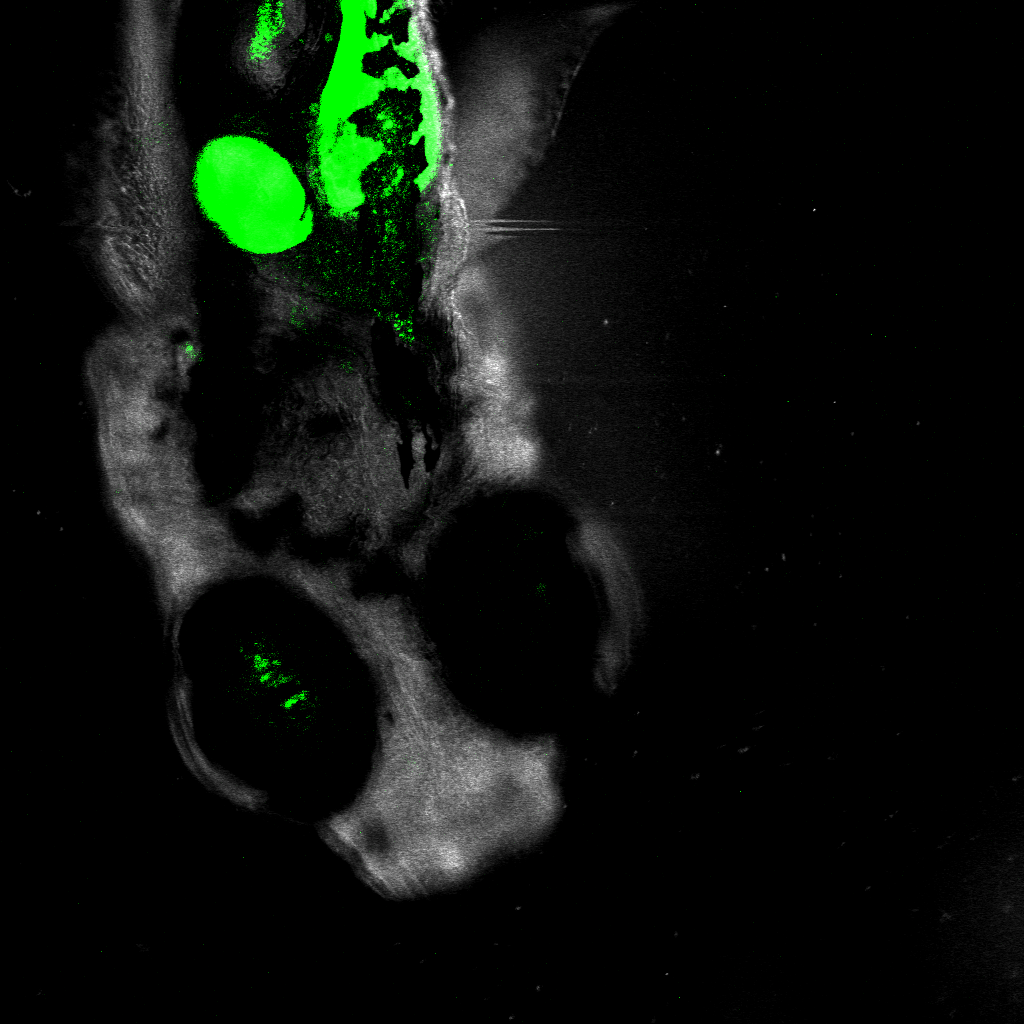

Supplement: Supplementary file 1 [file DataSheet3.zip › Original date(Figure 7-2)/Calcium ion/H89-8.tif]

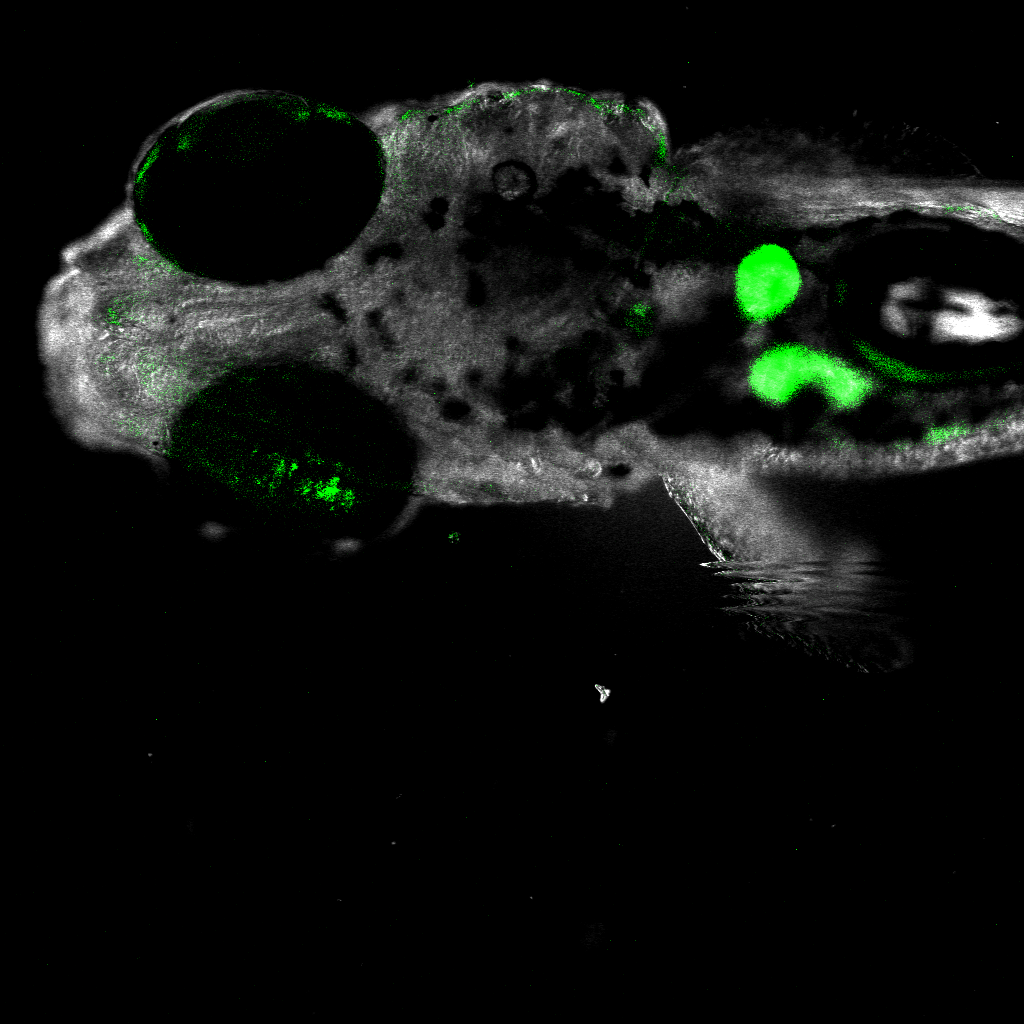

Supplement: Supplementary file 1 [file DataSheet3.zip › Original date(Figure 7-2)/Calcium ion/SCH23390+Sumanirole+Aconitine-(1).tif]

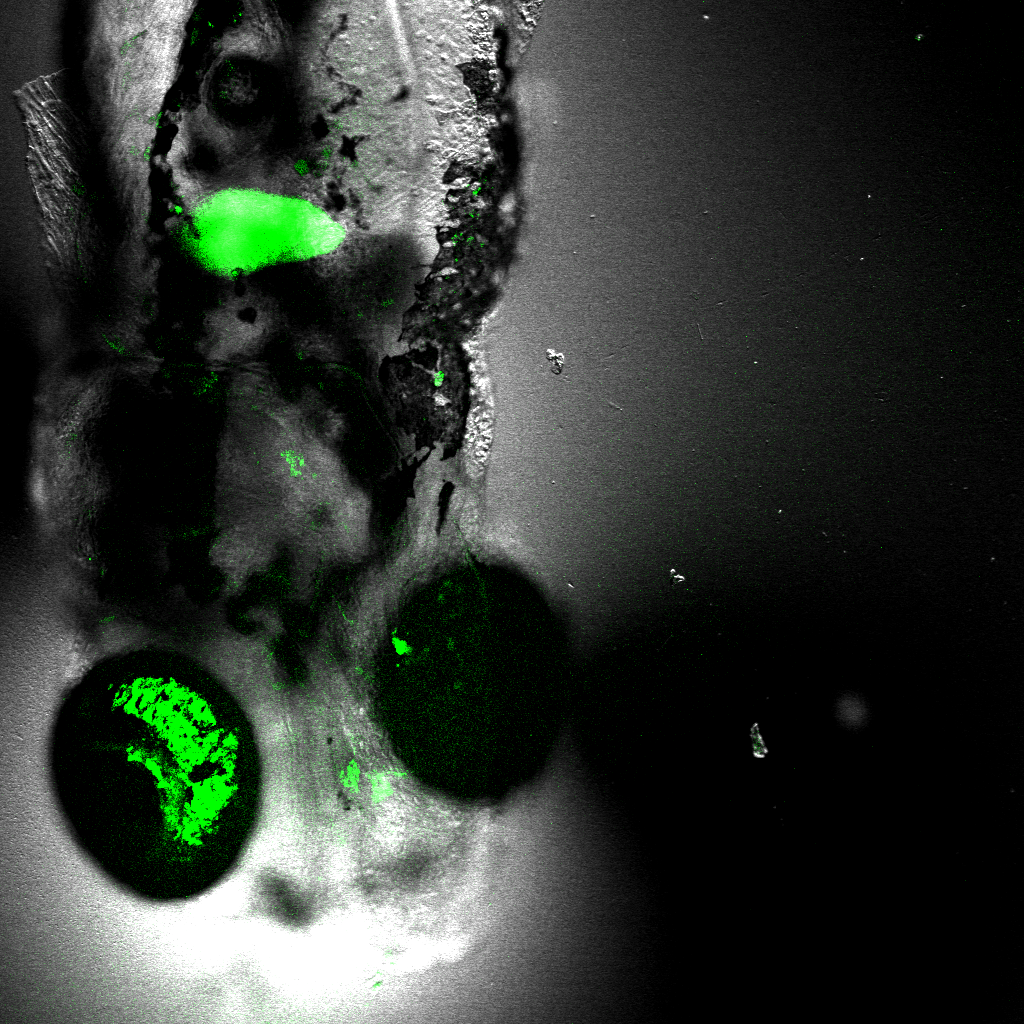

Supplement: Supplementary file 1 [file DataSheet3.zip › Original date(Figure 7-2)/Calcium ion/SCH23390+Sumanirole+Aconitine-(2).tif]

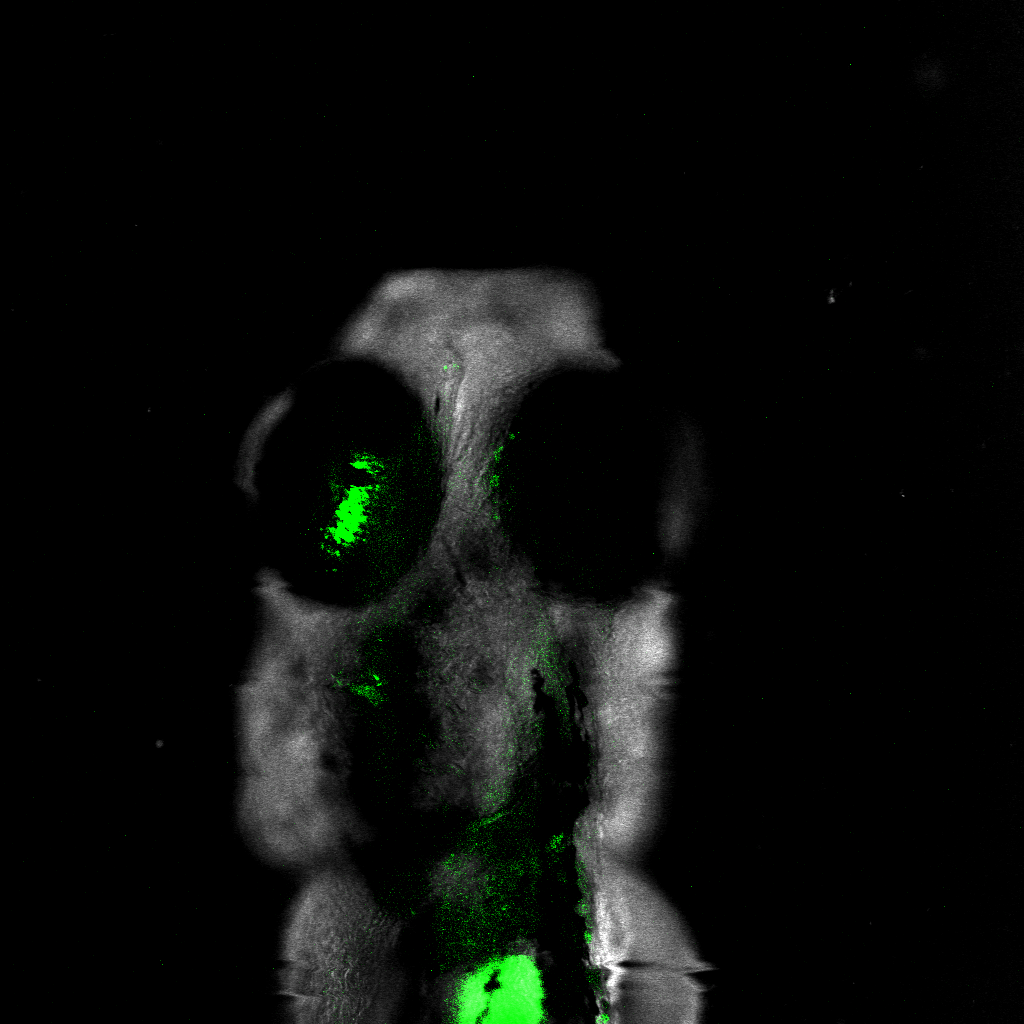

Supplement: Supplementary file 1 [file DataSheet3.zip › Original date(Figure 7-2)/Calcium ion/SCH23390+Sumanirole+Aconitine-(3).tif]

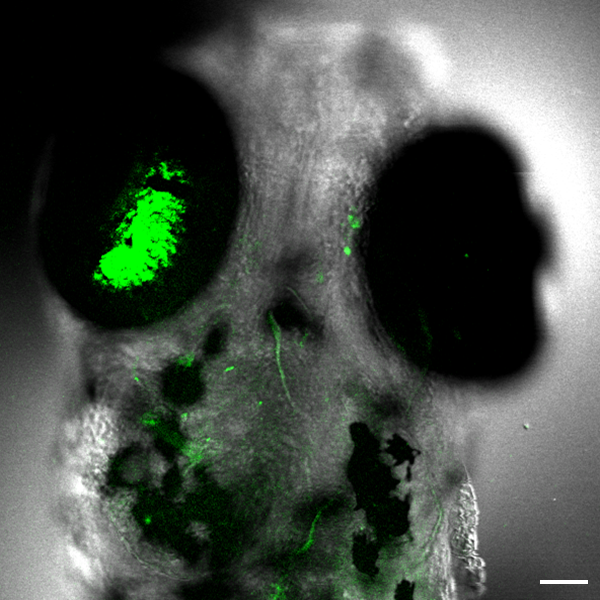

Supplement: Supplementary file 1 [file DataSheet3.zip › Original date(Figure 7-2)/Calcium ion/SCH23390+Sumanirole+Aconitine-(4).tif]

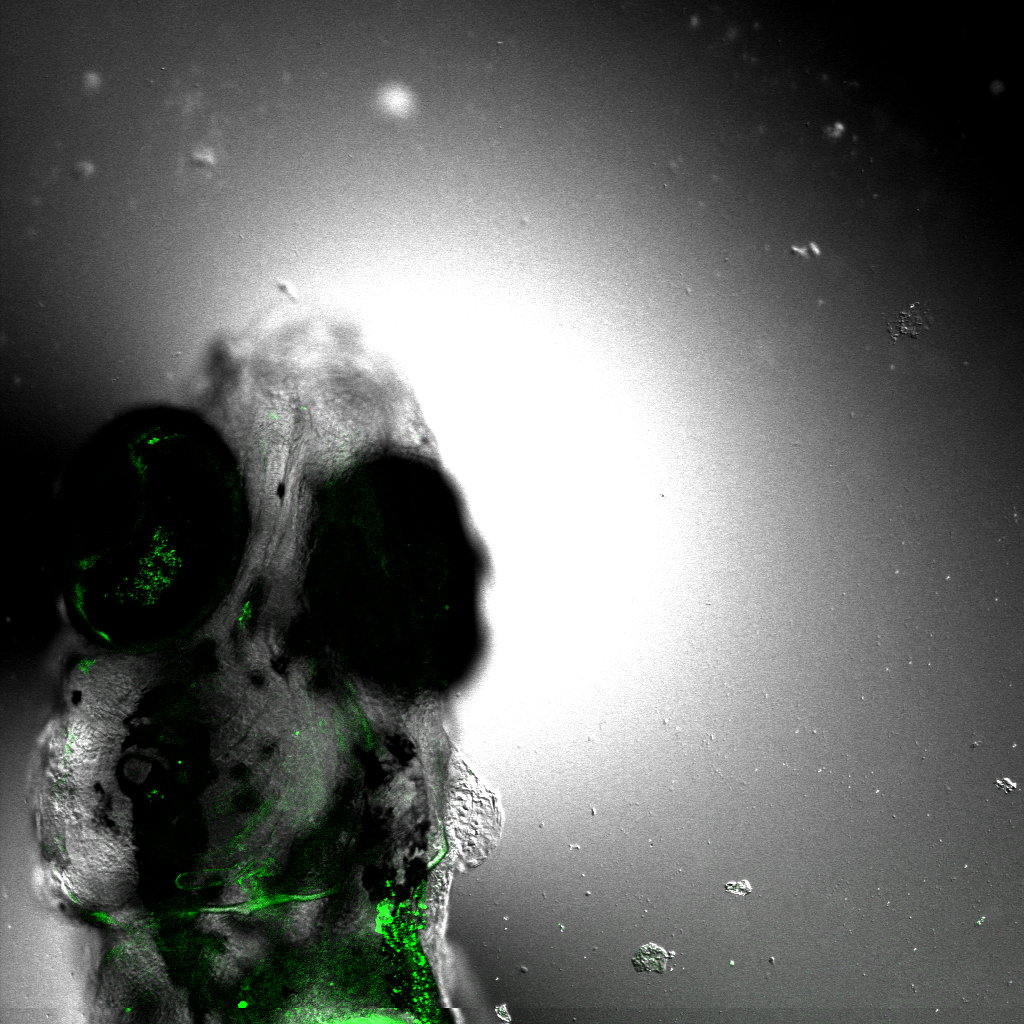

Supplement: Supplementary file 1 [file DataSheet3.zip › Original date(Figure 7-2)/Calcium ion/SCH23390+Sumanirole+Aconitine-(5).tif]

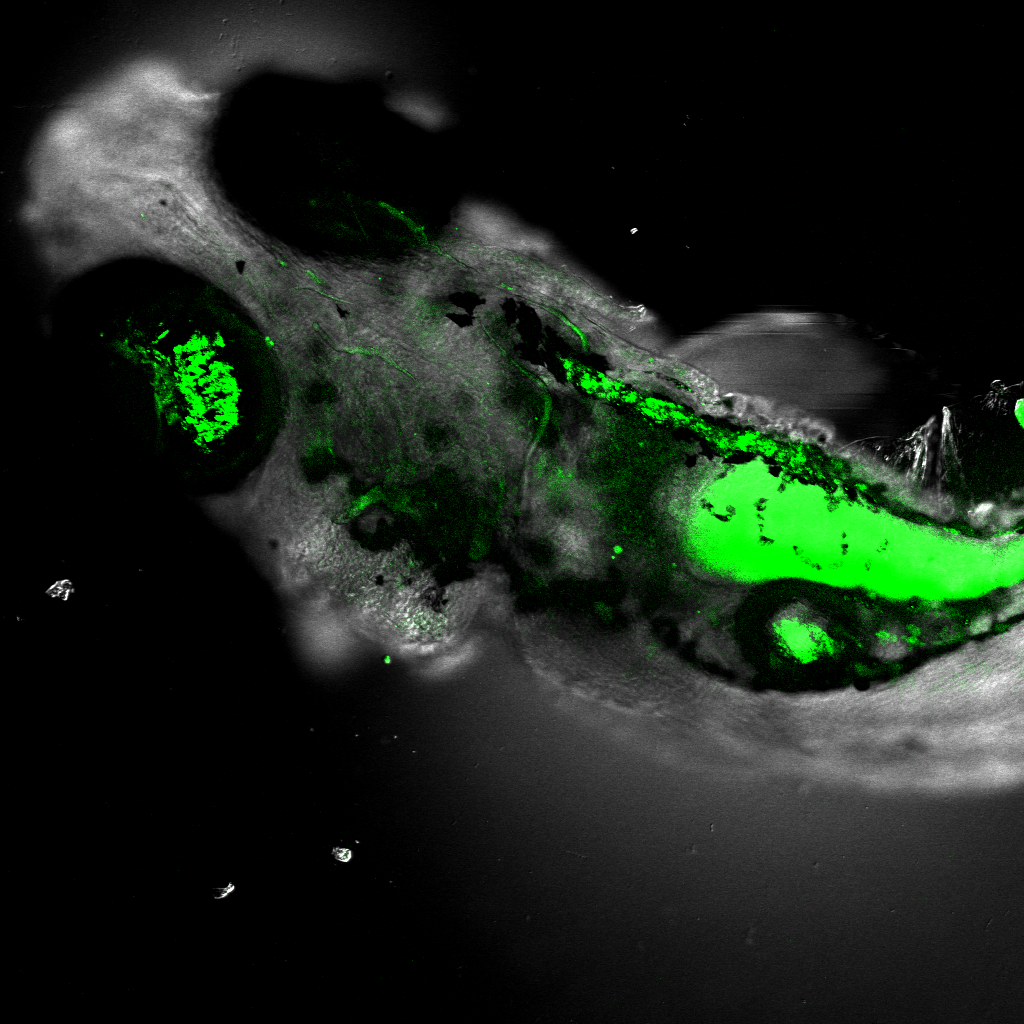

Supplement: Supplementary file 1 [file DataSheet3.zip › Original date(Figure 7-2)/Calcium ion/SCH23390+Sumanirole+Aconitine-(6).tif]

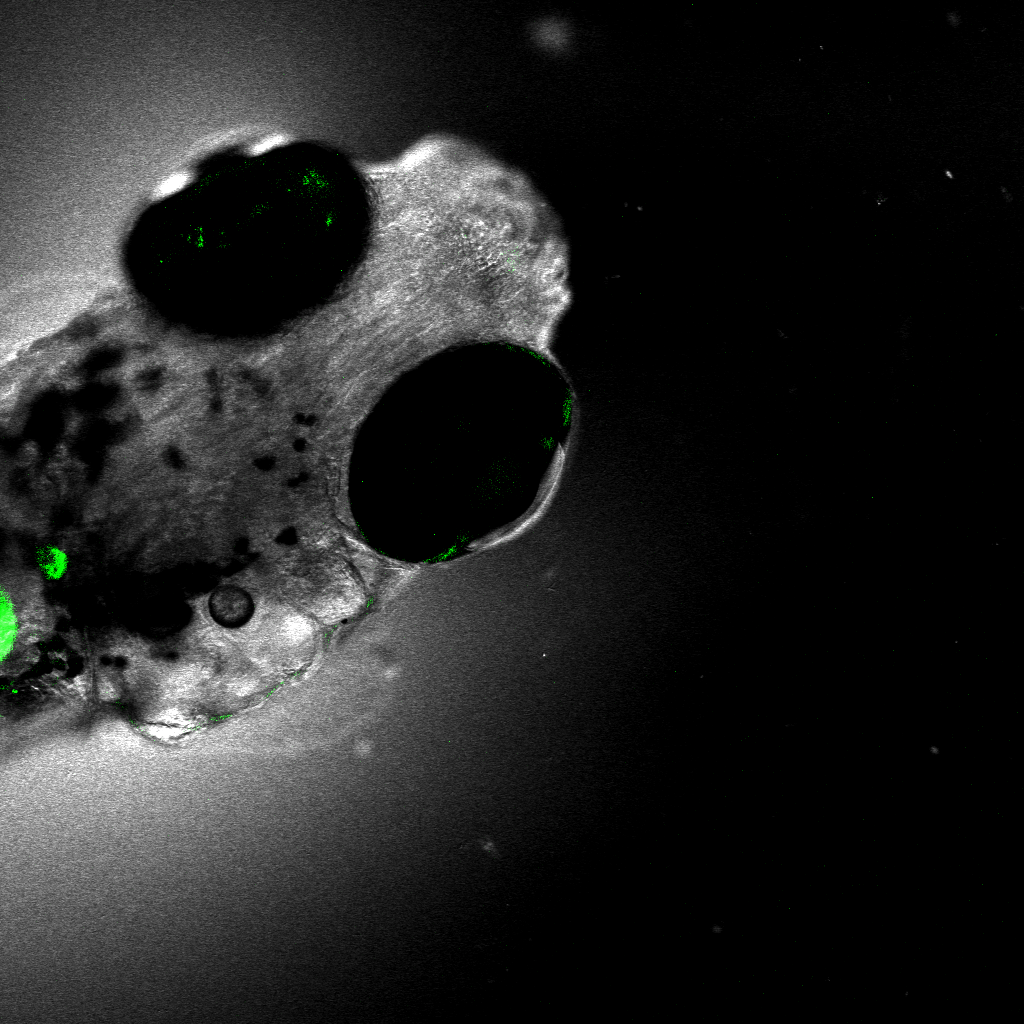

Supplement: Supplementary file 1 [file DataSheet3.zip › Original date(Figure 7-2)/Calcium ion/SCH23390+Sumanirole-(1).tif]

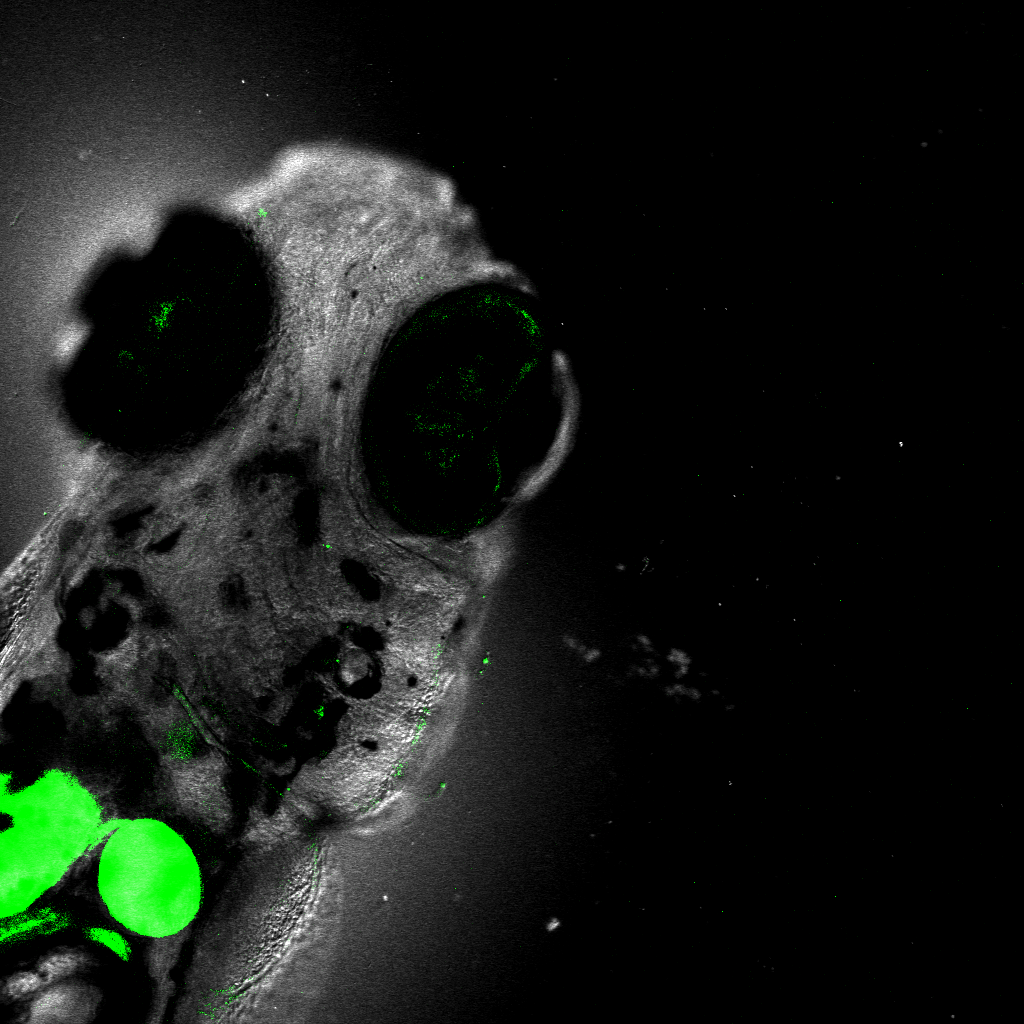

Supplement: Supplementary file 1 [file DataSheet3.zip › Original date(Figure 7-2)/Calcium ion/SCH23390+Sumanirole-(2).tif]

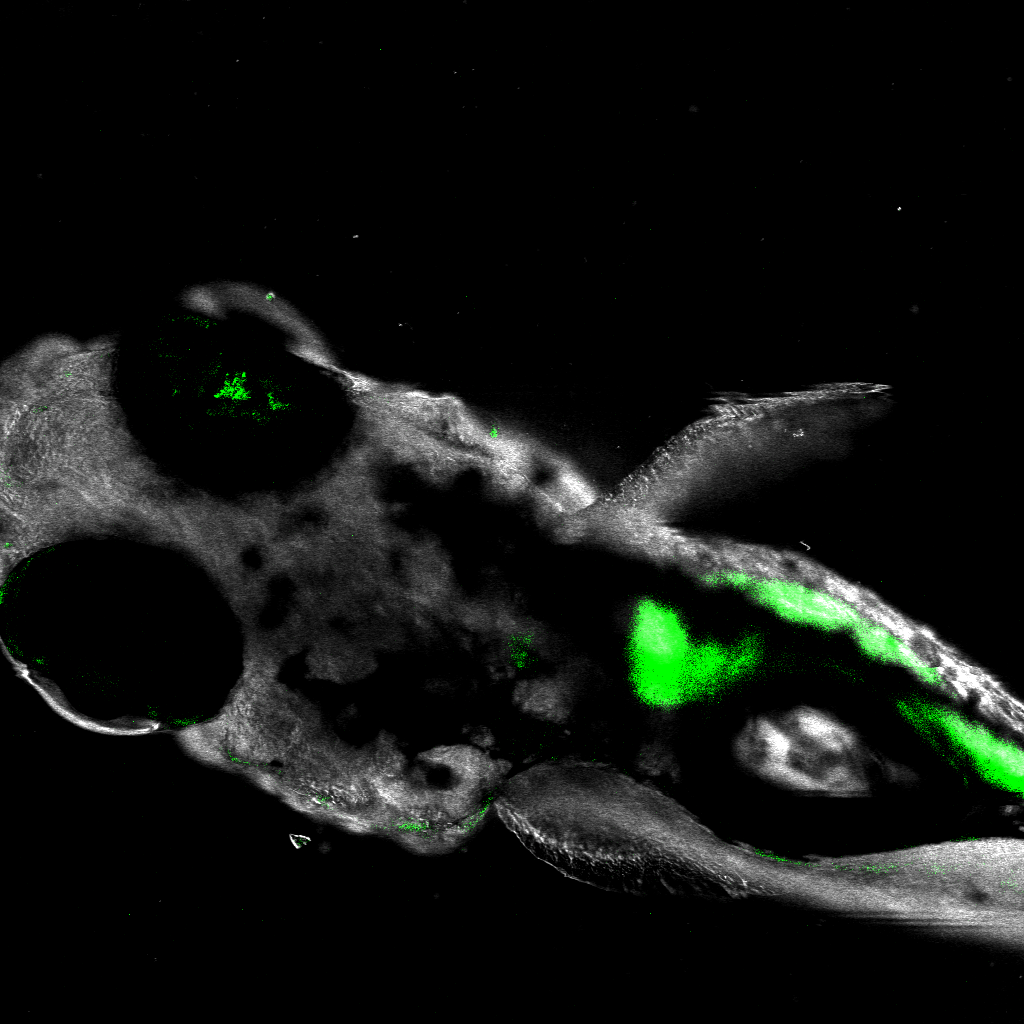

Supplement: Supplementary file 1 [file DataSheet3.zip › Original date(Figure 7-2)/Calcium ion/SCH23390+Sumanirole-(3).tif]

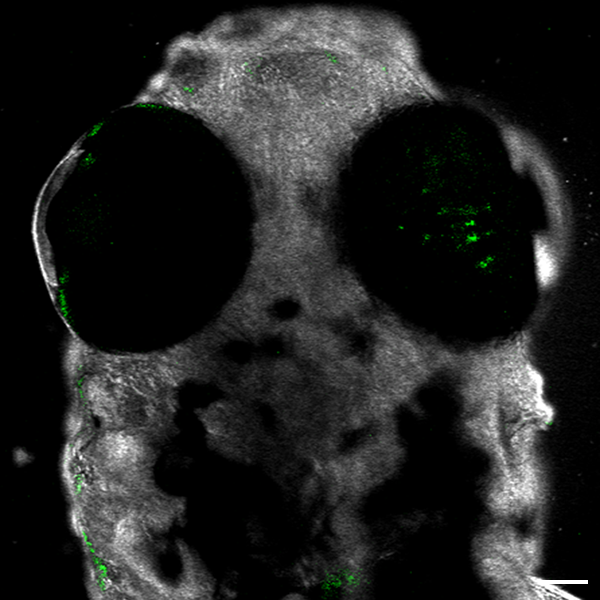

Supplement: Supplementary file 1 [file DataSheet3.zip › Original date(Figure 7-2)/Calcium ion/SCH23390+Sumanirole-(4).tif]

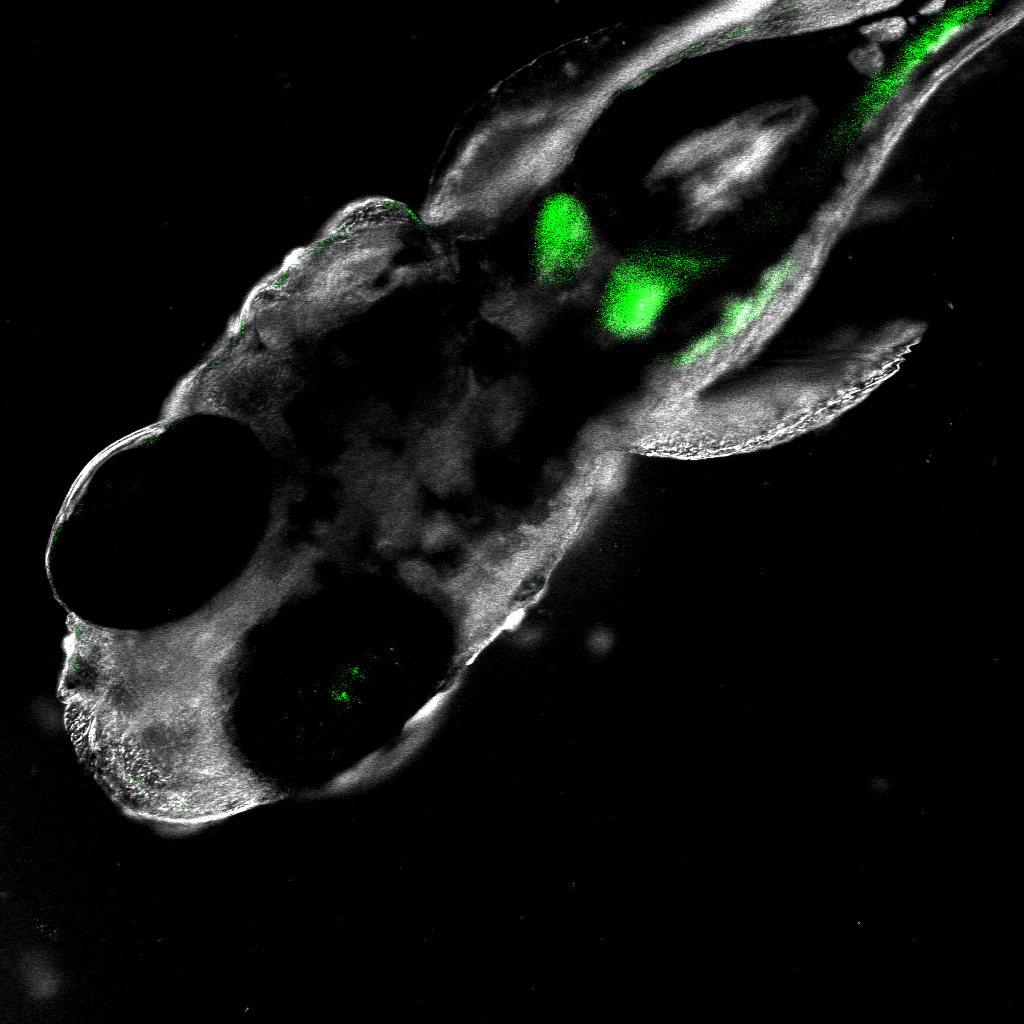

Supplement: Supplementary file 1 [file DataSheet3.zip › Original date(Figure 7-2)/Calcium ion/SCH23390+Sumanirole-(5).tif]

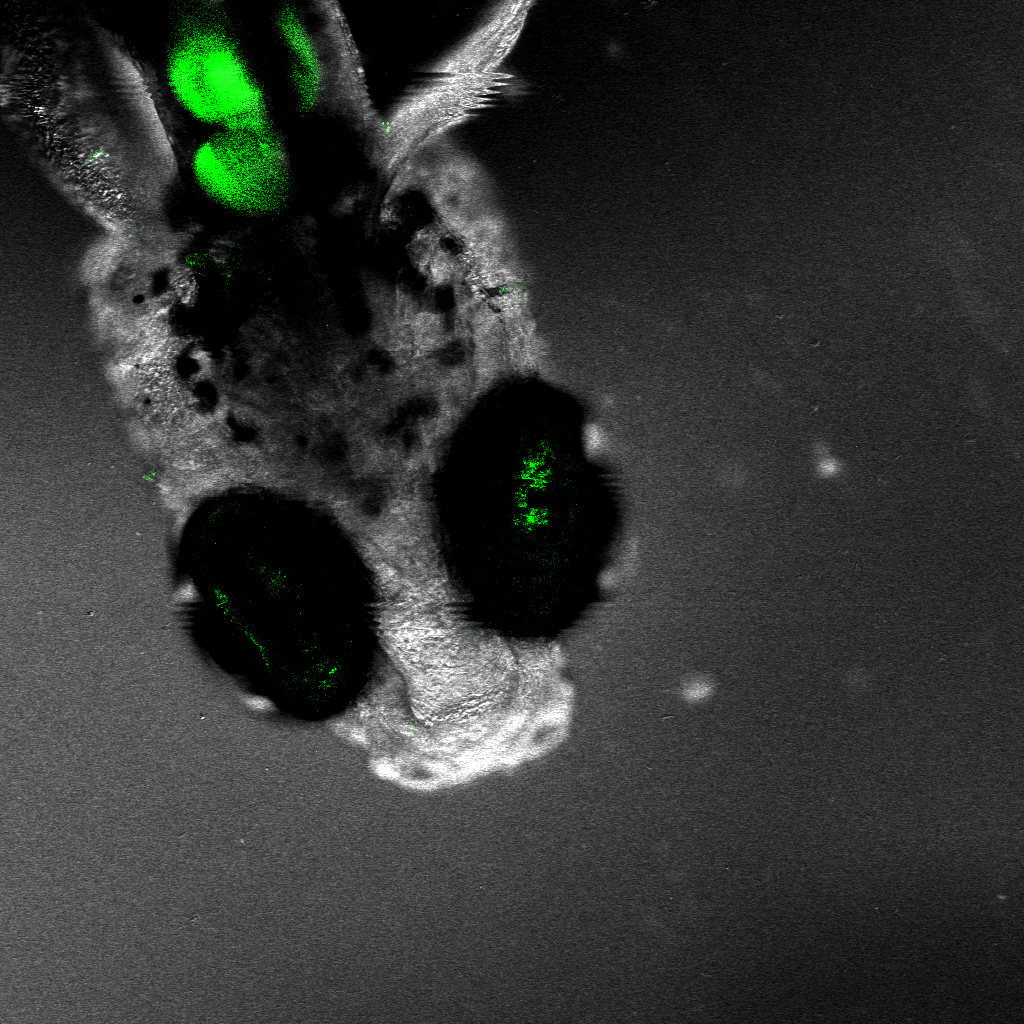

Supplement: Supplementary file 1 [file DataSheet3.zip › Original date(Figure 7-2)/Calcium ion/SCH23390+Sumanirole-(6).tif]

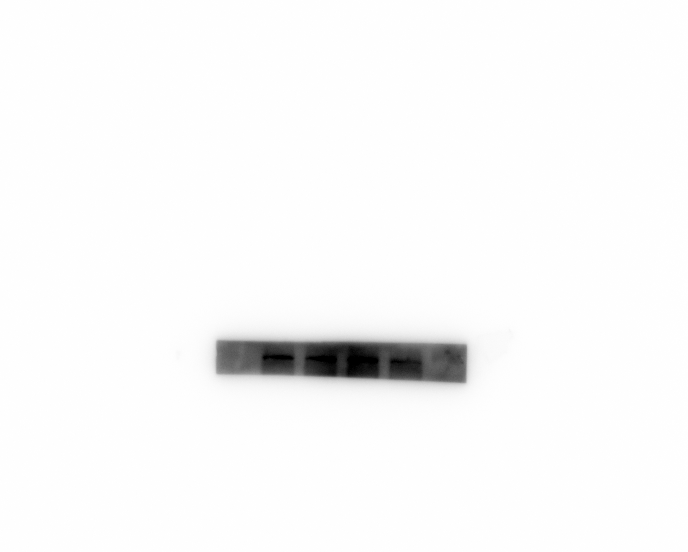

Supplement: Supplementary file 3 [file DataSheet4.zip › Original dataú¿Figure 8ú⌐/SCH23390-WB/ac-1-10s.tif]

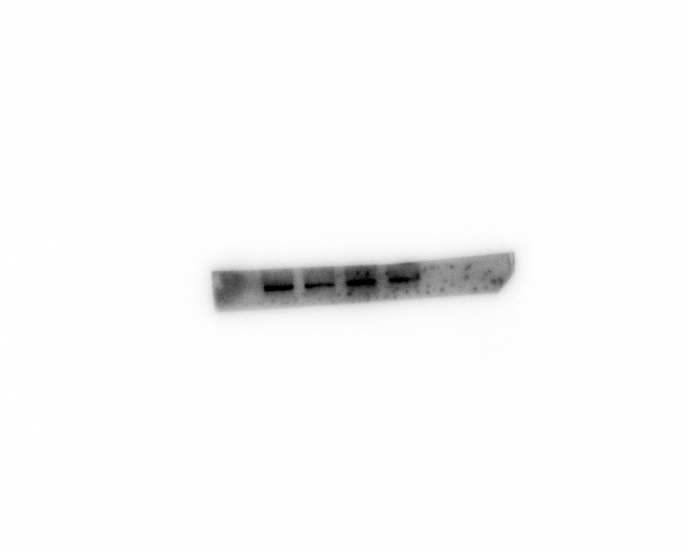

Supplement: Supplementary file 3 [file DataSheet4.zip › Original dataú¿Figure 8ú⌐/SCH23390-WB/ac-1-2-3s.tif]

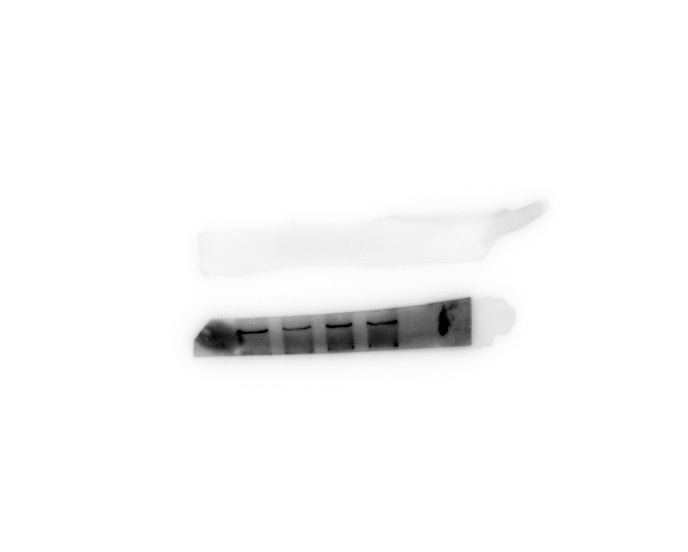

Supplement: Supplementary file 3 [file DataSheet4.zip › Original dataú¿Figure 8ú⌐/SCH23390-WB/ac-1-2.tif]

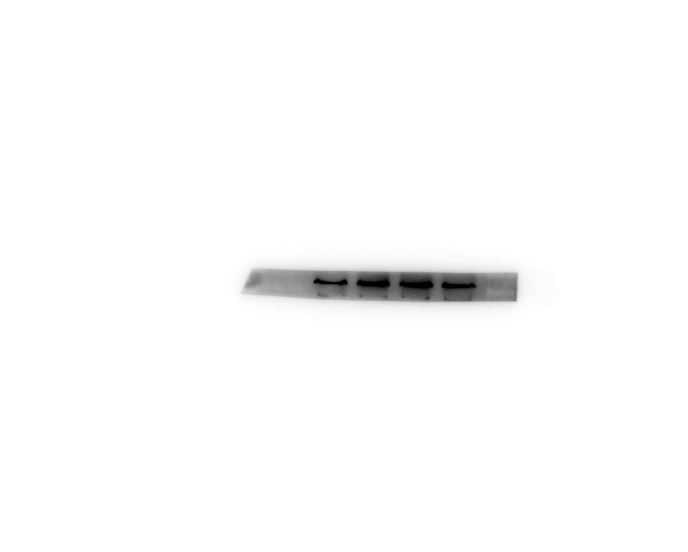

Supplement: Supplementary file 3 [file DataSheet4.zip › Original dataú¿Figure 8ú⌐/SCH23390-WB/ac-2-1-1.tif]

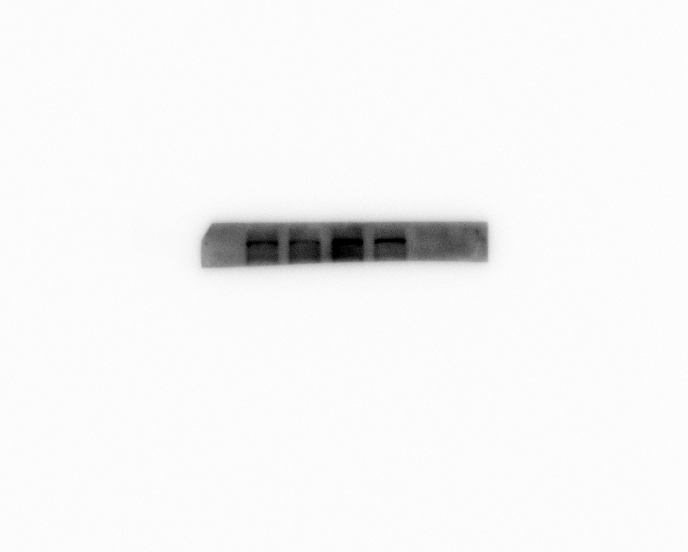

Supplement: Supplementary file 3 [file DataSheet4.zip › Original dataú¿Figure 8ú⌐/SCH23390-WB/ac-3-10s.tif]

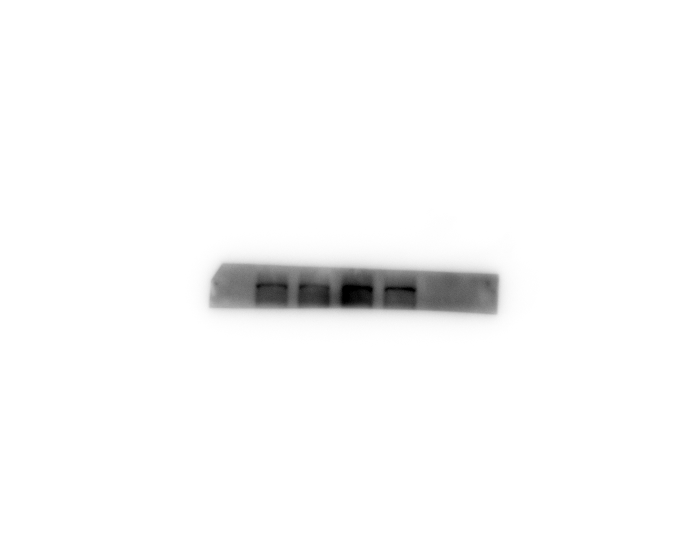

Supplement: Supplementary file 3 [file DataSheet4.zip › Original dataú¿Figure 8ú⌐/SCH23390-WB/ac-3-17s.tif]

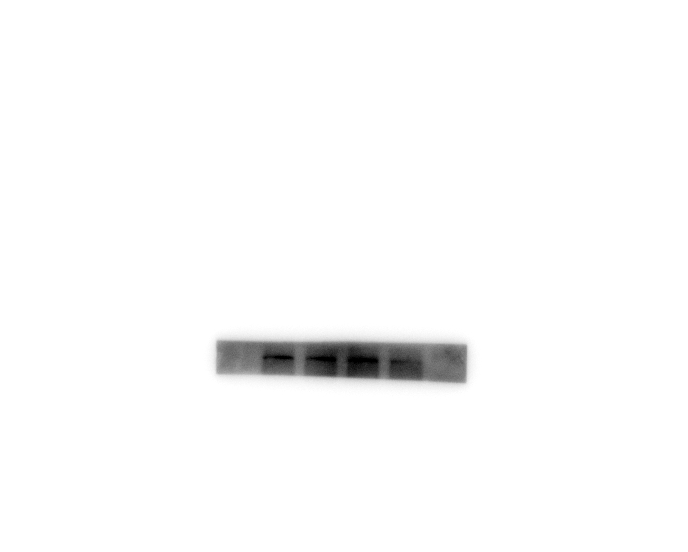

Supplement: Supplementary file 3 [file DataSheet4.zip › Original dataú¿Figure 8ú⌐/SCH23390-WB/ac-3s.tif]

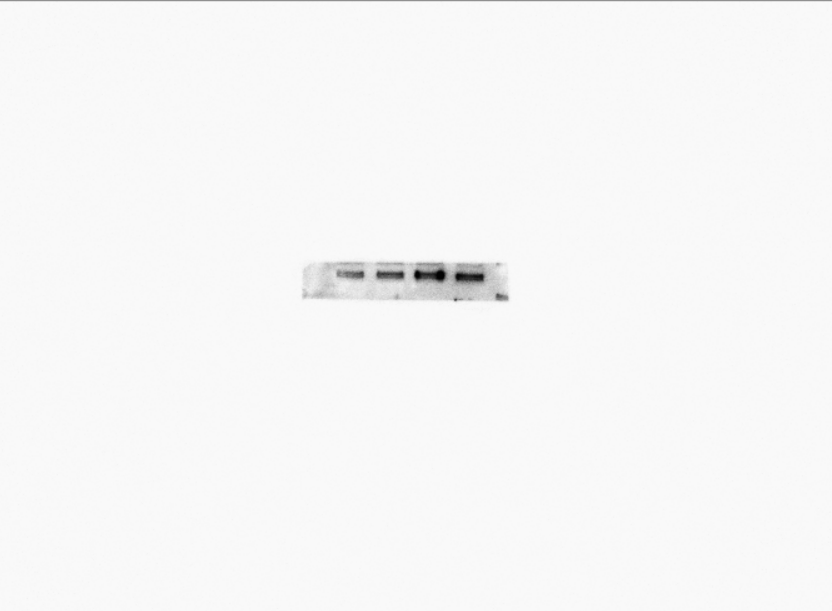

Supplement: Supplementary file 3 [file DataSheet4.zip › Original dataú¿Figure 8ú⌐/SCH23390-WB/D1.tif]

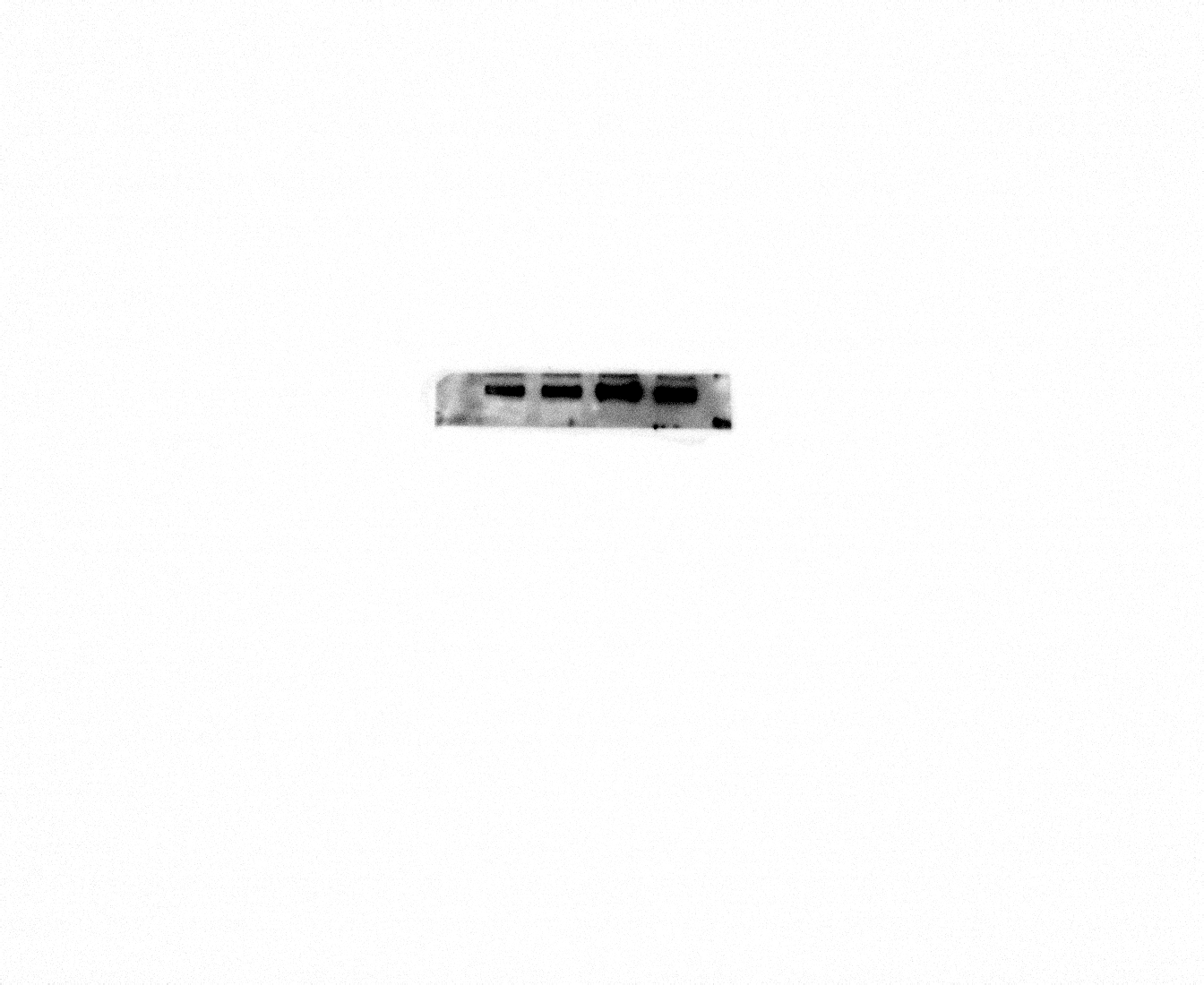

Supplement: Supplementary file 3 [file DataSheet4.zip › Original dataú¿Figure 8ú⌐/SCH23390-WB/d1_5].tif]

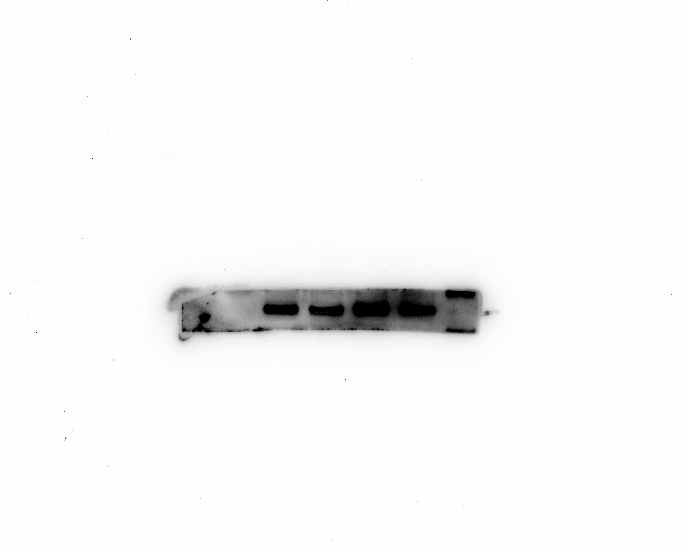

Supplement: Supplementary file 3 [file DataSheet4.zip › Original dataú¿Figure 8ú⌐/SCH23390-WB/p-n-2-d1-2.tif]

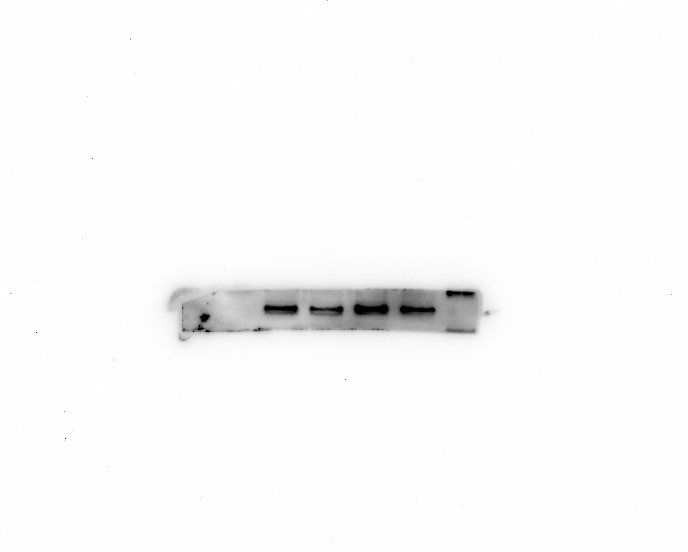

Supplement: Supplementary file 3 [file DataSheet4.zip › Original dataú¿Figure 8ú⌐/SCH23390-WB/p-n-2-d1-3.tif]

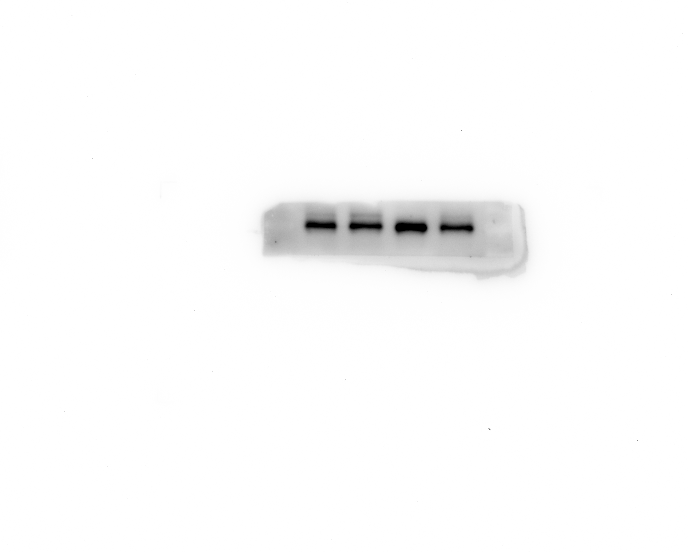

Supplement: Supplementary file 3 [file DataSheet4.zip › Original dataú¿Figure 8ú⌐/SCH23390-WB/p-pka-1.tif]

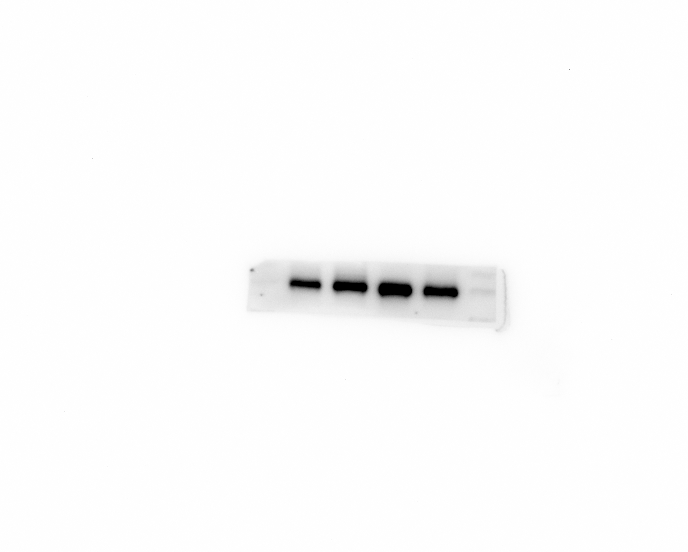

Supplement: Supplementary file 3 [file DataSheet4.zip › Original dataú¿Figure 8ú⌐/SCH23390-WB/p-pka-2.tif]

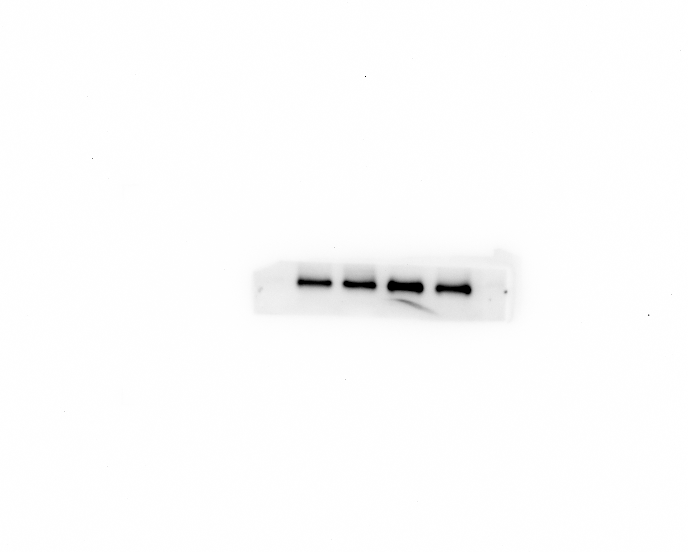

Supplement: Supplementary file 3 [file DataSheet4.zip › Original dataú¿Figure 8ú⌐/SCH23390-WB/p-pka-4.tif]

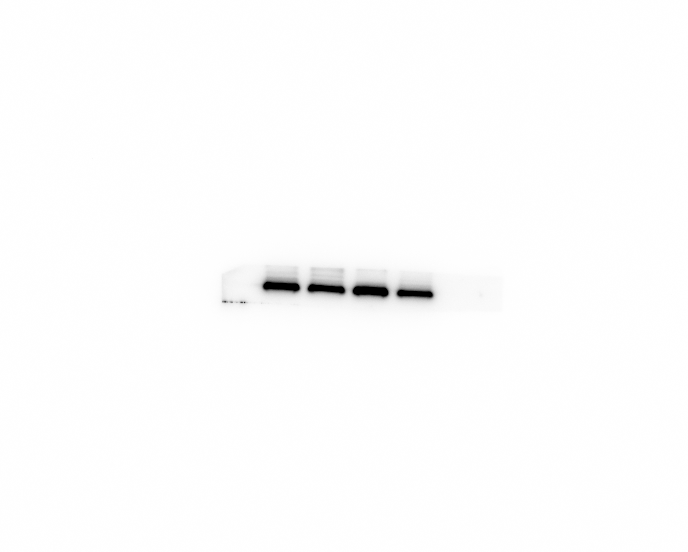

Supplement: Supplementary file 3 [file DataSheet4.zip › Original dataú¿Figure 8ú⌐/SCH23390-WB/p-pka-5.tif]

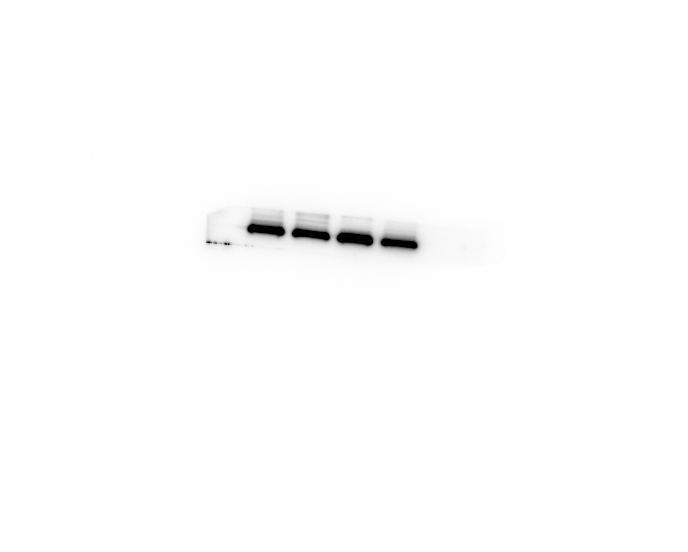

Supplement: Supplementary file 3 [file DataSheet4.zip › Original dataú¿Figure 8ú⌐/SCH23390-WB/p-pka-6.tif]

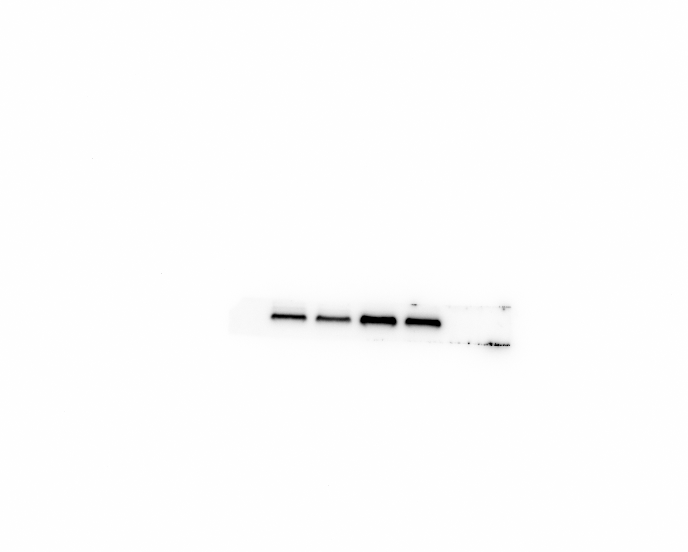

Supplement: Supplementary file 3 [file DataSheet4.zip › Original dataú¿Figure 8ú⌐/SCH23390-WB/p-pka-7.tif]

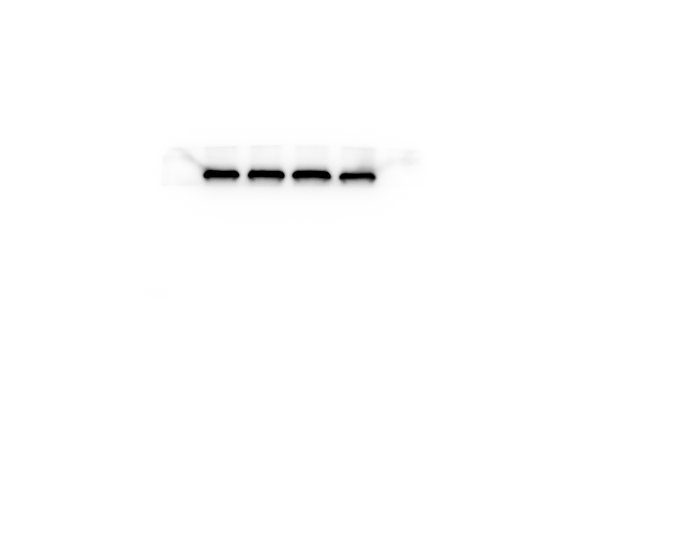

Supplement: Supplementary file 3 [file DataSheet4.zip › Original dataú¿Figure 8ú⌐/SCH23390-WB/p-pka-8.tif]

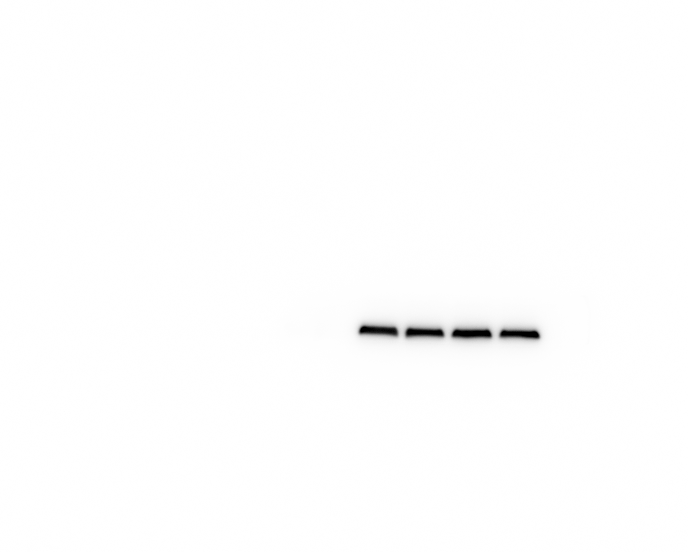

Supplement: Supplementary file 3 [file DataSheet4.zip › Original dataú¿Figure 8ú⌐/SCH23390-WB/pka-1.tif]

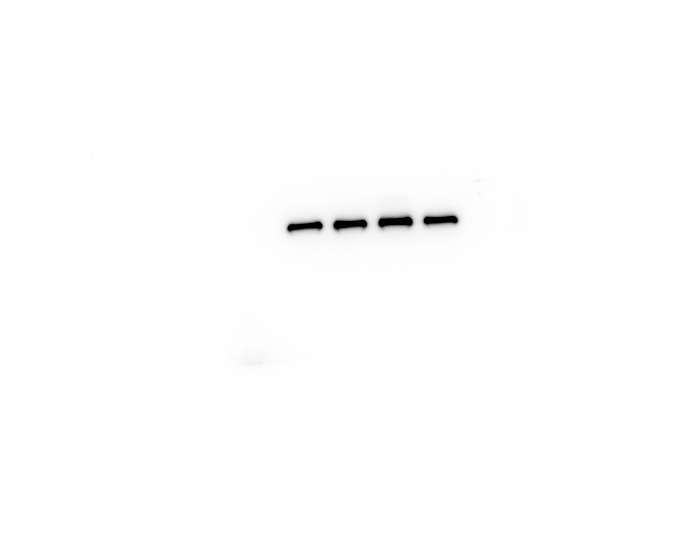

Supplement: Supplementary file 3 [file DataSheet4.zip › Original dataú¿Figure 8ú⌐/SCH23390-WB/pka-2.tif]

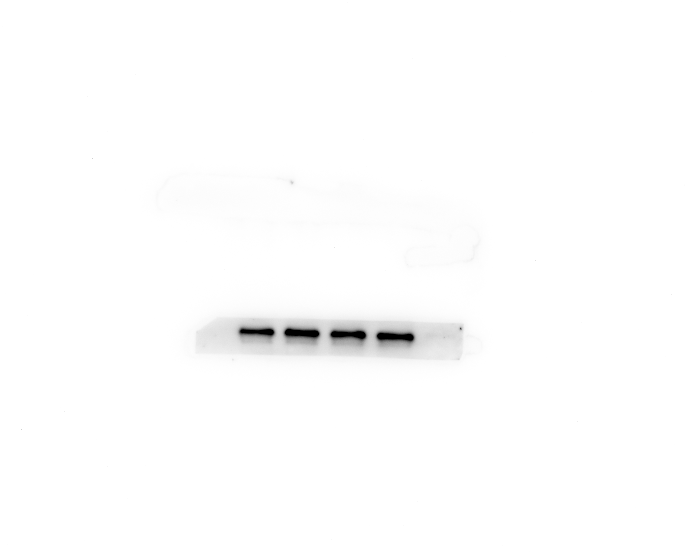

Supplement: Supplementary file 3 [file DataSheet4.zip › Original dataú¿Figure 8ú⌐/SCH23390-WB/pka-3.tif]

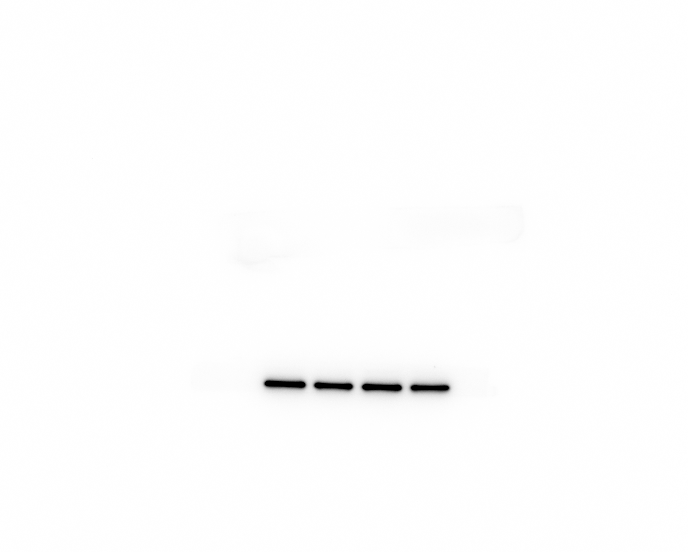

Supplement: Supplementary file 3 [file DataSheet4.zip › Original dataú¿Figure 8ú⌐/SCH23390-WB/pka-4.tif]

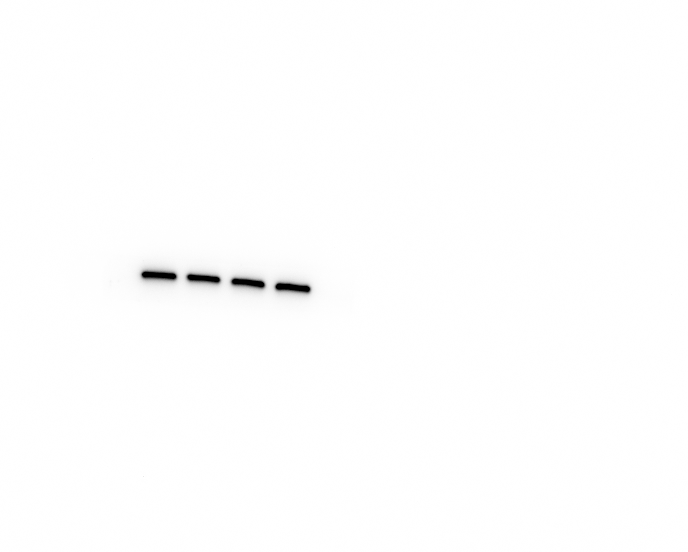

Supplement: Supplementary file 3 [file DataSheet4.zip › Original dataú¿Figure 8ú⌐/SCH23390-WB/pka-5.tif]

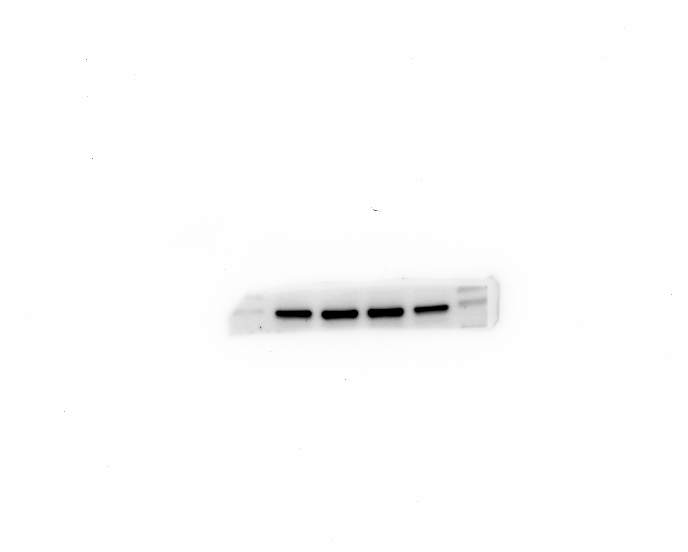

Supplement: Supplementary file 3 [file DataSheet4.zip › Original dataú¿Figure 8ú⌐/SCH23390-WB/pka-6.tif]

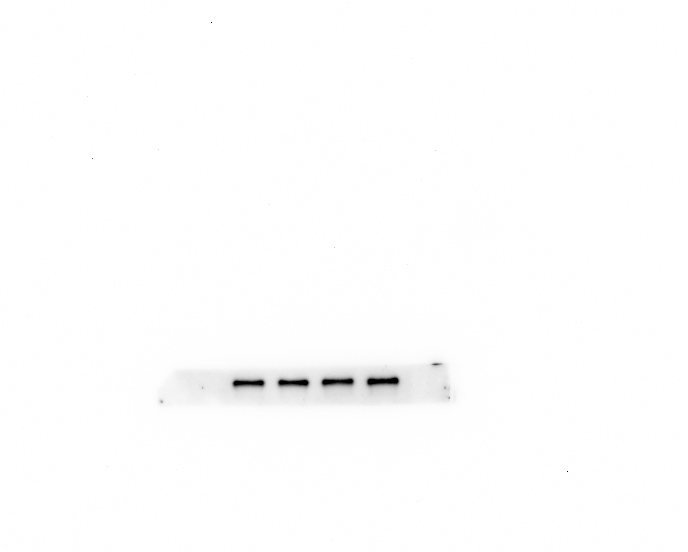

Supplement: Supplementary file 3 [file DataSheet4.zip › Original dataú¿Figure 8ú⌐/SCH23390-WB/pka-7.tif]

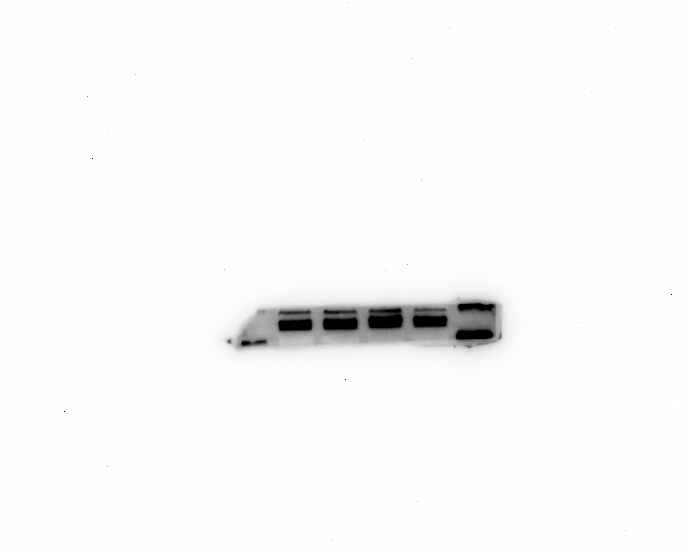

Supplement: Supplementary file 3 [file DataSheet4.zip › Original dataú¿Figure 8ú⌐/SCH23390-WB/sy-6-d1-2.tif]

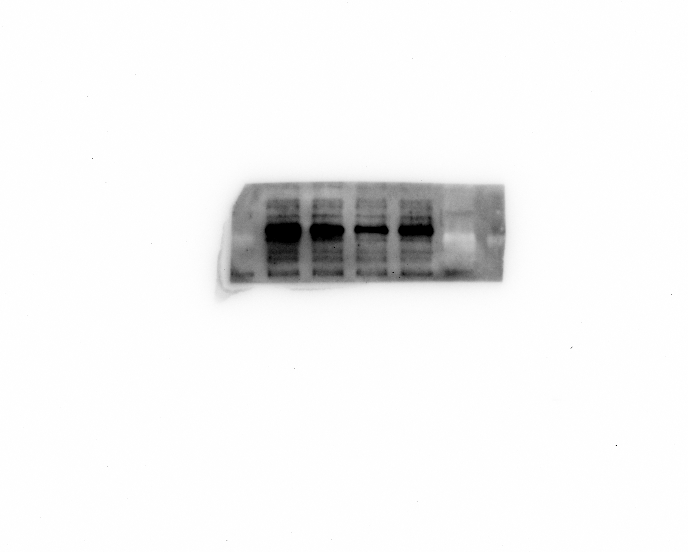

Supplement: Supplementary file 3 [file DataSheet4.zip › Original dataú¿Figure 8ú⌐/SCH23390-WB/sy-d2-4-1.tif]

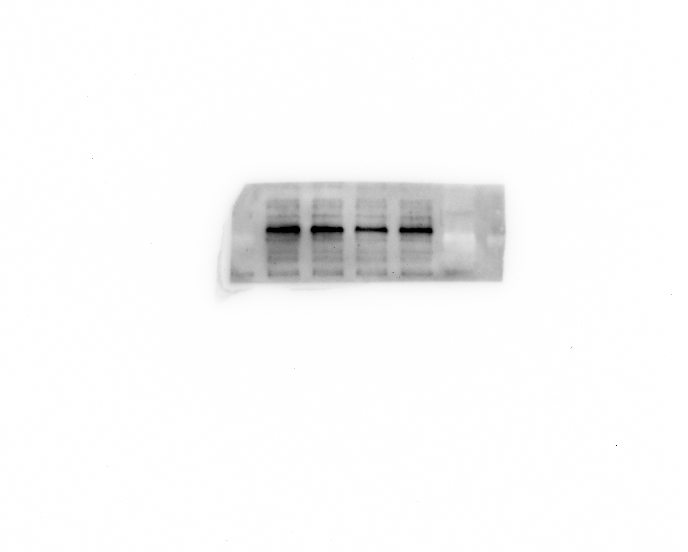

Supplement: Supplementary file 3 [file DataSheet4.zip › Original dataú¿Figure 8ú⌐/SCH23390-WB/sy-d2-4.tif]

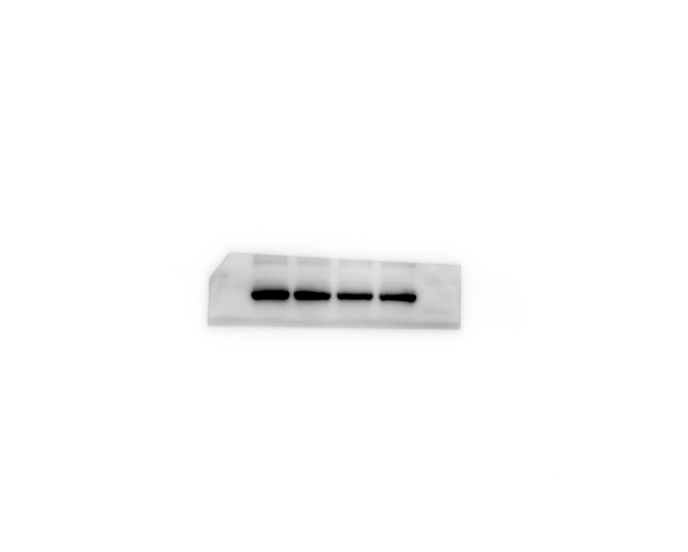

Supplement: Supplementary file 3 [file DataSheet4.zip › Original dataú¿Figure 8ú⌐/SCH23390-WB/SY-D2.tif]

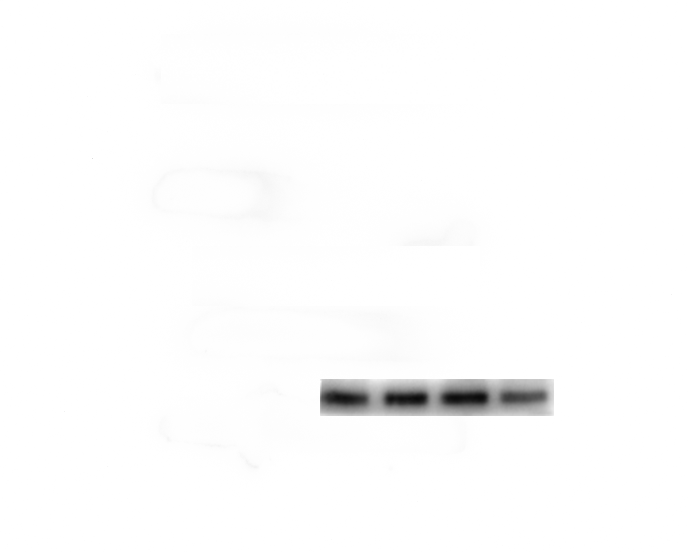

Supplement: Supplementary file 3 [file DataSheet4.zip › Original dataú¿Figure 8ú⌐/SCH23390-WB/═╝╞1⁄47-.tif]

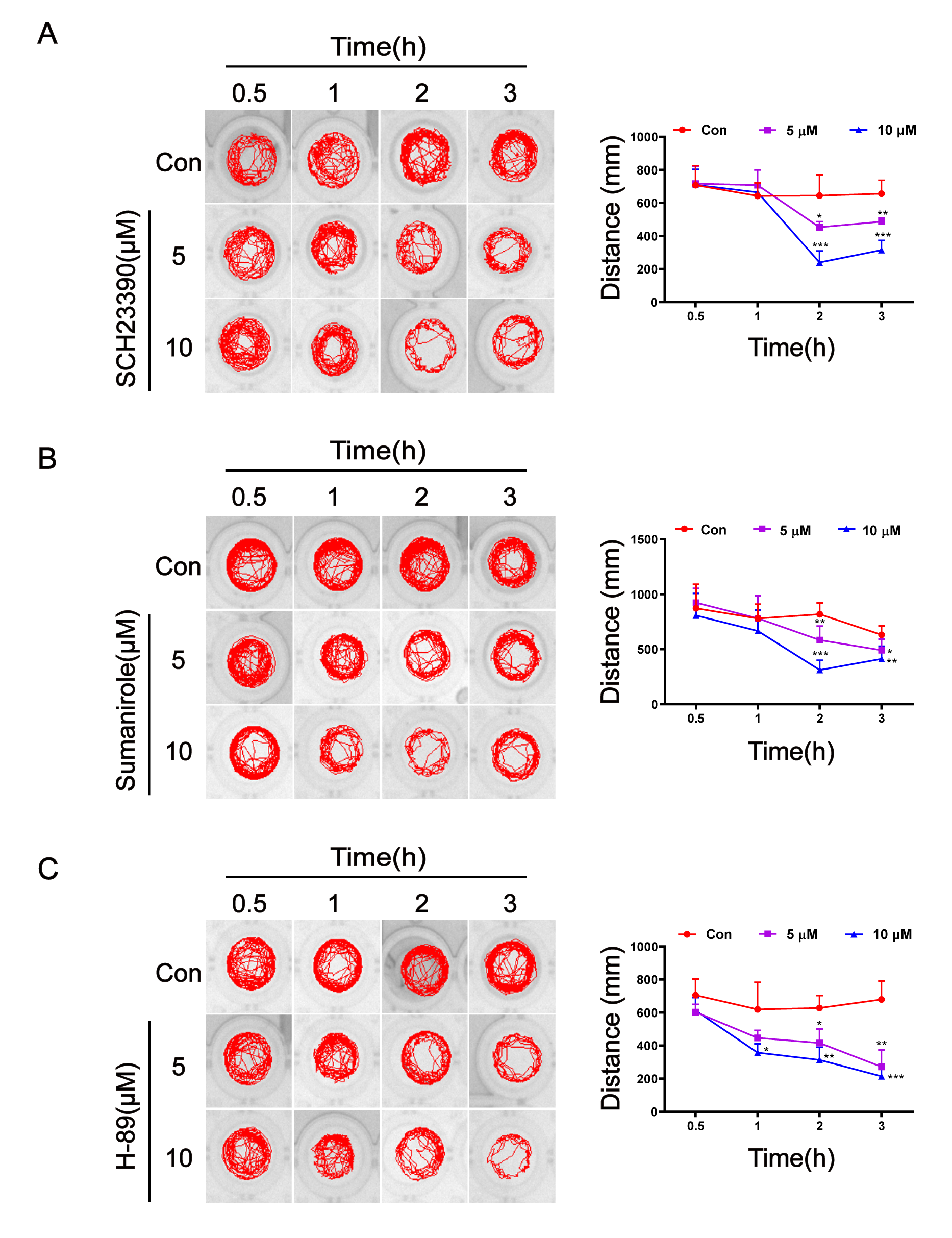

Supplement: Supplementary file 5 [file Image2.TIF]

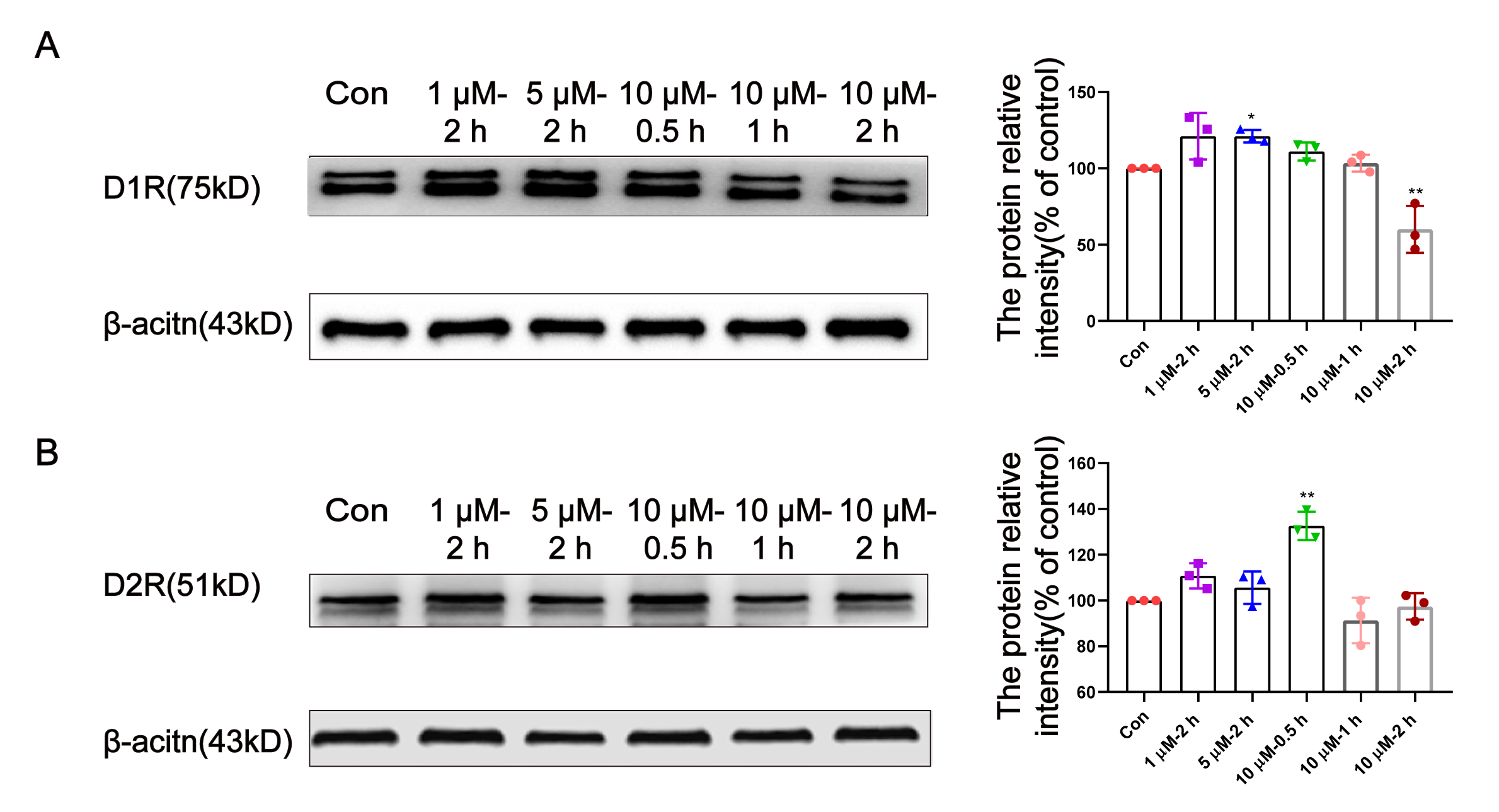

Supplement: Supplementary file 6 [file Image1.TIF]

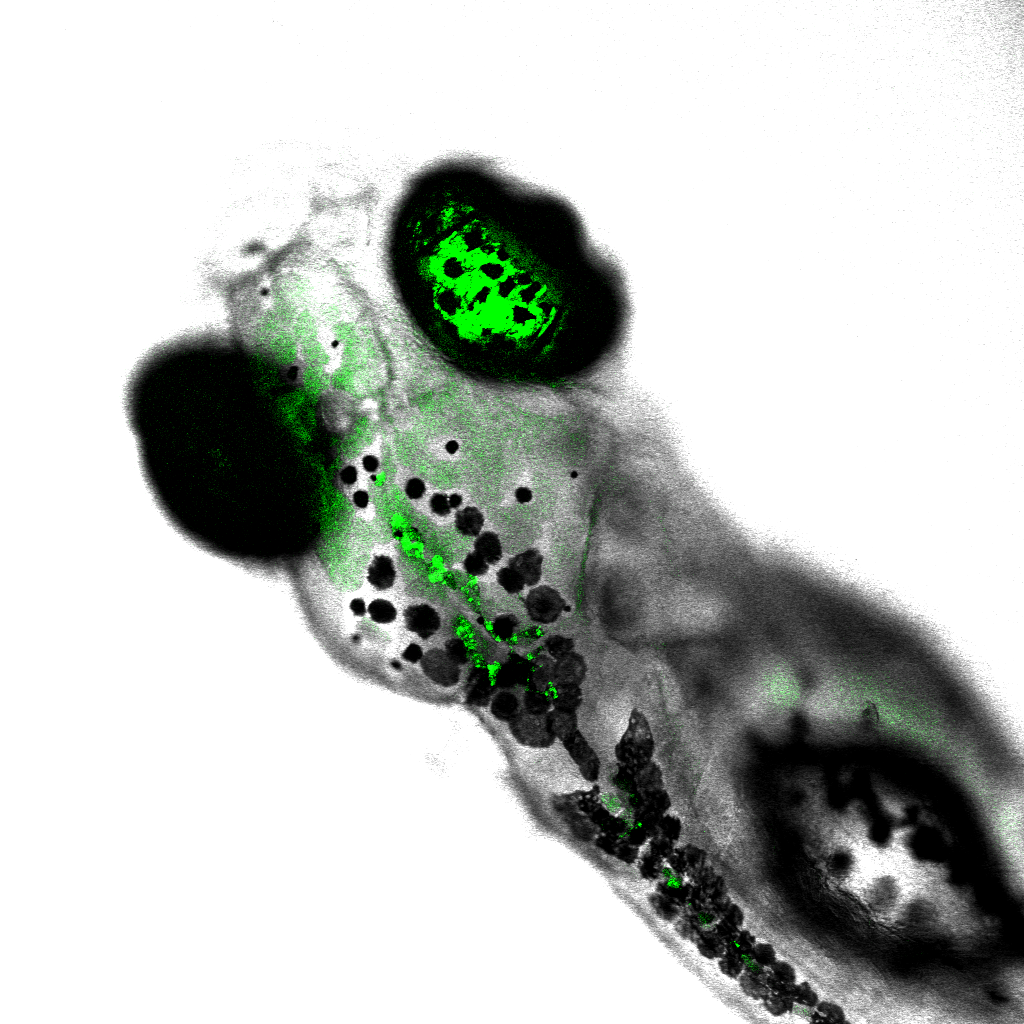

Supplement: Supplementary file 7 [file DataSheet2.zip › Original date(Figure 7-1)/SCH-Fluo 4-calcium ion/Aconitine-12_.tif]

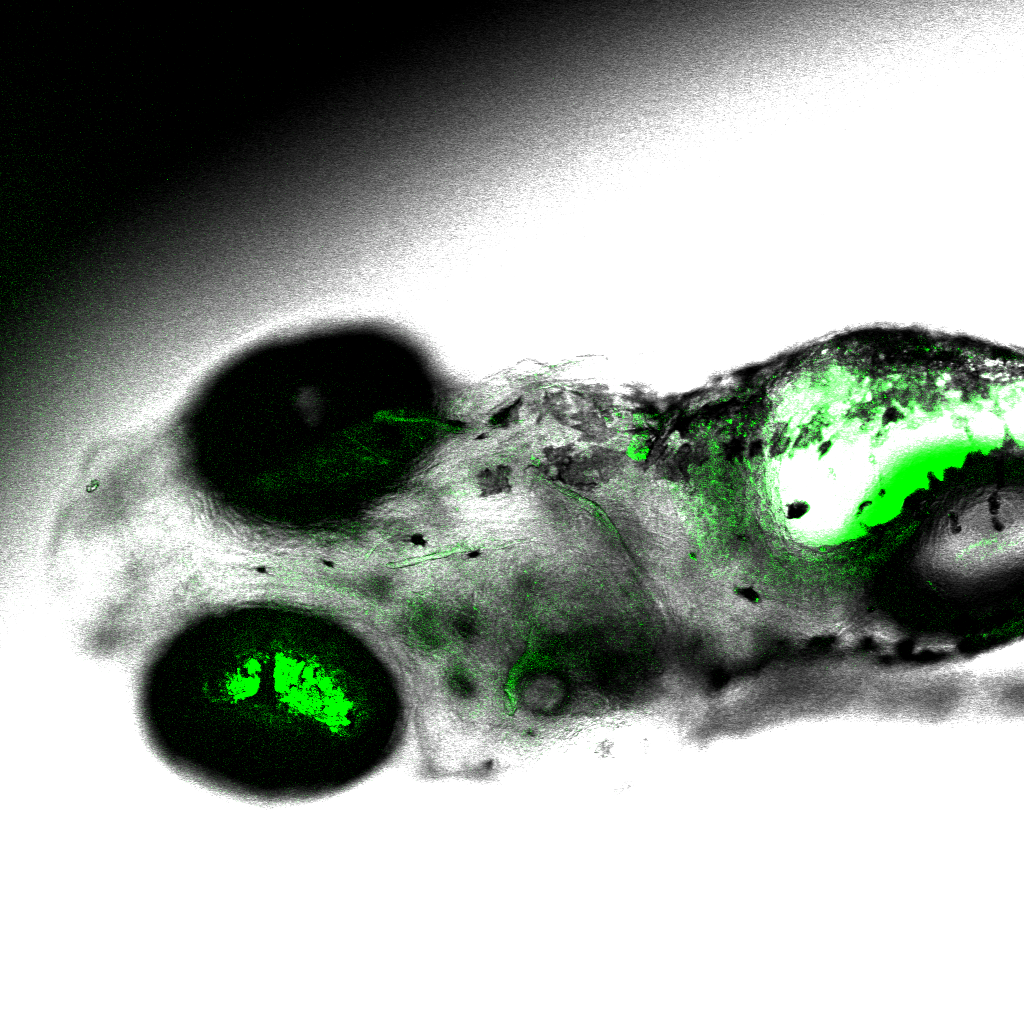

Supplement: Supplementary file 7 [file DataSheet2.zip › Original date(Figure 7-1)/SCH-Fluo 4-calcium ion/Aconitine-3_.tif]

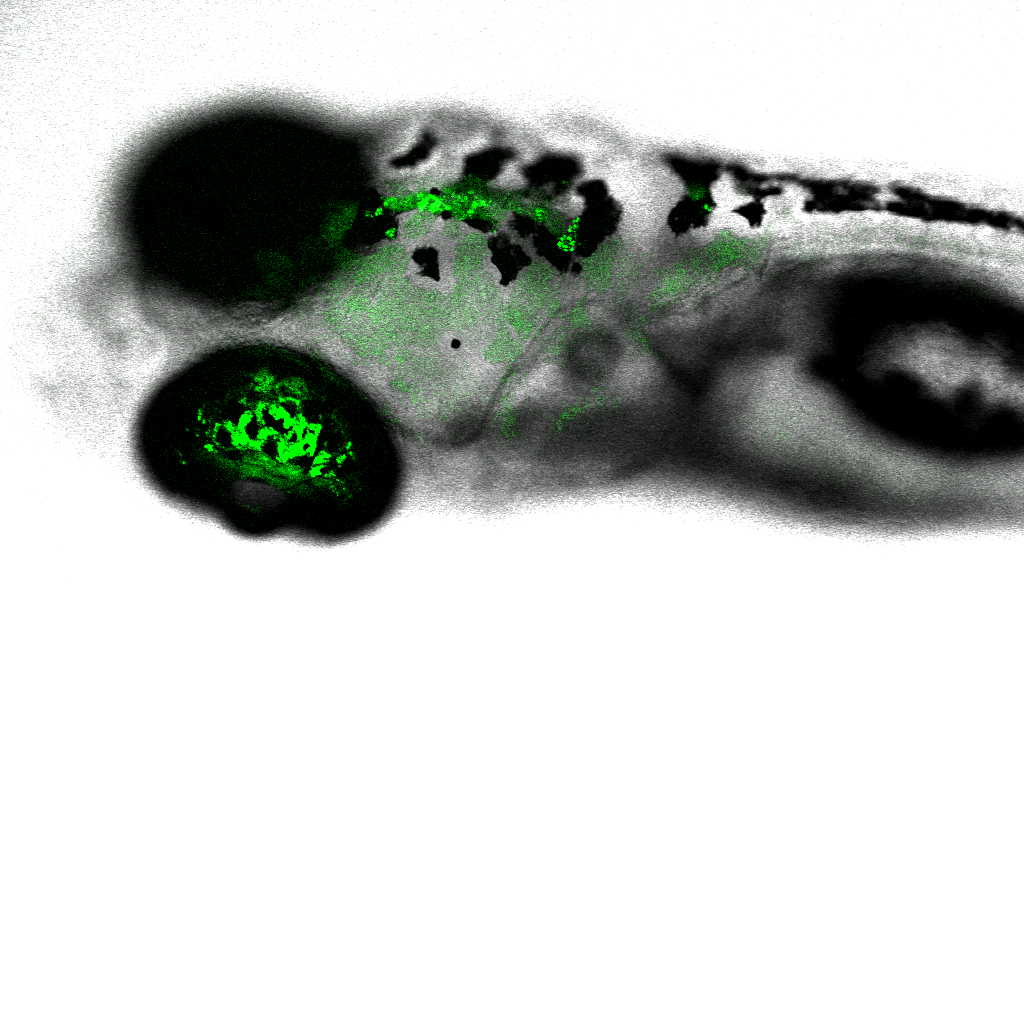

Supplement: Supplementary file 7 [file DataSheet2.zip › Original date(Figure 7-1)/SCH-Fluo 4-calcium ion/Aconitine-4_.tif]

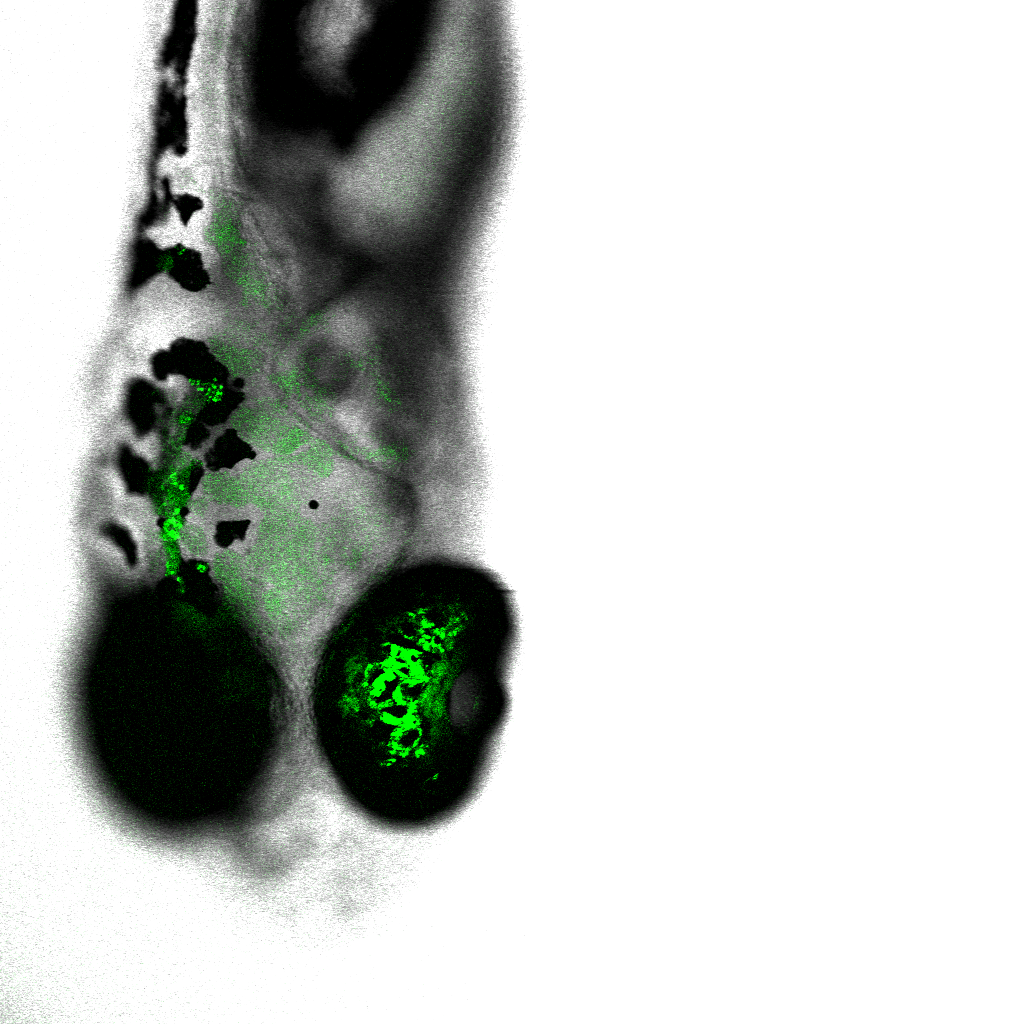

Supplement: Supplementary file 7 [file DataSheet2.zip › Original date(Figure 7-1)/SCH-Fluo 4-calcium ion/Aconitine-5_.tif]

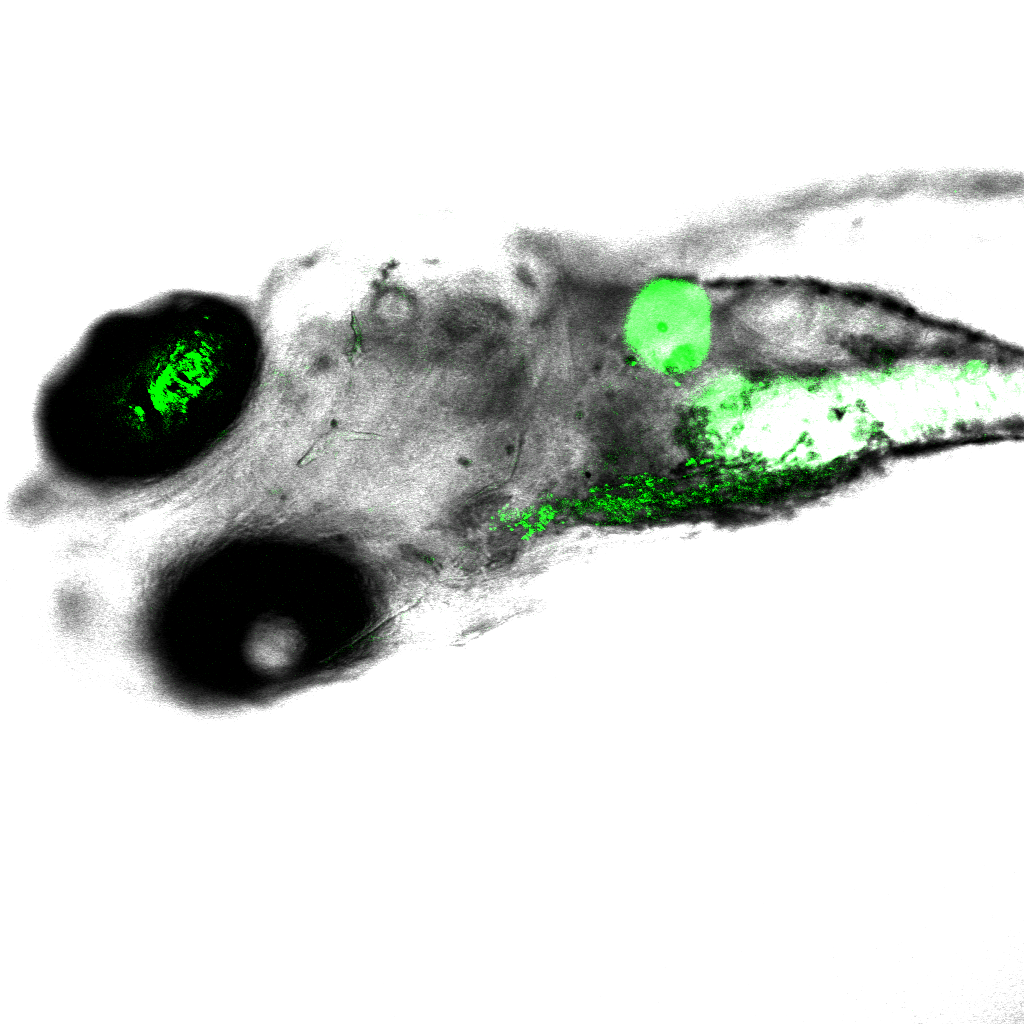

Supplement: Supplementary file 7 [file DataSheet2.zip › Original date(Figure 7-1)/SCH-Fluo 4-calcium ion/C-14_.tif]

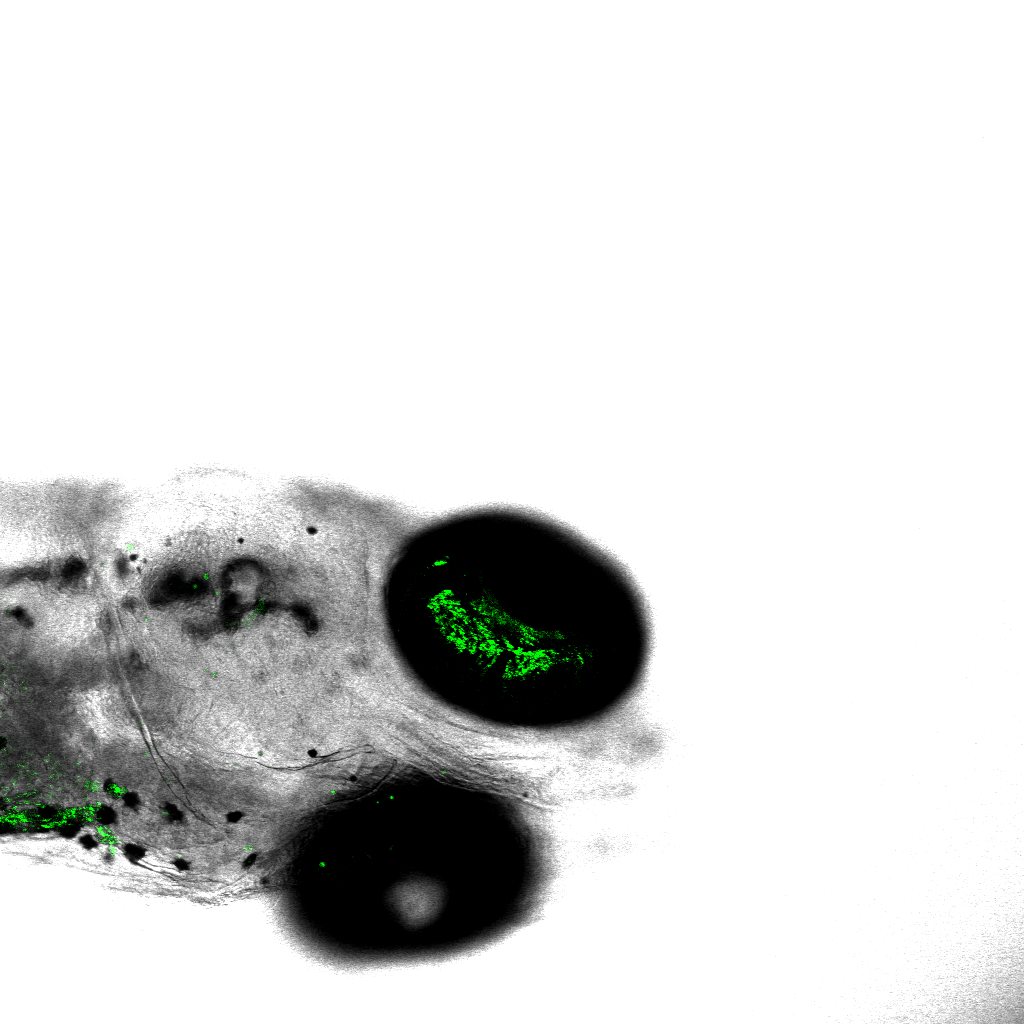

Supplement: Supplementary file 7 [file DataSheet2.zip › Original date(Figure 7-1)/SCH-Fluo 4-calcium ion/C-18_.tif]

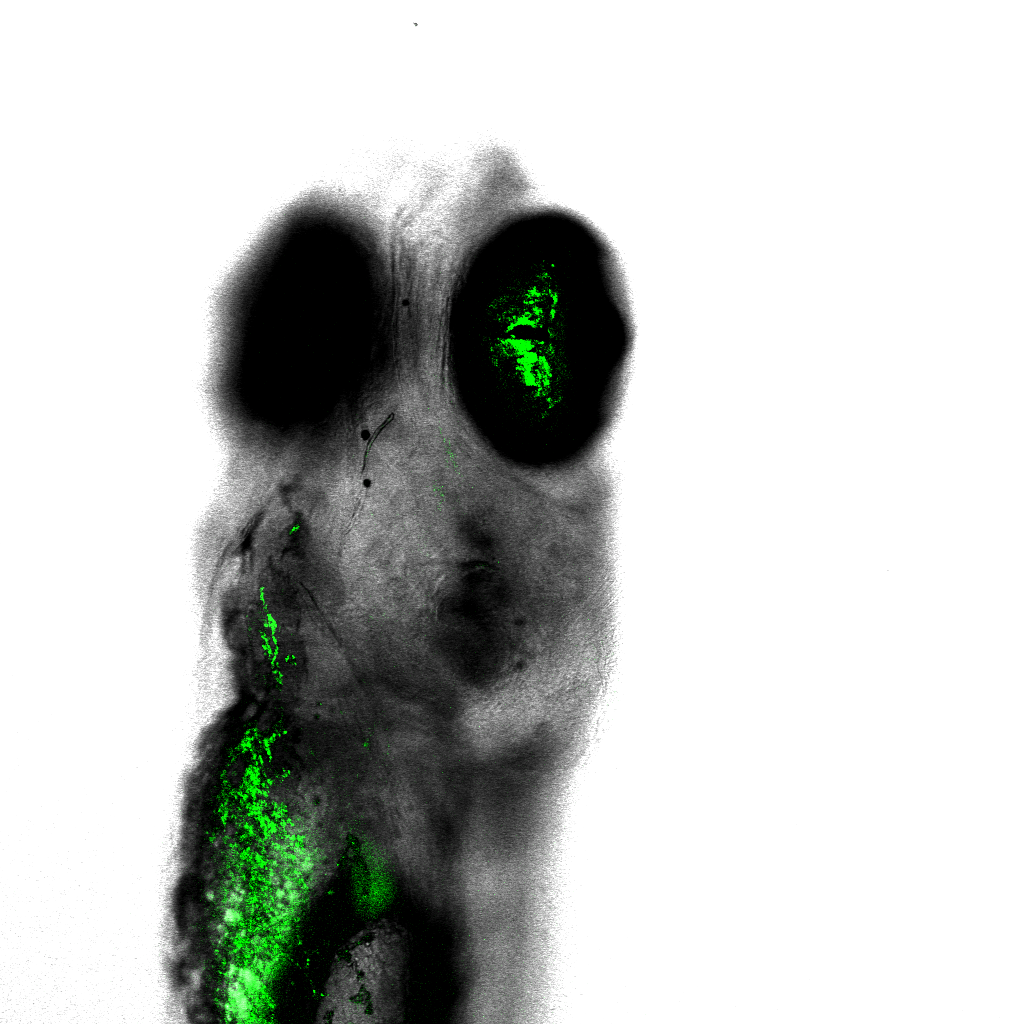

Supplement: Supplementary file 7 [file DataSheet2.zip › Original date(Figure 7-1)/SCH-Fluo 4-calcium ion/C-6_.tif]

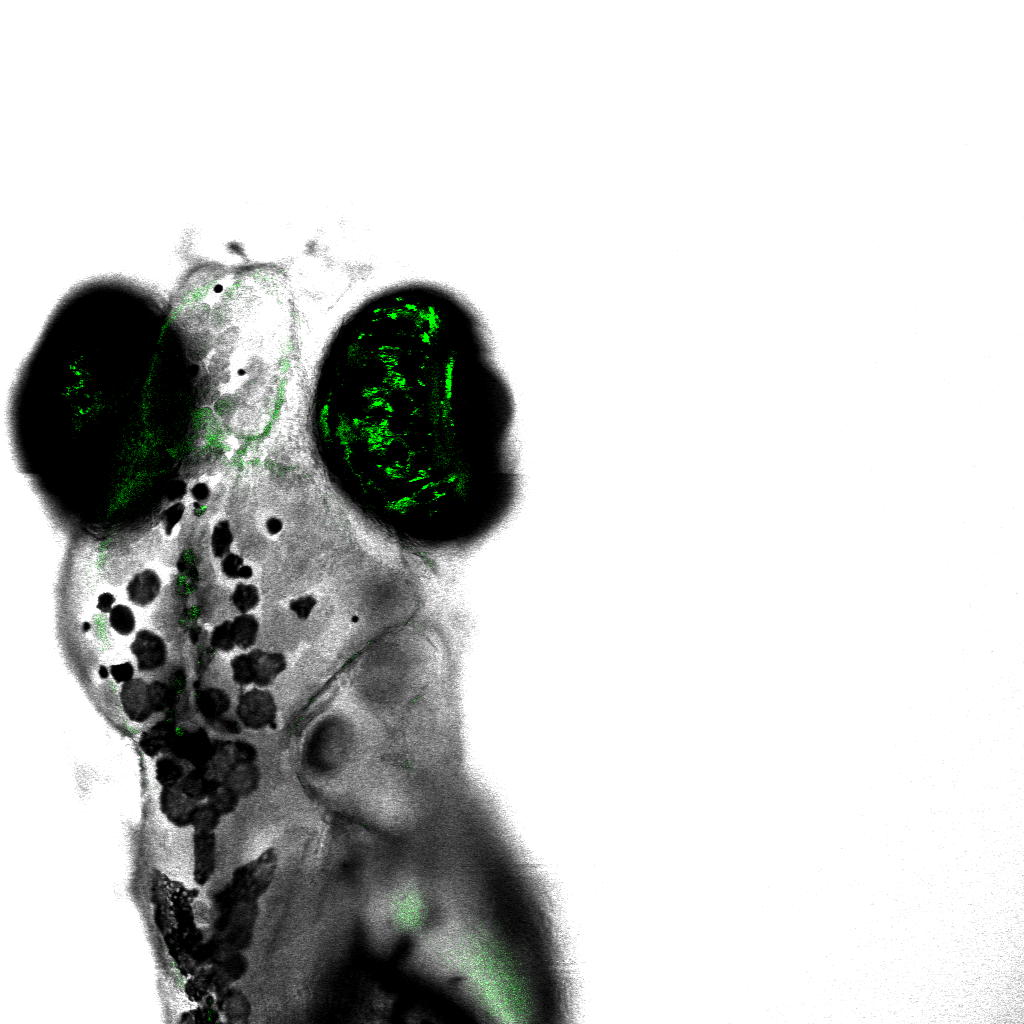

Supplement: Supplementary file 7 [file DataSheet2.zip › Original date(Figure 7-1)/SCH-Fluo 4-calcium ion/SCH23390+Aconitine-1_.tif]

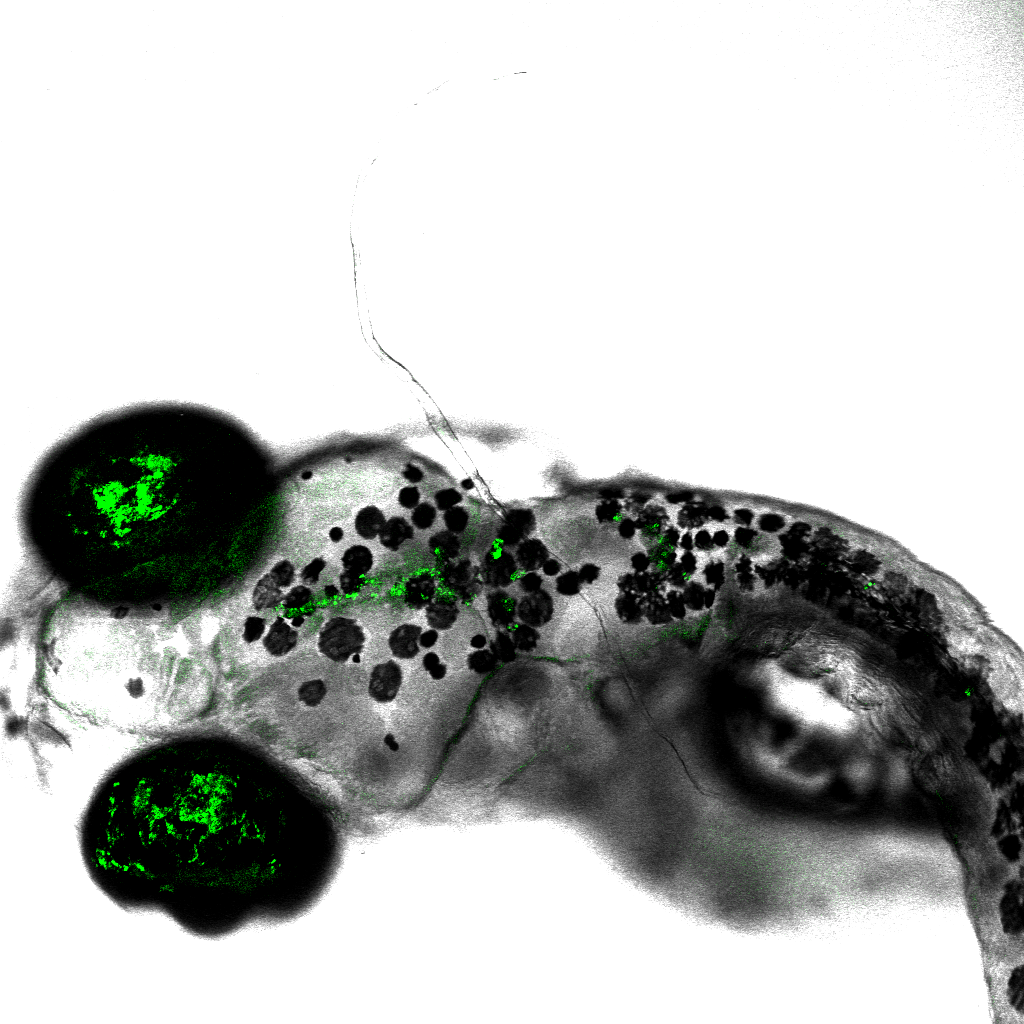

Supplement: Supplementary file 7 [file DataSheet2.zip › Original date(Figure 7-1)/SCH-Fluo 4-calcium ion/SCH23390+Aconitine-2_.tif]

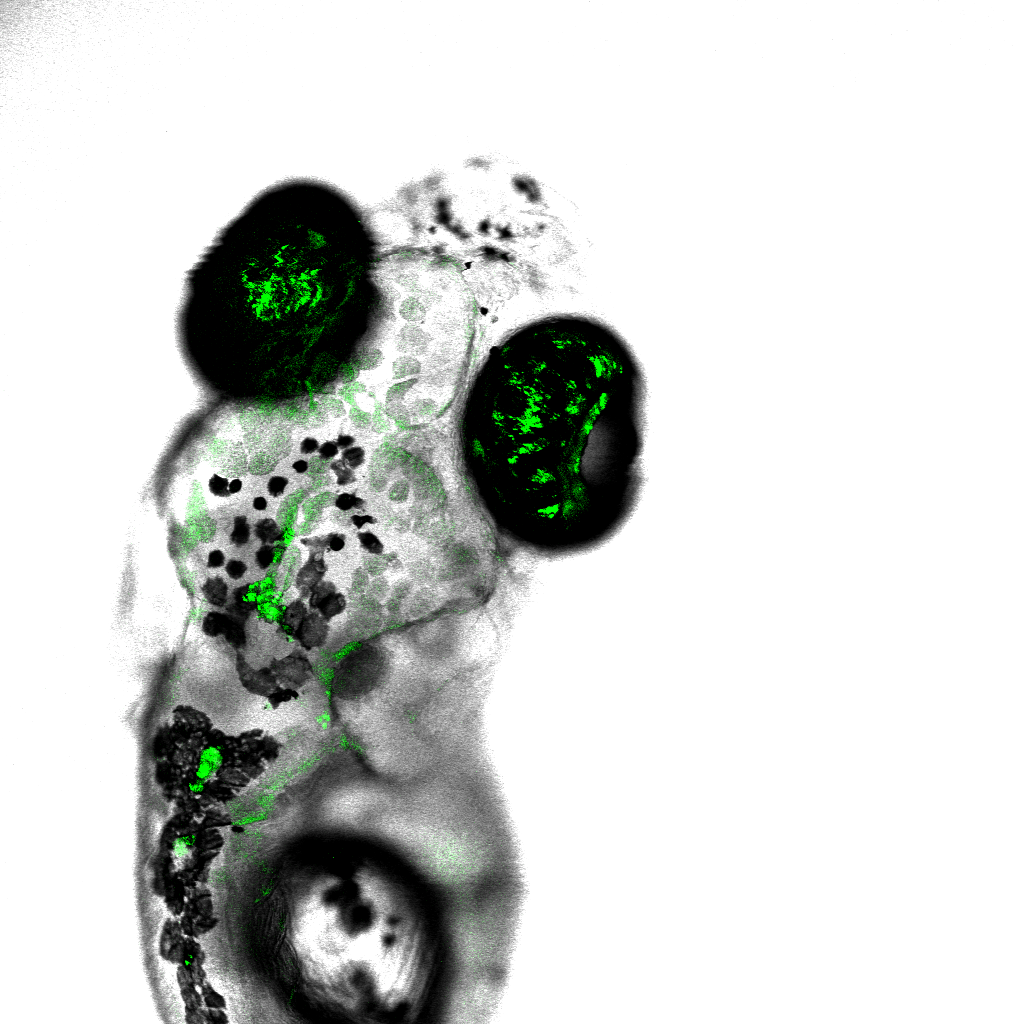

Supplement: Supplementary file 7 [file DataSheet2.zip › Original date(Figure 7-1)/SCH-Fluo 4-calcium ion/SCH23390+Aconitine-5_.tif]

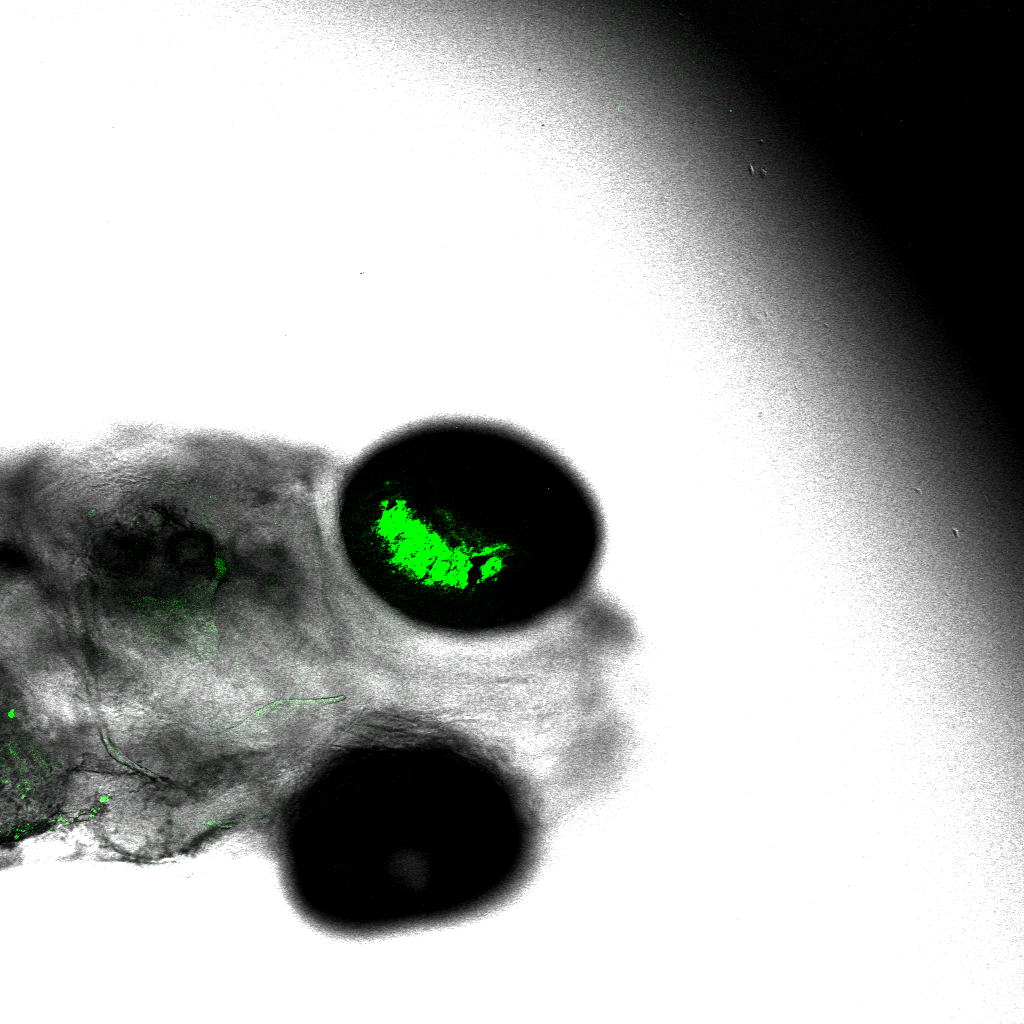

Supplement: Supplementary file 7 [file DataSheet2.zip › Original date(Figure 7-1)/SCH-Fluo 4-calcium ion/SCH23390-1.tif]

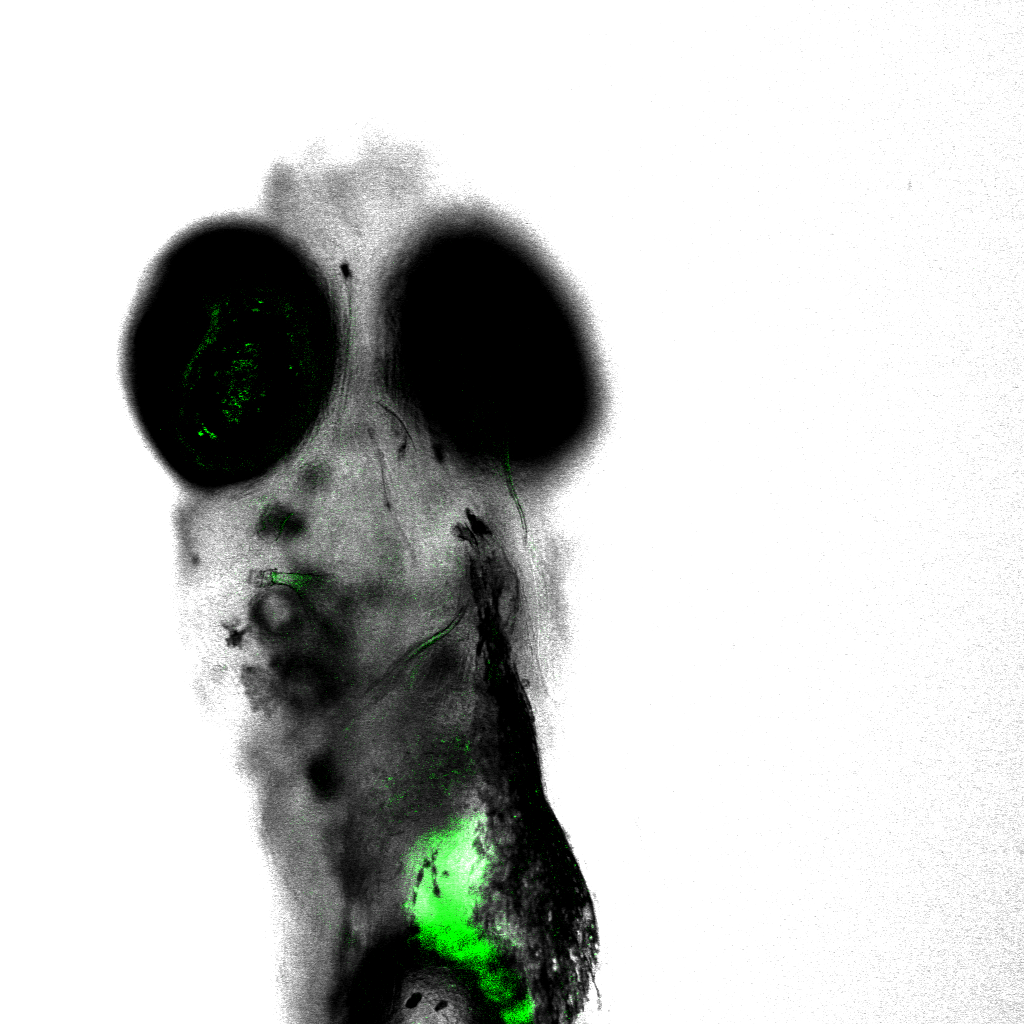

Supplement: Supplementary file 7 [file DataSheet2.zip › Original date(Figure 7-1)/SCH-Fluo 4-calcium ion/SCH23390-2_.tif]

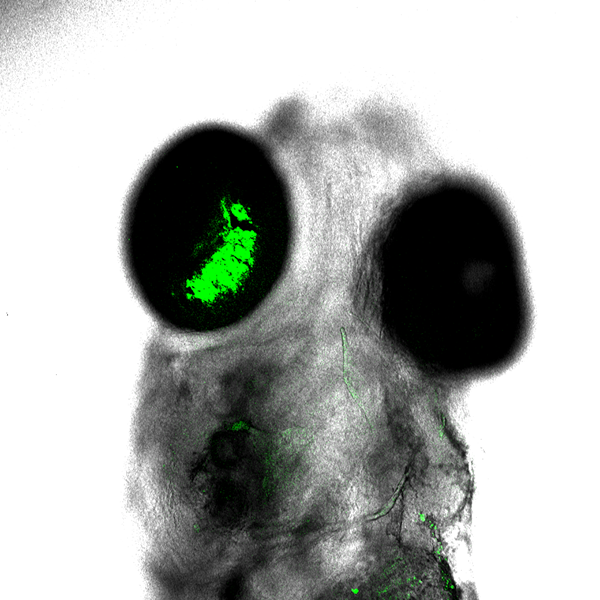

Supplement: Supplementary file 7 [file DataSheet2.zip › Original date(Figure 7-1)/SCH-Fluo 4-calcium ion/SCH23390-3.tif]

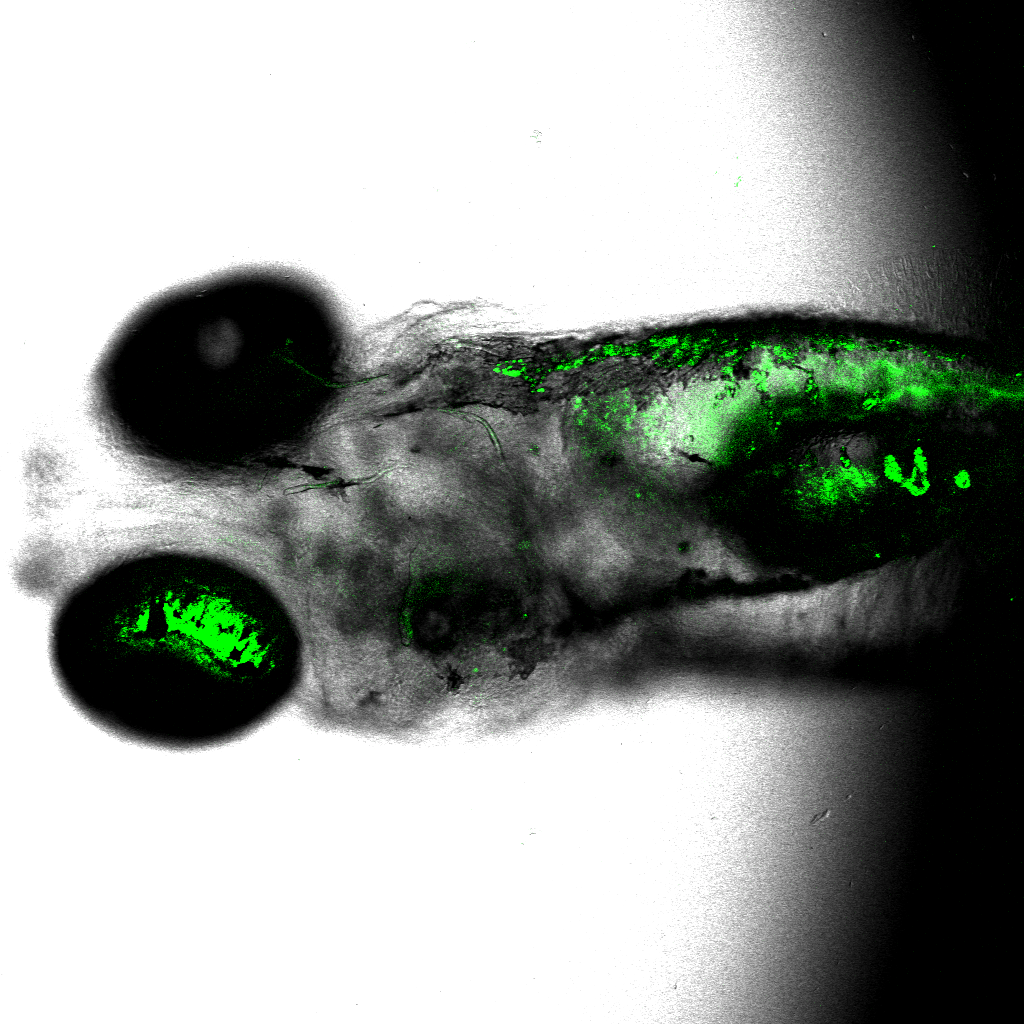

Supplement: Supplementary file 7 [file DataSheet2.zip › Original date(Figure 7-1)/SCH-Fluo 4-calcium ion/SCH23390-4.tif]

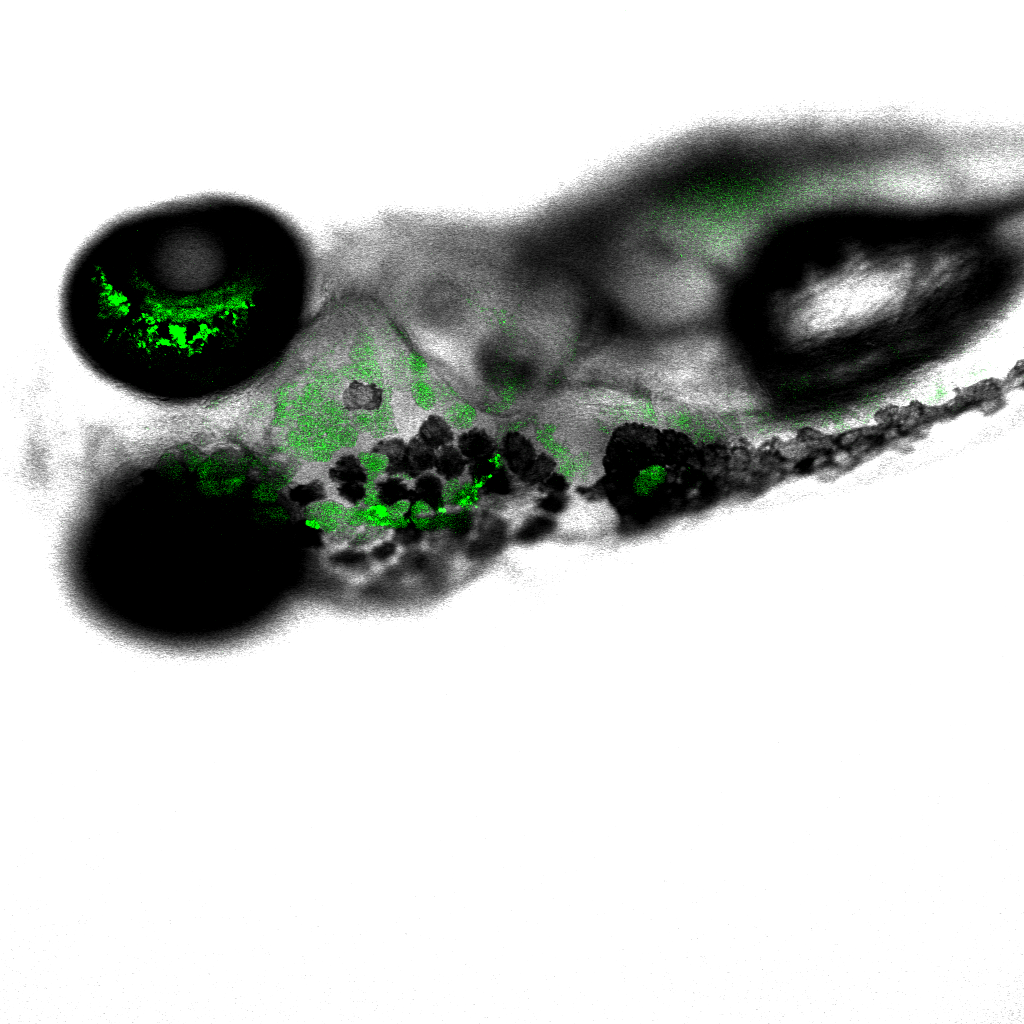

Supplement: Supplementary file 7 [file DataSheet2.zip › Original date(Figure 7-1)/Sumanirole-Fluo 4-calcium ion/Aconitine-1.tif]

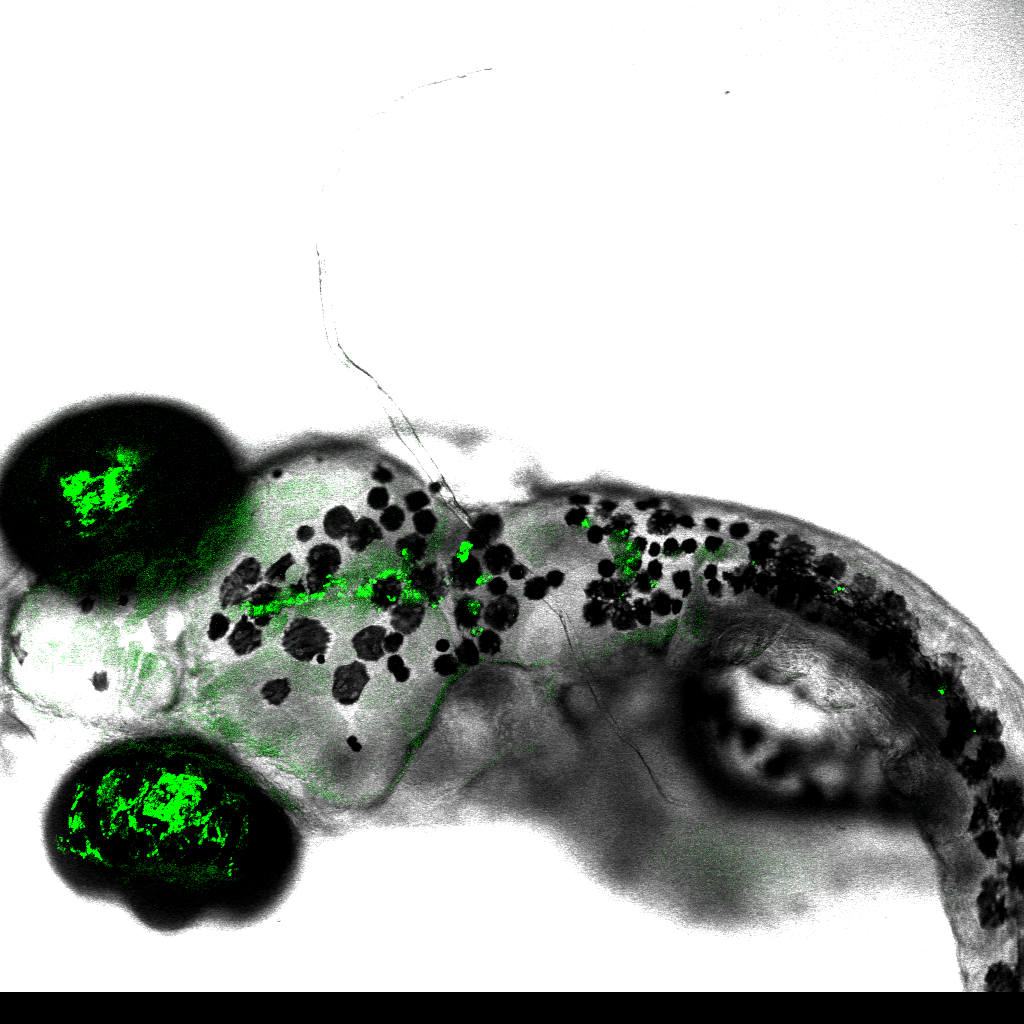

Supplement: Supplementary file 7 [file DataSheet2.zip › Original date(Figure 7-1)/Sumanirole-Fluo 4-calcium ion/Aconitine-2_.tif]

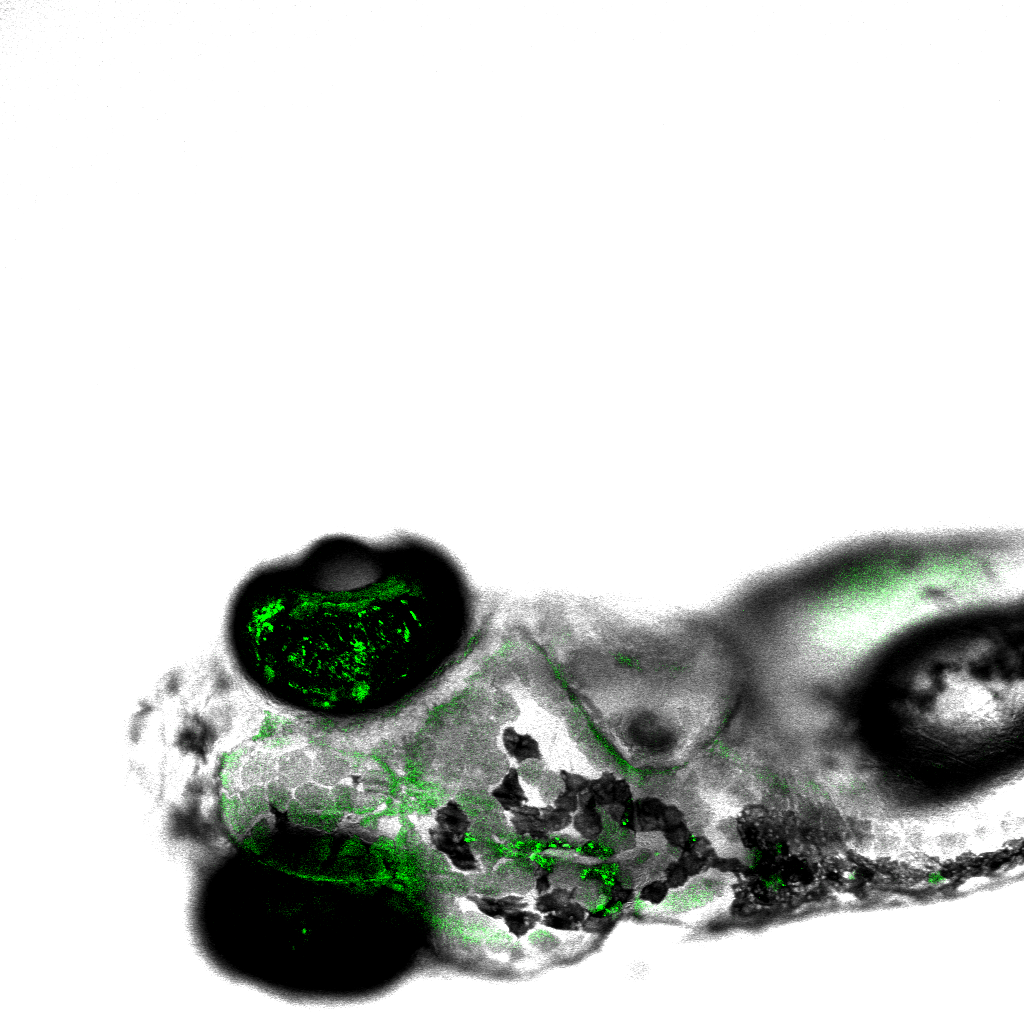

Supplement: Supplementary file 7 [file DataSheet2.zip › Original date(Figure 7-1)/Sumanirole-Fluo 4-calcium ion/Aconitine-3_.tif]

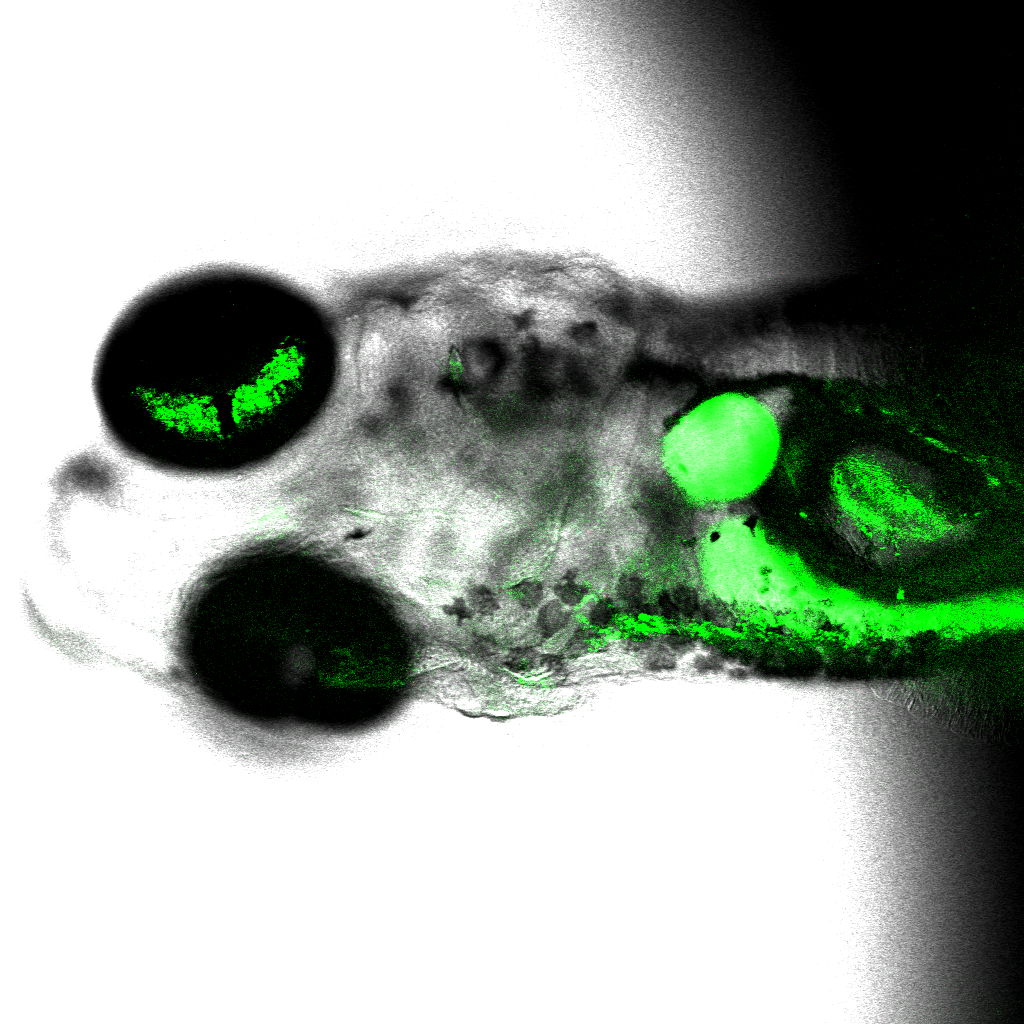

Supplement: Supplementary file 7 [file DataSheet2.zip › Original date(Figure 7-1)/Sumanirole-Fluo 4-calcium ion/C-1.tif]

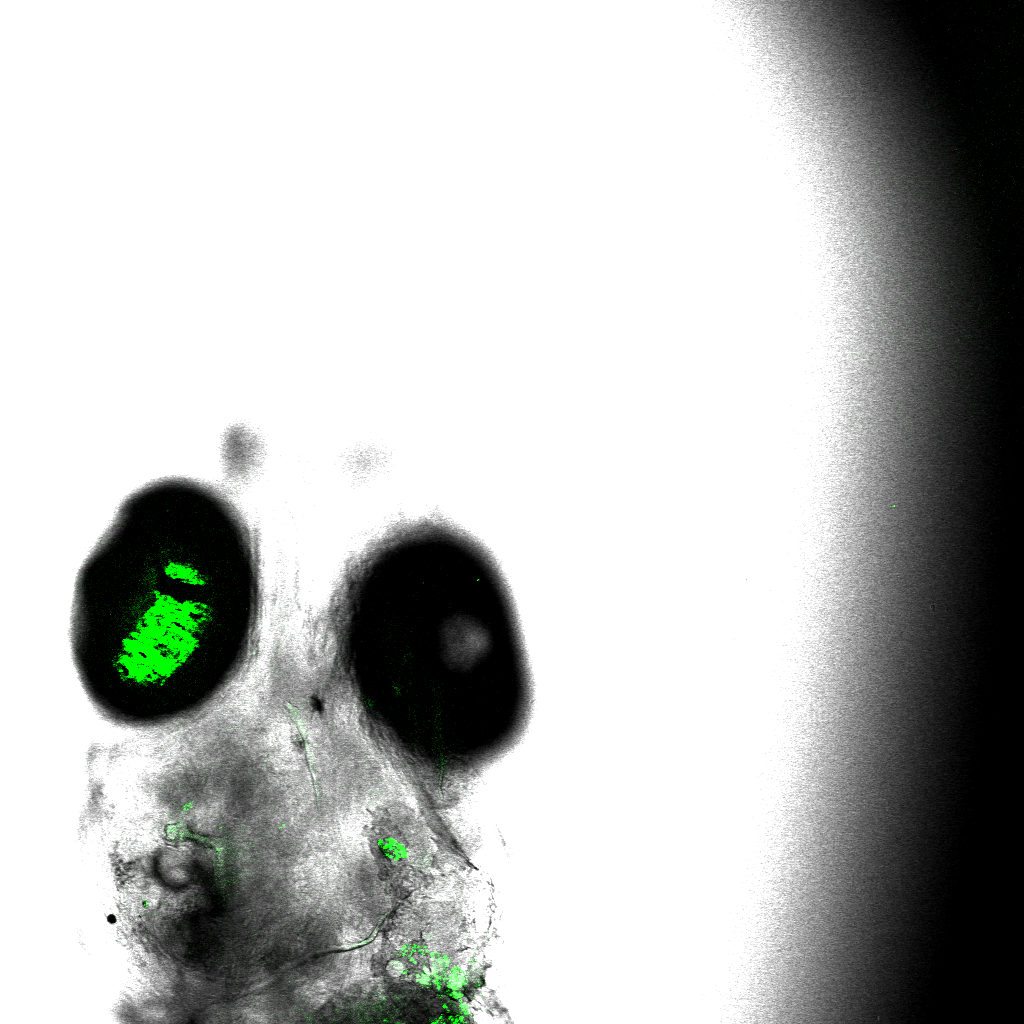

Supplement: Supplementary file 7 [file DataSheet2.zip › Original date(Figure 7-1)/Sumanirole-Fluo 4-calcium ion/c-2_.tif]

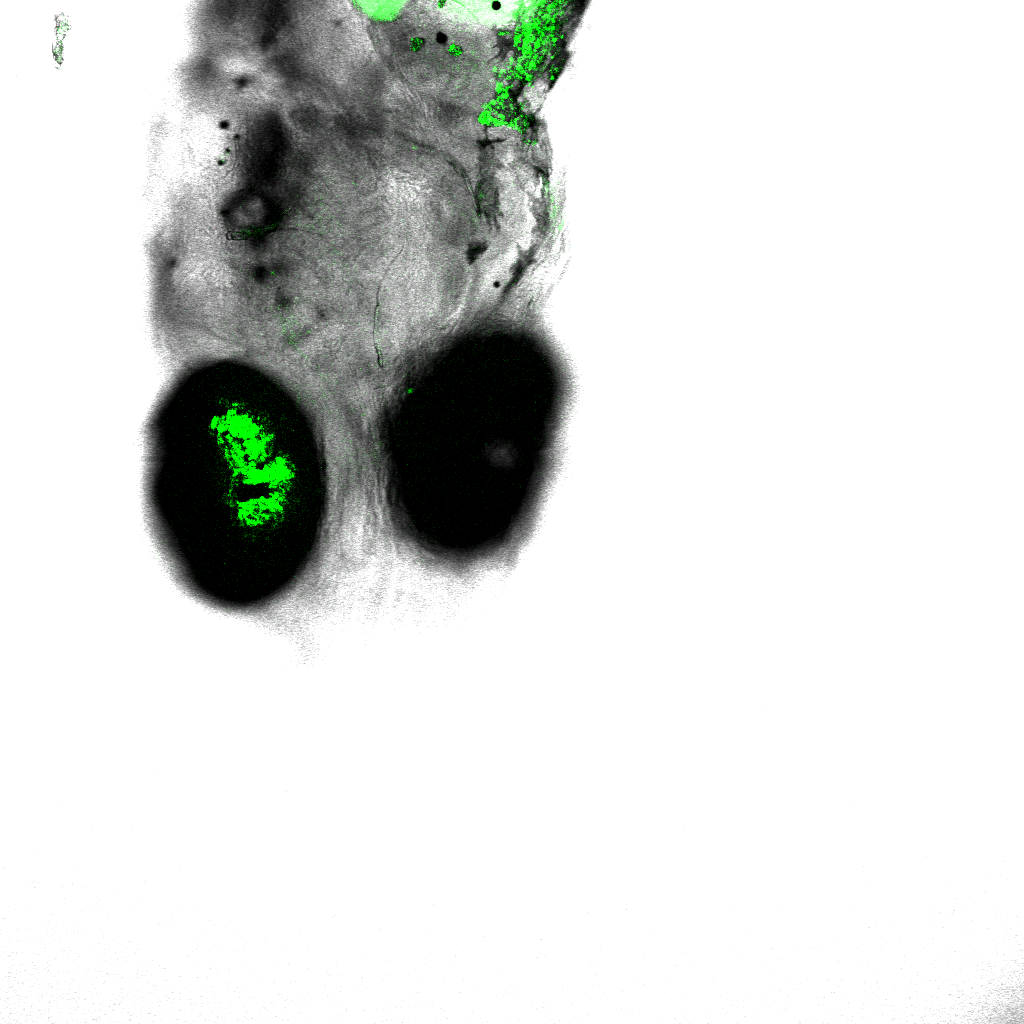

Supplement: Supplementary file 7 [file DataSheet2.zip › Original date(Figure 7-1)/Sumanirole-Fluo 4-calcium ion/C-3.tif]

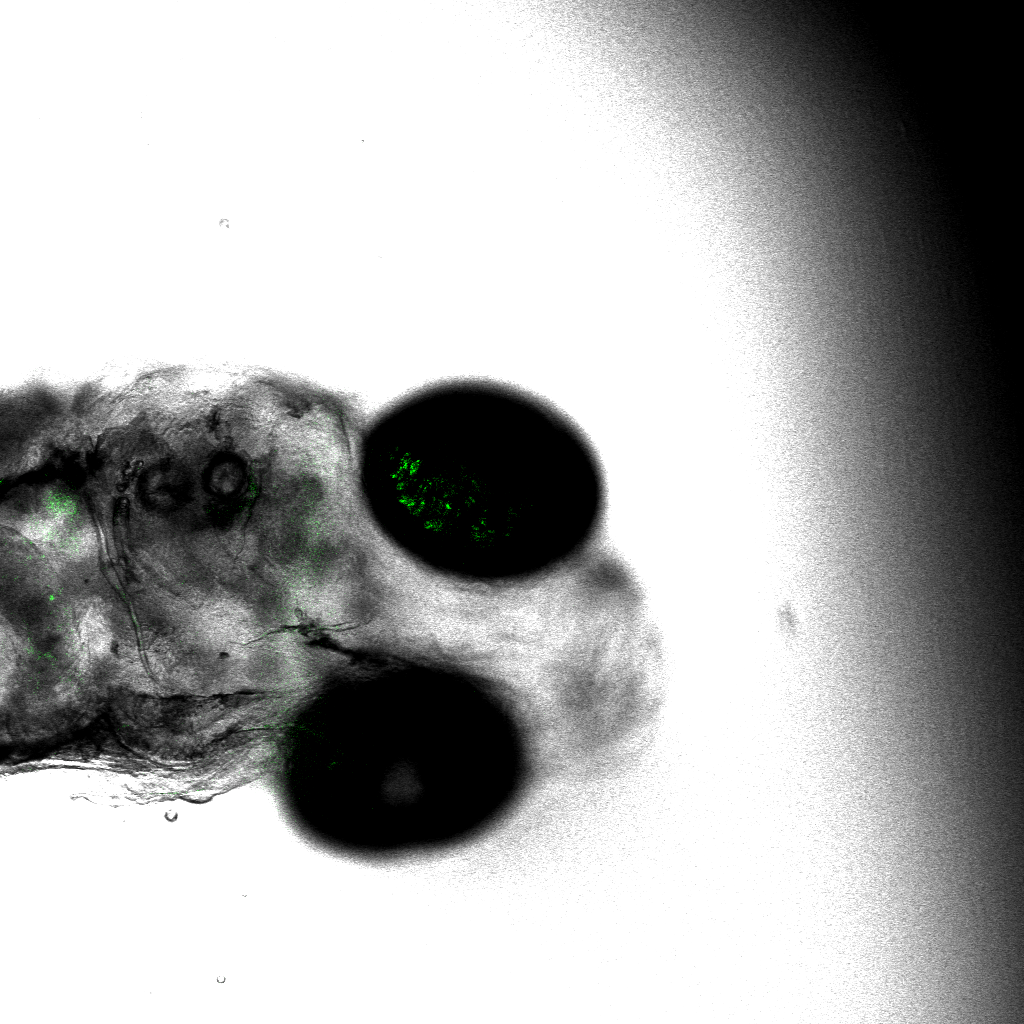

Supplement: Supplementary file 7 [file DataSheet2.zip › Original date(Figure 7-1)/Sumanirole-Fluo 4-calcium ion/sum-1_.tif]

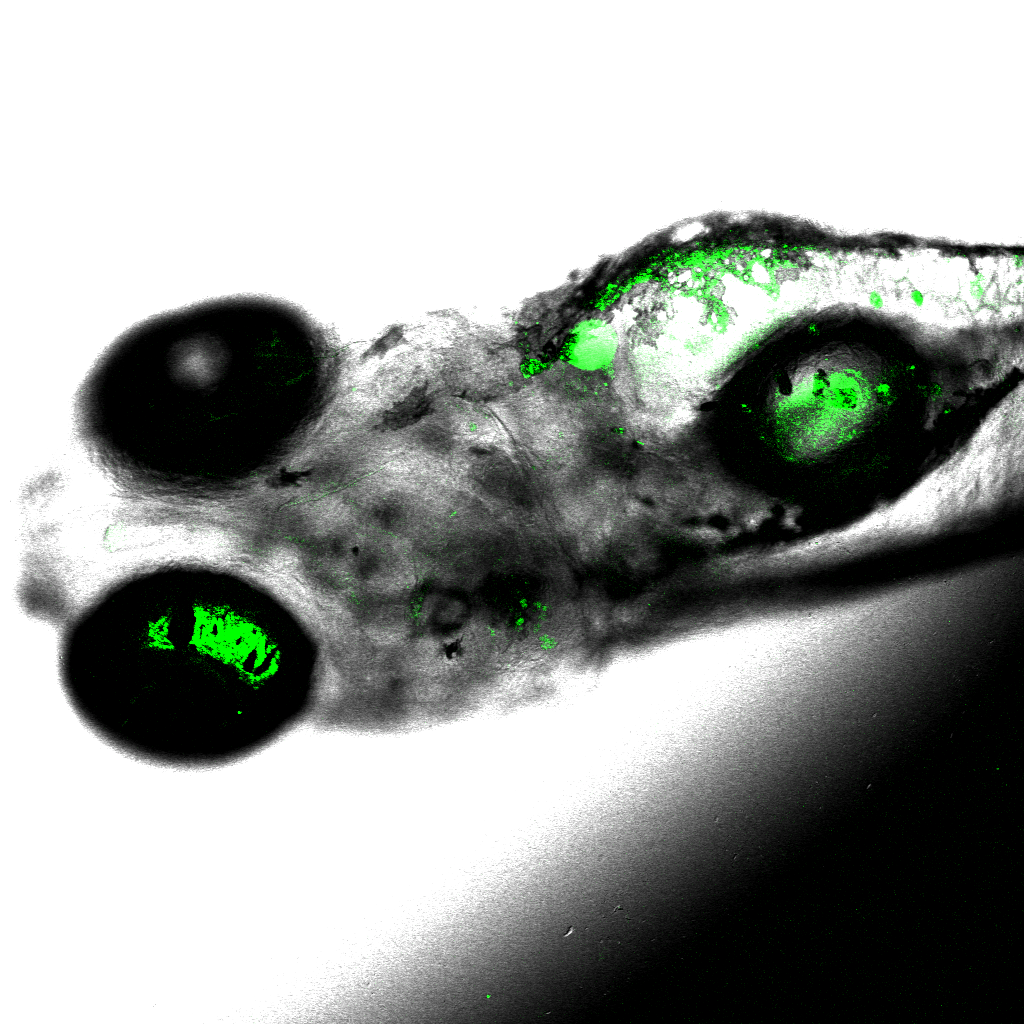

Supplement: Supplementary file 7 [file DataSheet2.zip › Original date(Figure 7-1)/Sumanirole-Fluo 4-calcium ion/sum-2_.tif]

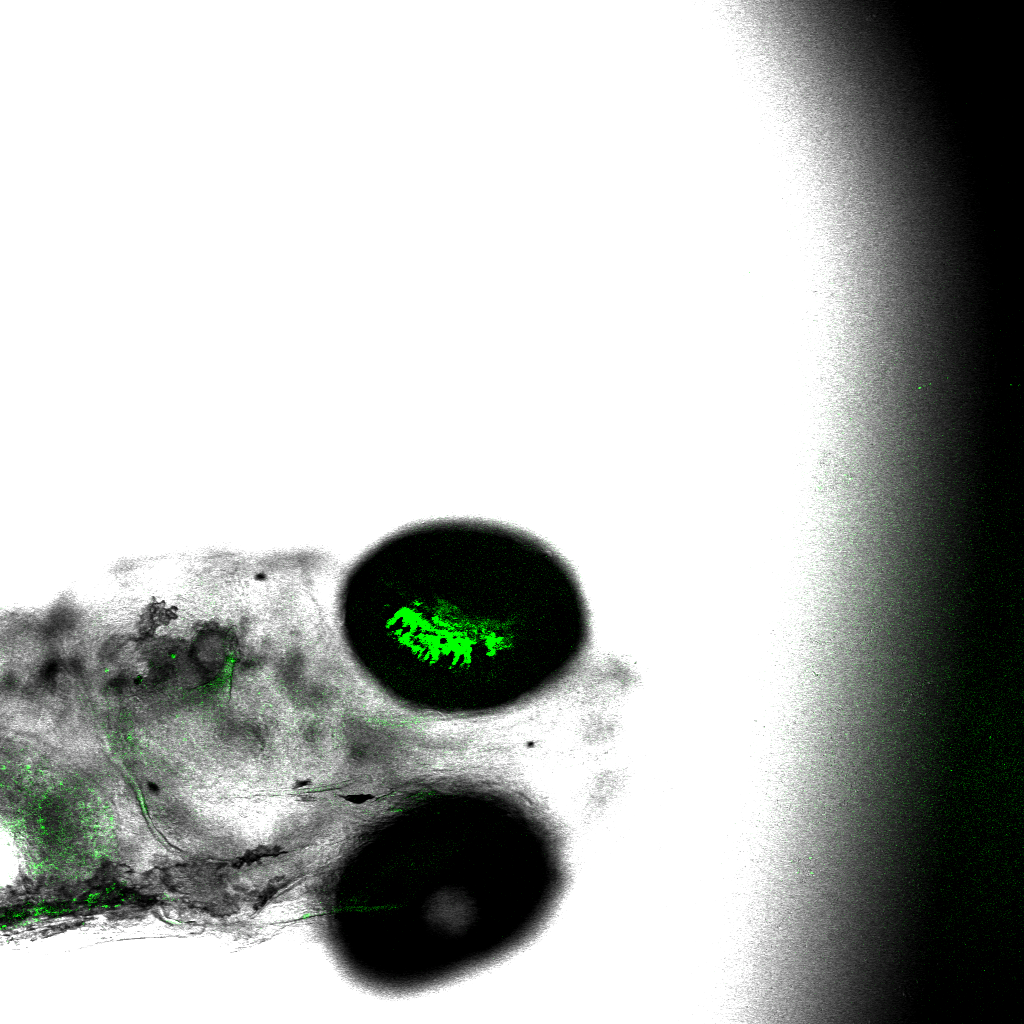

Supplement: Supplementary file 7 [file DataSheet2.zip › Original date(Figure 7-1)/Sumanirole-Fluo 4-calcium ion/sum-3_.tif]

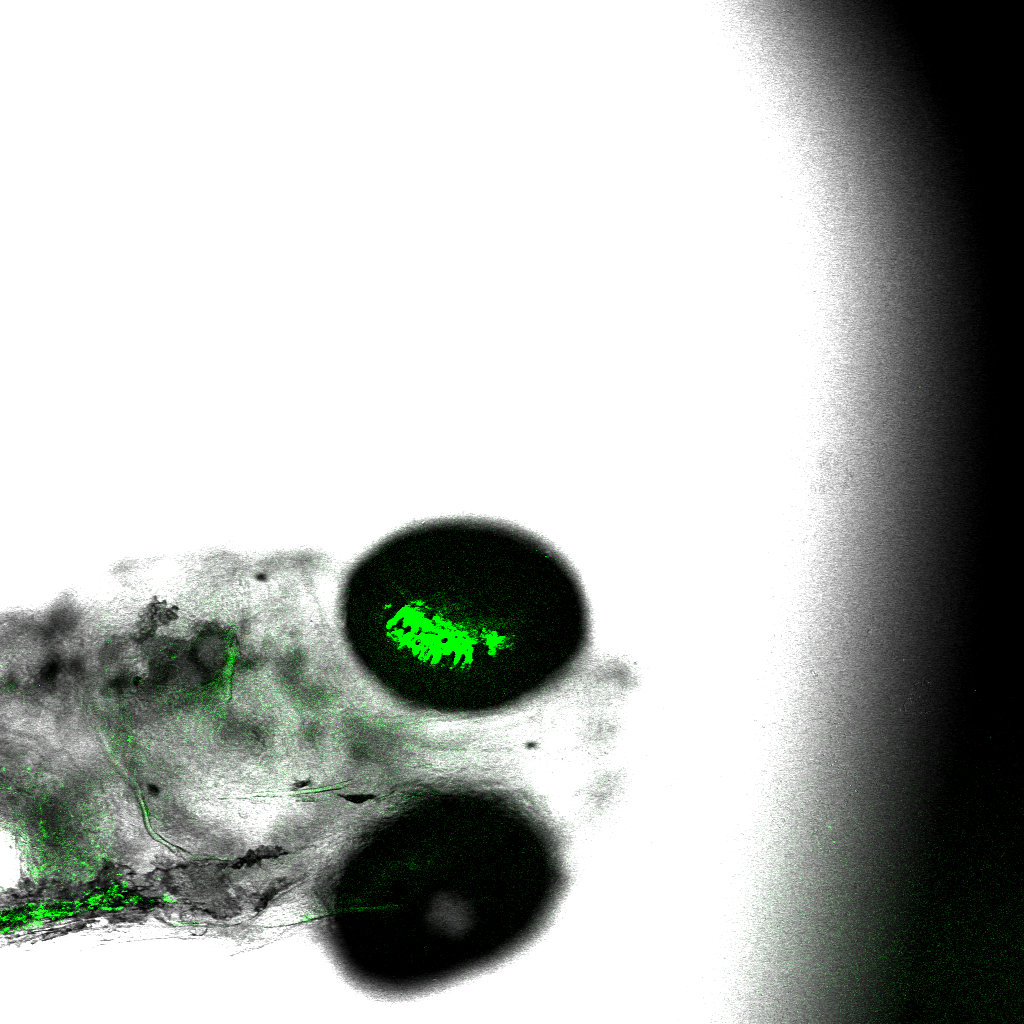

Supplement: Supplementary file 7 [file DataSheet2.zip › Original date(Figure 7-1)/Sumanirole-Fluo 4-calcium ion/Sumanirole+Aconitine-1_.tif]

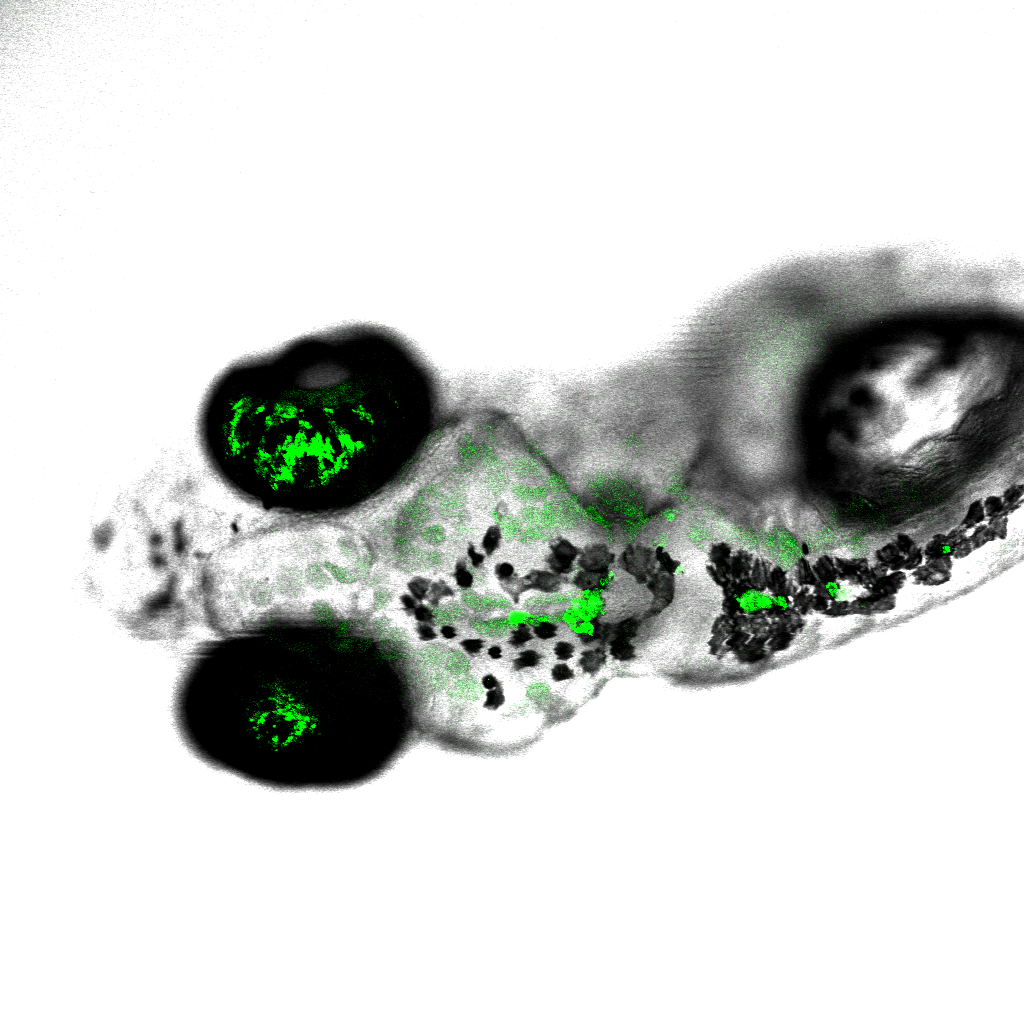

Supplement: Supplementary file 7 [file DataSheet2.zip › Original date(Figure 7-1)/Sumanirole-Fluo 4-calcium ion/Sumanirole+Aconitine-2_.tif]

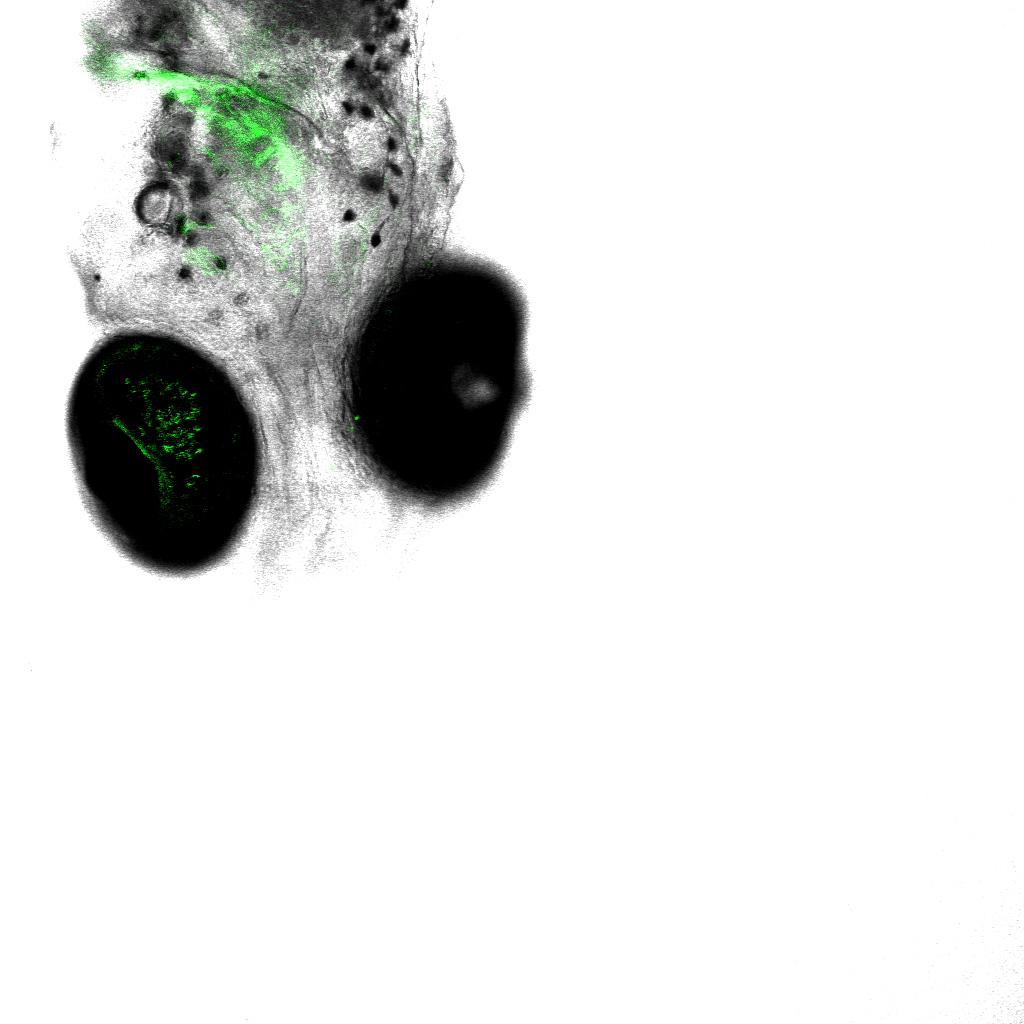

Supplement: Supplementary file 7 [file DataSheet2.zip › Original date(Figure 7-1)/Sumanirole-Fluo 4-calcium ion/Sumanirole+Aconitine-3_.tif]
