# Supplementary material for: Dopamine Homeostasis Imbalance and Dopamine Receptors-Mediated AC/cAMP/PKA Pathway Activation are Involved in Aconitine-Induced Neurological Impairment in Zebrafish and SH-SY5Y Cells
Source: Front Pharmacol. 2022 Mar 18;13:837810. doi: 10.3389/fphar.2022.837810 (PMC8971779; doi:10.3389/fphar.2022.837810)
Supplement: Supplementary file 2 [file Table1.DOCX]

**Table S1 Primer sequences used for the** **RT-PCR.**

| **Gene names** | **Species** | **Forward sequences (5’−3’)** | **Reverse sequences (3’−5’)** |
| --- | --- | --- | --- |
| **th1** | Zebrafish | TGAAACCAGACCCAGCCGAAAAC | CCAGCGTGCTAACATCCGACAG |
| **mao** |  | TGGCGGAGGCATCTCAGGTC | TTCTTCCACCAACACGGCTTCTG |
| **dat** |  | CCGCTCTACGCCTTCTACAAGTTC | AGGTGATGGTCAGTCTCAGGAGTG |
| **vmat2** |  | GGTGTTGGCTCGTCCTGTTCTTC | CGTTTCCTCGTTCCTCGTCATCTG |
| **drd1a** |  | GTGTTATCAGCGTGGACCGATACTG | GATGAAGGAGATGAGCACAGACAGAG |
| **drd1b** |  | AGCCCAGACGACCAGTTATACAGAG | AGCGAGGAGGAGATGGCGTATG |
| **drd2a** |  | CAGGATCAGTCTGGTGGAAGTCAAC | CTTCAGGCGATGGAGGTGATATGTC |
| **drd2b** |  | GTTGTCTACTCCTCCATCATGTCGTTC | TCCGTTTAGTGTTGACCCGTTTCC |
| **drd2l** |  | CCAGACCACCACCAATTACCTCATC | CACTCACCAACAACCTCCAGATAGAC |
| **drd3** |  | CAGGCGTTGAAGAAGCGAAGAGG | AGGTAGTTGGTGGTGGTCTGAAGAG |
| **drd4a** |  | TATGCGGAGTTTCAGGATGGAGTTTG | TTAGAGGGATGGAGACGGCGATG |
| **drd4b** |  | AAGCCCTCAAGACGACGACAAAC | TCCATCACAGACAGACACATTCAAGG |
| **drd4-rs** |  | TTTACTCCTCCGTTTGCTCCTTCTTC | CATCGCTTGAGTCCTCTGAACATCC |
| **drd5a** |  | CTGGACCAACTCTTCGCTCAACC | TCGTTGCTGATGTTGACCGTCTC |
| **β-actin** |  | TGAATCCCAAAGCCAACAGAGAGAAG | CCATCACCAGAGTCCATCACAATACC |
| **th** | SH-SY5Y  cells | TGTCTGAGGAGCCTGAGATTCGG | TTGTCCTTGGCGTCACTGAAGC |
| **mao-b** |  | ACAAATGCGACGTGGTCGT | TCCTCTCCTGTCCTCCATTGGTTG |
| **dat** |  | CTACCGACTCTGTGAGGCATCTG | CCACGACCACGAACAGGAGAAAG |
| **vmat2** |  | GTCCTTCTGCTGGTGGTGCTATTG | TTCTTCTTTGGCAGGTGGACTTCG |
| **drd1** |  | GGTGACCAACTTCTTTGTCATC | GGGGTCATCTTTCTCTCATACC |
| **drd2** |  | CCACTACAACTACTATGCCACA | GAATCCTGCTGAATTTCCACTC |
| **drd3** |  | CTTCCAAGAAAGAGGAGGAGAG | CCATTGCTGAGTTTTCGAACTT |
| **drd4** |  | CTCTTCGTCTACTCCGAGGTCCAG | CACGAACCTGTCCACGCTGATG |
| **drd5** |  | CAACATGACCAACGTCTTCATC | CTGAGTCATCTTGCGCTTGTAG |
| **β-actin** |  | CTACCTCATGAAGATCCTCACCGA | TTCTCCTTAATGTCACGCACGATT |
