# Supplementary figures and images for: Dopamine Homeostasis Imbalance and Dopamine Receptors-Mediated AC/cAMP/PKA Pathway Activation are Involved in Aconitine-Induced Neurological Impairment in Zebrafish and SH-SY5Y Cells (part 2 of 2)
Source: Front Pharmacol. 2022 Mar 18;13:837810. doi: 10.3389/fphar.2022.837810 (PMC8971779; doi:10.3389/fphar.2022.837810)

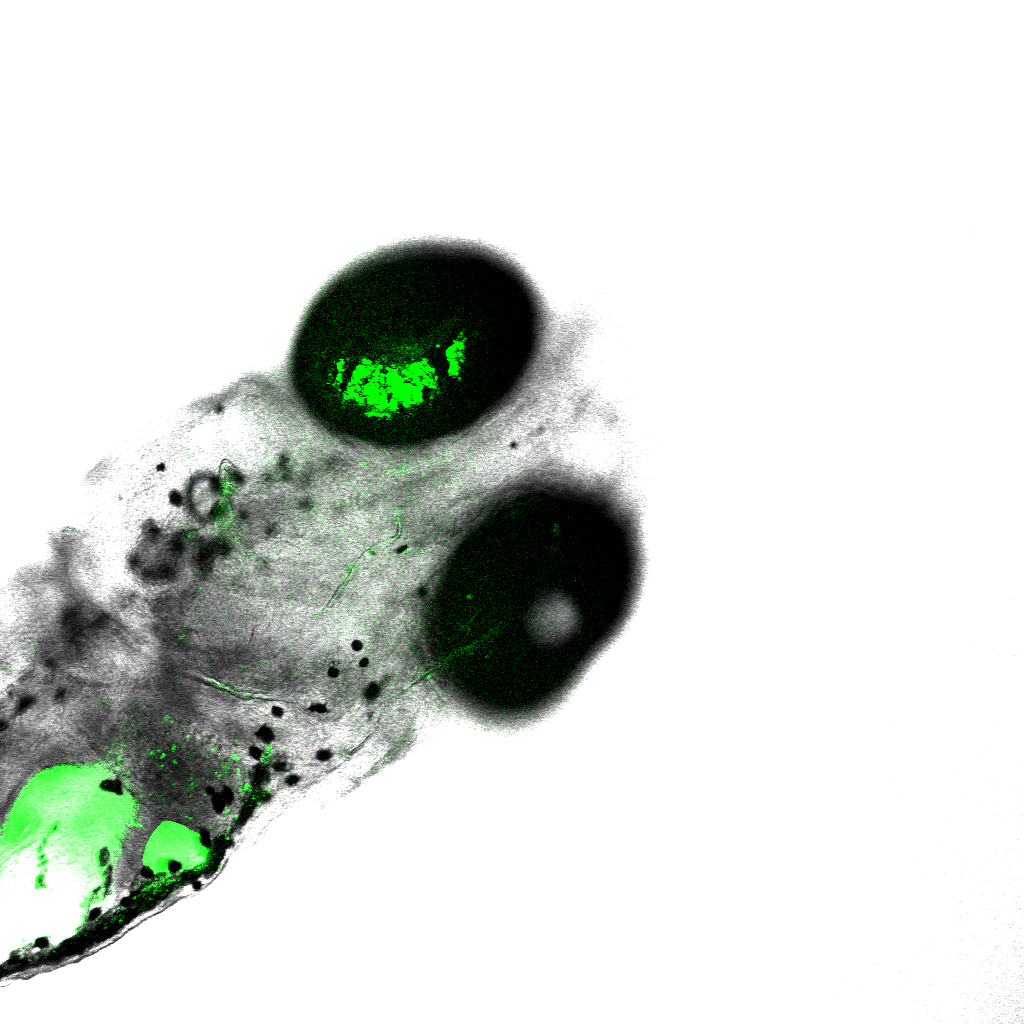

Supplement: Supplementary file 7 [file DataSheet2.zip › Original date(Figure 7-1)/Sumanirole-Fluo 4-calcium ion/Sumanirole+Aconitine-4_.tif]
